# Large-scale data-driven pre-trained DNA models enhance performance across diverse genomics tasks

**DOI:** 10.1038/s41467-026-73129-6

**Published:** 2026-05-14

**Authors:** Canzhuang Sun, Zhijie He, Shifei Zhang, Kang Xu, Yu Sun, Yuyang Wang, Pengzhen Hu, Xiaochen Bo, Mingzhi Liao, Hao Li, Hebing Chen

**Affiliations:** 1 College of Life Sciences, Center of Bioinformatics, Northwest A&F University, Yangling, China; 2 Academy of Military Medical Sciences, Beijing, China

**Keywords:** Genome informatics, Computational models, Gene regulation

## Abstract

Sequence-based deep learning has advanced genome interpretation, yet most models remain task-specific and rely on retraining, limiting scalability across biological contexts. Here we present SUCCEED, a supervised multi-task DNA foundation model pretrained on 6,389 ENCODE functional genomics tracks to learn transferable regulatory representations. By integrating convolutional layers with a Transformer architecture, SUCCEED captures both local sequence motifs and long-range regulatory dependencies, achieving performance comparable to or exceeding Enformer across benchmark tasks. Through transfer learning, it predicts cell-type-specific epigenomic profiles, denoises sparse chromatin accessibility signals, and predicts three-dimensional chromatin contacts without CTCF input across data scales and cell types. Across diverse genomics tasks, SUCCEED performs comparably to supervised foundation models such as Sei and outperforms self-supervised models trained solely on DNA sequence. Overall, SUCCEED is a transferable and scalable foundation model that provides a unified framework for genome-scale regulatory modeling in complex biological contexts.

## Abstract (web summary)

SUCCEED is a supervised multi-task DNA foundation model pretrained on 6,389 ENCODE tracks. It integrates CNN and Transformer architectures to model local motifs and long-range dependencies, enabling prediction of genomic regulatory features across tasks and cell types.

## Introduction

A long-standing goal of genomics is to decode genome sequences and elucidate the functional consequences of sequence variation^1,2^. Recent advances, including near-complete human genome assemblies and large-scale functional genomic resources across multiple scales, have substantially advanced our ability to link sequence variation to regulatory mechanisms and cellular states, and have mapped gene regulation at unprecedented resolution^3–6^. However, current datasets remain limited in their coverage across tissues, cell types, and spatiotemporal contexts, and sequencing or experimental noise further constrains signal quality. Consequently, existing resources capture only partial aspects of transcriptional regulatory networks, impeding quantitative modeling of complex mechanisms and limiting systematic assessment of the pathogenic impact of noncoding variants.

Deep learning has emerged as a powerful tool for genomics, reducing reliance on experimental data and enabling the direct prediction of gene expression, chromatin accessibility, histone modifications, transcription factor (TF) binding, and 3D chromatin architecture from DNA sequences^7–15^. Trained models can further be used to reveal cis-regulatory grammar and to assess the potential impact of noncoding variants. However, existing sequence-based approaches generally lack cell-type-specific signals, limiting their ability to perform accurate de novo prediction in unseen cell types and often requiring computationally intensive retraining when applied to new datasets^16–20^. Although integrating multiple epigenomic datasets can improve predictive performance, the generalization of such methods remains limited. Genomic language models, such as DNABERT, Nucleotide Transformer, HyenaDNA, and Evo, have demonstrated superior performance to task-specific models across diverse genomic tasks^21–25^. However, these models rely solely on masked self-supervised training of genomic sequences and thus fail to exploit the wealth of available epigenomic resources. Moreover, most current methods depend on task-specific architectures, necessitating repeated retraining across applications, which increases computational costs and fails to fully exploit large-scale genomic data.

To address these challenges, we present SUCCEED (Sequence-Functional Genome Foundation Model), a data-driven, transferable, and scalable DNA foundation model. Trained in a supervised manner on large-scale functional genomic datasets, SUCCEED directly learns the mapping between DNA sequences and regulatory features, capturing the regulatory grammar underlying transcriptional regulation. Through transfer learning, SUCCEED demonstrates strong generalization across diverse downstream tasks, including prediction of cell-type-specific epigenomic profiles, denoising and enhancement of chromatin accessibility data, and inference of 3D chromatin interactions. Overall, SUCCEED provides a unified framework for multi-task, multimodal, and multiscale genomic analysis, achieving strong performance across diverse genomics tasks.

## Results

### Overview of SUCCEED

To model cis-regulatory grammar at multiple scales, we designed SUCCEED as a hybrid architecture inspired by Enformer^12^, Borzoi^21^, and AlphaGenome^22^ that integrates local and distal sequence information in a hierarchical manner (Supplementary Fig. 1). Specifically, the architecture consists of four main components: a convolutional frontend for early motif extraction, a multi-stage downsampling convolutional tower, a Transformer encoder for modeling long-range dependencies, and a pointwise prediction head with multitask outputs (“Methods”). A one-dimensional convolutional neural network identifies conserved local sequence motifs within cis-regulatory elements, such as promoters and enhancers. The downsampling tower enables the model to progressively reduce sequence resolution, allowing it to integrate information from different genomic regions effectively. These local features are passed to a Transformer module, which captures long-range dependencies by modeling interactions between distal genomic regions. Finally, a pointwise multi-task output head is used to support concurrent prediction of multiple regulatory signals, including chromatin accessibility, enhancer activity, and TF binding. This hierarchical design enables joint encoding of local motif signals and the long-range regulatory context, thereby enhancing the model’s ability to represent gene regulatory logic. To reduce computational cost, we optimized several architectural components by decreasing the number of convolutional kernels while increasing kernel stride to more effectively capture sequence motifs. In addition, motivated by recent advances in large language models, we systematically optimized key components of our architecture^12,26,27^. We incorporated the SwiGLU activation into the Transformer modules, where its gating mechanism adaptively modulates information flow, substantially enhancing representational flexibility and expressive capacity compared with piecewise-linear activations such as ReLU^28^. In parallel, we replaced conventional LayerNorm with RMSNorm. By normalizing activations solely based on their root mean square, RMSNorm eliminates mean subtraction, reduces computational overhead, and preserves the directional information of representations, thereby improving training stability in large-scale Transformer models^29^. Furthermore, we introduced rotary positional embeddings (RoPE) to jointly encode the relative and absolute positional information of regulatory elements within sequences, strengthening the model’s ability to capture and exploit sequence structure^30^.

**Fig. 1:**
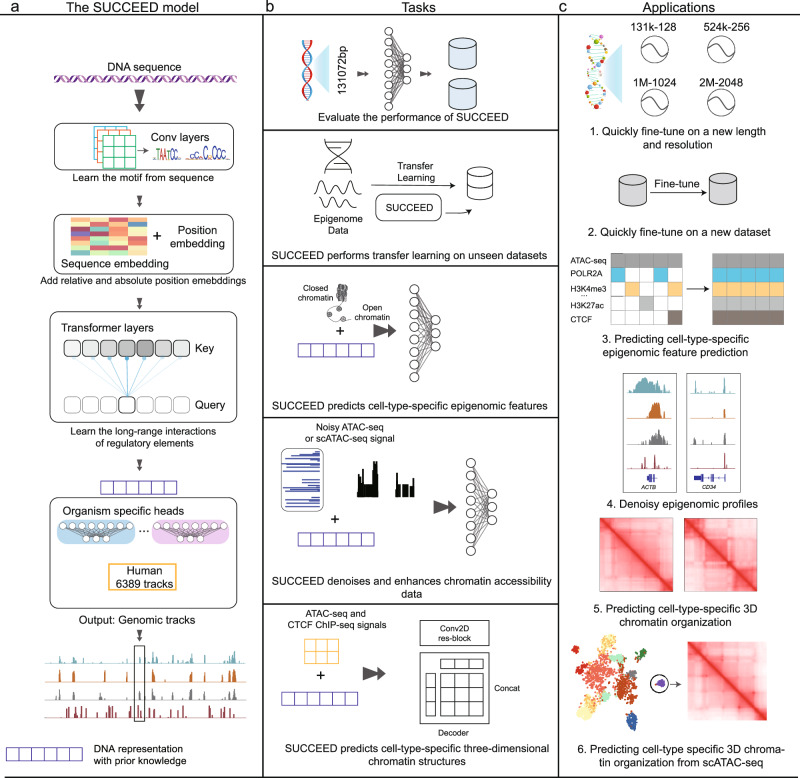
Schematic of the SUCCEED workflow. **a** Pre-trained framework of the SUCCEED model. Multiscale DNA sequences were encoded using one-hot encoding and used as input to predict 6389 human epigenomic tracks. **b** The performance of the SUCCEED model was evaluated on an independent test set, followed by the application of transfer learning and additional information processing to address various complex genomic tasks. Benchmarking was performed against several state-of-the-art models. **c** The sequence representations learned by SUCCEED, incorporating prior knowledge, were applied to a range of genomic downstream tasks, including adaptation to new datasets, prediction of cell-type-specific epigenomic modifications, denoising and enhancement of chromatin accessibility data, and prediction of cell-type-specific chromatin 3D structures.

SUCCEED was pretrained to model the relationship between DNA sequence and diverse epigenomic features using 6389 epigenomic tracks curated from the ENCODE resource, spanning multiple tissues and cell types. These tracks encompass chromatin accessibility, histone modifications, and TF binding (Supplementary Data 1)^31,32^. The model was trained in a supervised multi-task framework, taking one-hot encoded DNA sequences as input and producing predictions through 6389 task-specific output heads, thereby enabling broad inference across heterogeneous epigenomic signals (Fig. 1). Training was optimized using a Poisson negative log-likelihood loss, which effectively captures quantitative associations between DNA sequence and a wide range of epigenomic readouts^33^. This large-scale supervised pretraining provides the foundation for all downstream evaluations.

To address diverse genomic tasks, we designed transfer strategies, including efficient fine-tuning schemes such as updating only the final classification head. By incorporating widely available chromatin accessibility data, the model can predict additional cell-type-specific epigenomic features, including histone modifications, TF binding, and 3D chromatin architecture. Furthermore, leveraging prior knowledge learned during large-scale pretraining, SUCCEED effectively denoises low-quality and noisy ATAC-seq data, including scATAC-seq.

### Efficient and accurate genomic prediction with SUCCEED

**Fig. 2:**
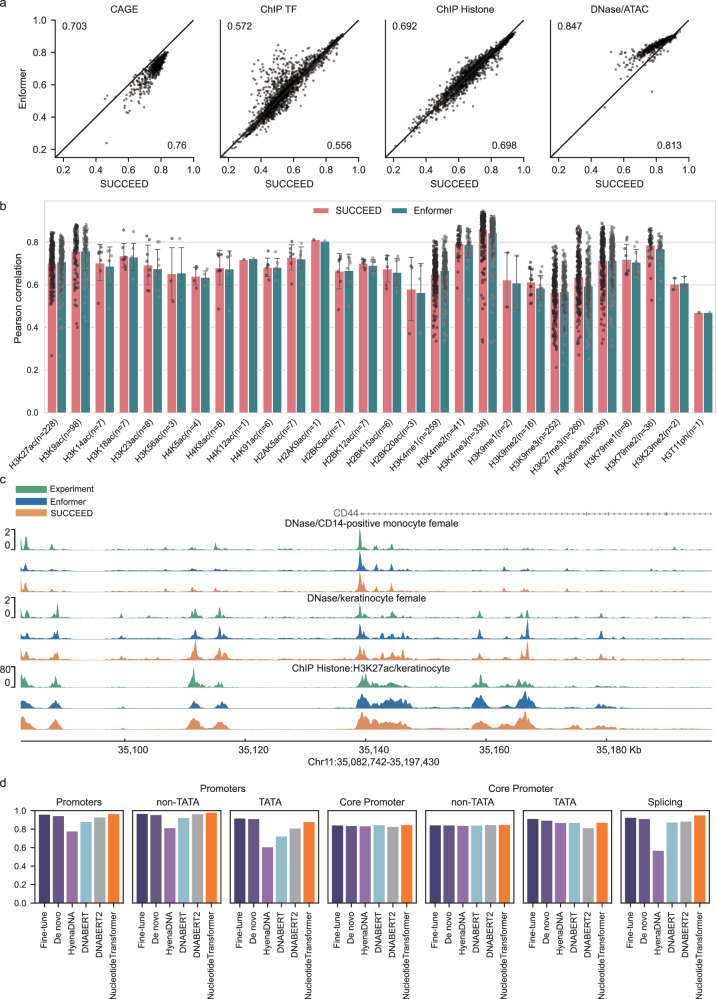
Model performance evaluation. **a** Comparison of SUCCEED and Enformer results across 638 CAGE, 2131 transcription factor (TF) ChIP-seq, 1860 histone modification ChIP-seq, and 684 DNase-seq or ATAC-seq datasets. **b** Pearson correlation coefficients between SUCCEED and Enformer across multiple histone modification datasets. The *x*-axis indicates individual histone modification marks, and the *y*-axis shows the mean Pearson correlation coefficient. Data are presented as mean ± s.d., where *n* represents the number of samples. **c** Representative examples of predicted genomic trajectories at the *CD44* locus in the test set, illustrating the consistency between SUCCEED and Enformer predictions and the true results. The three trajectories in each experiment share the same *y*-axis. **d** SUCCEED was benchmarked against masked self-supervised genomic language models across seven representative genomic tasks, with de novo referring to models trained from scratch on a given task without leveraging pretrained parameters.

We first assessed model performance under a DNA-only setting by benchmarking SUCCEED against Enformer on an independent held-out test set (“Methods”). Despite using substantially fewer convolutional kernels and Transformer layers, SUCCEED achieved comparable accuracy. For CAGE signal prediction, SUCCEED reached a Pearson correlation coefficient (PCC) of 0.76, outperforming Enformer (0.703). In TF ChIP-seq tasks, SUCCEED attained a PCC of 0.556, slightly below Enformer (0.572). For histone modification ChIP-seq, SUCCEED achieved 0.698, nearly identical to Enformer (0.692). In DNase-seq and ATAC-seq tasks, SUCCEED reached 0.813, marginally lower than Enformer (0.847) (Fig. 2a).

Notably, these results indicate that SUCCEED maintains competitive performance across diverse assay types despite its reduced architectural complexity. Analysis of individual histone marks further confirmed this overall trend, with performance differences remaining small and consistent across marks (Fig. 2b). In addition, at the *CD44* gene locus in the human hg38 reference genome, both SUCCEED and Enformer show strong agreement with experimental measurements across multiple epigenomic tracks, indicating that SUCCEED accurately captures locus-specific regulatory patterns (Fig. 2c).

We benchmarked SUCCEED against existing DNA foundation models on seven widely used genomic tasks that are commonly employed for model evaluation. Specifically, the tasks include promoter prediction, non-TATA promoter prediction, TATA promoter prediction, core promoter prediction, non-TATA core promoter prediction, and TATA core promoter prediction, all formulated as binary classification problems, as well as human splice-site prediction, which is treated as a multi-task classification task. Across all seven tasks, SUCCEED was evaluated on identical datasets under both fine-tuning and training from scratch settings (“Methods”). The fine-tuned SUCCEED model achieved a mean accuracy of 0.906, compared to 0.891 for the model trained from scratch. This result suggests a modest improvement associated with large-scale supervised pretraining for genomic prediction tasks (Fig. 2d). We compared SUCCEED with DNA foundation models on these tasks and found that it achieves performance comparable to, or exceeding, that of larger self-supervised models across most genomic benchmarks (Fig. 2d and “Methods”).

We next examined whether SUCCEED learns biologically meaningful representations rather than spurious correlations through a systematic interpretability analysis of the pre-trained model. Specifically, we extracted activation maps from the first convolutional layer and aggregated subsequences whose activation values exceeded 80% of the maximal response, from which we derived position weight matrices (PWMs) for each convolutional filter. The resulting sequence patterns were then matched to known TF motifs using TOMTOM from the MEME suite^34^ against the JASPAR database^35^, enabling the annotation of individual filters. This analysis identified multiple TF motifs associated with functional regulatory elements (Supplementary Fig. 3a). In addition, to evaluate whether SUCCEED exploits long-range genomic interactions during prediction, we applied the Input × Gradient attribution method^36^. The results show that SUCCEED relies not only on local sequence features but also substantially incorporates long-range genomic information, indicating that the model captures regulatory signals across genomic scales (Supplementary Fig. 3b).

Collectively, these results indicate that supervised pretraining on large-scale functional genomics data enables the model to learn representations directly linked to biological function and long-range genomic interactions, thereby enabling accurate prediction of diverse epigenomic signals.

### Scalable and transferable modeling of gene regulation across resolutions with SUCCEED

Most existing models are trained with input lengths of 10–100 kb, approximately 256 bp, scales that roughly correspond to a single nucleosome^37–39^. However, cis-regulatory elements often span multiple nucleosomes, and distal regulation can extend over megabase distances, making single-resolution modeling insufficient to capture the full complexity of hierarchical regulatory structures^40,41^. From a biological perspective, finer resolutions (for example, 128 bp) are well suited for modeling local regulatory signals such as TF binding and chromatin accessibility, which operate at the scale of individual motifs or nucleosome-sized regions. Intermediate resolutions (for example, 512–1024 bp) capture interactions between nearby regulatory elements, including enhancers and promoters, across tens to hundreds of kilobases. In contrast, coarser resolutions paired with larger input lengths enable the integration of long-range regulatory context and structural features, such as domain-scale chromatin organization and higher-order interactions spanning hundreds of kilobases to megabases. To systematically assess SUCCEED’s performance across different input lengths and corresponding resolutions, we constructed and evaluated multiple resolution-specific variants by adjusting the pooling window size.

**Fig. 3:**
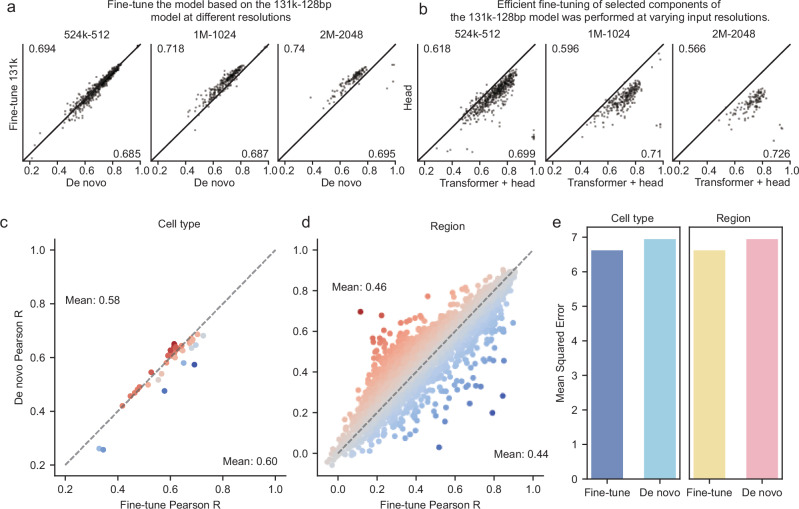
SUCCEED multi-scale input and transfer learning. **a** Performance comparison between de novo training and transfer learning for SUCCEED (using Pearson correlation coefficient). De novo refers to the model trained from scratch, while Fine-tune 131 k denotes the model fine-tuned from a pre-trained model with an input sequence length of 131,072 bp and a resolution of 128 bp. 524 k–512 refers to the model with an input length of 524,288 bp and a resolution of 512 bp; 1M–1024 corresponds to an input length of 1,048,576 bp with a resolution of 1024 bp; 2M–2048 bp indicates an input length of 2,097,152 bp and a resolution of 2048 bp. **b** Comparison of performance when fine-tuning different portions of the model. Head represents fine-tuning only the final classification head, with all other parameters frozen. Transformer + Head denotes fine-tuning both the Transformer module and the final classification head, while the remaining parameters are frozen. **c**, **d** Performance comparison on the human brain scATAC-seq dataset. De novo indicates the model trained from scratch, and Fine-tune refers to fine-tuning the 131 k model on the new dataset. **e** Mean Squared Error for the fine-tuned and pre-trained models.

We observed that SUCCEED maintained high predictive accuracy across varying input sequence lengths (Fig. 3a). Notably, fine-tuning a pretrained SUCCEED model (trained on 131,072 bp inputs) on datasets with different input lengths and resolutions (e.g., 524,288 bp) further improved epigenomic profiles prediction (Fig. 3a). These improvements were achieved without full retraining; significant gains were obtained by updating only selected components. In particular, fine-tuning the classification head while keeping other parameters fixed yielded strong performance, and jointly updating Transformer layers and the classification head outperformed models trained from scratch (Fig. 3b). Moreover, fine-tuning accelerated convergence and substantially reduced computational cost (Supplementary Fig. 4a, b). Together, these findings demonstrate SUCCEED’s adaptability in multi-resolution and multi-scale modeling of gene regulation. Moreover, with an efficient parameter-tuning strategy, SUCCEED achieves performance gains without full retraining, offering a practical solution for large-scale, multi-scale genomic data analysis.

### Transferability of SUCCEED to unseen cell types via efficient fine-tuning

Sequence-based models are commonly used to study noncoding and structural variants, learning the mapping between DNA sequence and regulatory function^12,17,19^. Most existing models are trained on specific cell types or tissues; however, adapting them to new cell types typically necessitates retraining, which is both time- and resource-intensive. Pretraining and fine-tuning strategies have been widely adopted in genomics, enabling tasks such as enhancer prediction and de novo design of regulatory sequences with defined functions using pretrained genomic language models^22,24,25,42^.

To evaluate the transferability of SUCCEED to unseen cell types, we assessed fine-tuning and training from scratch on a single-cell chromatin accessibility (scATAC-seq) dataset comprising 45 human brain cell types^43^. On the held-out test set, both strategies achieved comparable overall performance (Fig. 3c), with de novo models showing a slight advantage in certain genomic regions (Fig. 3d). Differences in mean squared error (MSE) were minimal (Fig. 3e), but fine-tuning remained more efficient in terms of training time and computational cost (Supplementary Fig. 4c). Additional analyses revealed that fine-tuned models captured cell-type-specific chromatin accessibility patterns, although limitations remained at some marker gene loci (Supplementary Fig. 4d). These results demonstrate the robust performance of SUCCEED in transfer learning settings, particularly for scATAC-seq modeling. Notably, fine-tuning only the classification head achieved predictive accuracy comparable to, or even exceeding, that of de novo models, while substantially reducing computational cost. In summary, the results demonstrate that SUCCEED has learned the grammar of transcriptional regulation from large-scale external datasets and exhibits robust generalization across data sources.

### SUCCEED enables accurate prediction of cell-type-specific epigenomic profiles

**Fig. 4:**
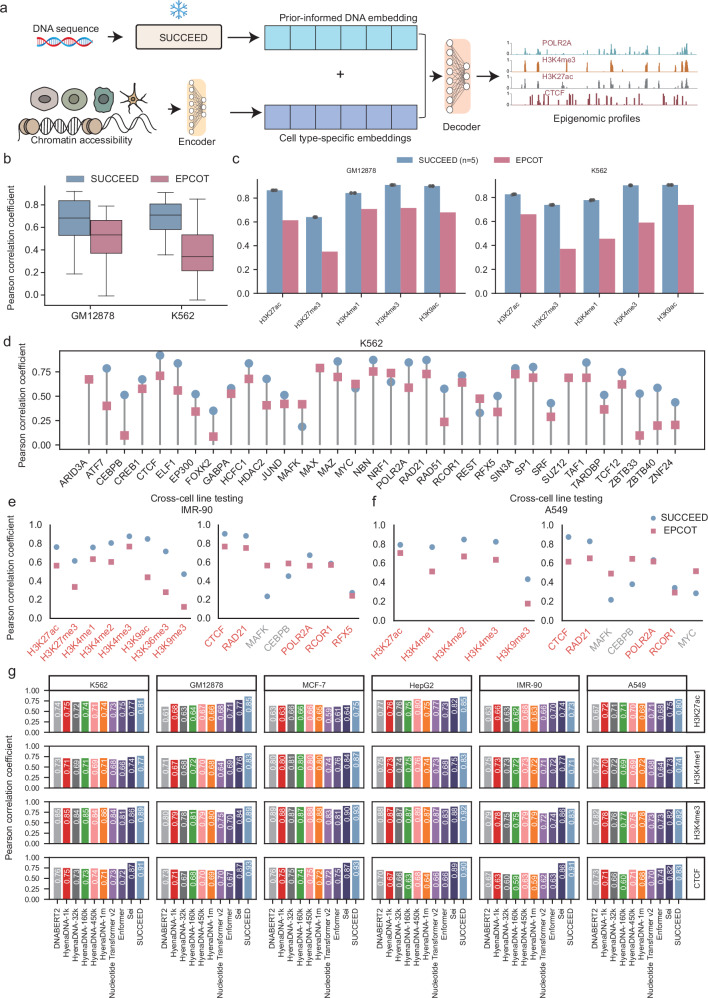
Performance of SUCCEED in predicting epigenomic profiles surpasses other models. **a** The SUCCEED framework for predicting cell-type-specific epigenomic profiles. The model takes 1,048,576 bp DNA sequences as input, incorporating cell-type-specific chromatin accessibility data. The DNA sequence is encoded through the frozen parameters of SUCCEED, while chromatin accessibility data is processed by a Conv1D-only encoder. The two representations are concatenated and input into a classification head to predict various cell-type-specific epigenomic profiles. **b** Performance comparison between SUCCEED and EPCOT on an independent test set, evaluated using the Pearson correlation coefficient. *n* = 46 samples. The box shows the interquartile range (25th–75th percentile), the centre line indicates the median, and whiskers extend to the minimum and maximum values. **c** Performance comparison between SUCCEED and EPCOT for predicting multiple histone modification profiles on independent test sets from different cell lines. The *x*-axis indicates individual histone modification marks and the *y*-axis shows the Pearson correlation coefficient. Bars represent the mean across five independent training runs (*n* = 5) with different random seeds; points show results from individual runs. **d** Performance comparison between SUCCEED and EPCOT for predicting multiple transcription factors in the K562 cell line. The *x*-axis represents transcription factors, and the *y*-axis shows Pearson correlation coefficients. **e**, **f** Performance comparison of SUCCEED and EPCOT across cell types (unseen cell lines). Histone modifications are shown on the left, and transcription factors on the right. The red areas on the *x*-axis indicate that SUCCEED outperforms EPCOT, while the gray areas represent the opposite. **g** Performance comparison of models across multiple commonly profiled epigenomic marks in different cell lines.

Predicting cell-type-specific epigenomic profiles is a central task in genomics. However, similar to most sequence-only models, the original SUCCEED model—trained exclusively on DNA sequence—has limited capacity to characterize epigenomic profiles in unseen cell types, particularly for highly cell-type-specific regulatory features. To improve SUCCEED’s generalization in this setting, we augmented its framework with an encoder that ingests cell-type-specific chromatin states. Specifically, the model extracts sequence representations through the SUCCEED backbone while processing ATAC-seq signals via a chromatin encoder, thereby integrating cell-type-specific chromatin accessibility information while preserving the core advantages of large-scale epigenomic pretraining (Fig. 4a). This architectural extension substantially improves the accuracy of cell-type-specific epigenomic profile prediction.

We evaluated SUCCEED’s performance in predicting cell-type-specific epigenomic profiles through a comprehensive comparison with the state-of-the-art method EPCOT^44^. EPCOT predicts cell-type-specific epigenomic profiles by integrating DNA sequence with chromatin accessibility or DNase-seq signals and, like SUCCEED, adopts a two-stage modeling framework. However, EPCOT is pre-trained only on local sequence contexts (1000 bp) and on a relatively limited set of genomic data, whereas SUCCEED captures both local sequence features and long-range genomic interactions and leverages large-scale functional genomics datasets for pretraining. We benchmarked the two methods under two evaluation settings: cross-chromosomal and cross-cell type. In the cross-chromosomal setting, models were trained on the cell lines K562, GM12878, HepG2, and MCF-7 and evaluated on chromosomes 10 and 21. In the cross-cell type setting, performance was assessed on held-out cell lines IMR-90 and A549.

In cross-chromosomal testing, SUCCEED outperformed EPCOT across most epigenomic marks (Fig. 4b and Supplementary Fig. 5a), with particularly notable improvements in histone modification prediction (Fig. 4c and Supplementary Fig. 5b). SUCCEED also achieved superior performance on most TF binding marks (Fig. 4d and Supplementary Fig. 5c). In cross-cell type testing on IMR-90, it yielded higher accuracy across histone marks and TFs, except MAFK and CEBPB, while both models performed similarly on structural TFs such as RCOR1 and RFX5 (Fig. 4e). Similar results were observed in A549 cell line, with SUCCEED outperforming EPCOT in histone modifications prediction but slightly underperforming on MAFK, CEBPB, and MYC (Fig. 4f). At a representative locus on chromosome 12, SUCCEED recapitulated H3K27ac regulatory signals with high concordance to experimental data (Supplementary Fig. 5d).

Additionally, we also compared SUCCEED with EPCOT using the methodology from the original EPCOT paper, which aggregates epigenomic signals shared between pairwise combinations of four cell types. This configuration increases the number of training signals tracks from 46 to 208. Under this configuration, SUCCEED also maintained superior performance, achieving higher PCC (Supplementary Fig. 6a). It outperformed EPCOT on most histone marks and achieved stronger TF binding predictions in both cross-chromosomal and cross-cell type evaluations (Supplementary Fig. 6d). To further evaluate the contribution of pretraining on large-scale epigenomic datasets to model performance, we compared the performance of SUCCEED without pretraining, which showed consistently inferior results across all metrics, highlighting the critical role of large-scale pretraining in capturing generalizable regulatory features (Supplementary Fig. 6e).

We further conducted a systematic comparison of SUCCEED with representative self-supervised DNA foundation models and supervised models on the same datasets (Supplementary Data 2). Specifically, the evaluated methods differed only in their DNA sequence encoders, while sharing an identical ATAC-seq signal encoder and joint representation decoder (“Methods”). We note that certain baseline models are subject to inherent architectural constraints, such as limitations in maximum input sequence length or original task design, which may influence performance under unified evaluation settings (“Methods”). We observed that SUCCEED and Sei, which are both pretrained on large-scale functional genomics data, consistently achieve higher performance than self-supervised sequence-only models when predicting cell-type-specific epigenomic signals (Supplementary Fig. 6f–h). This advantage remains robust across cross-chromosomal, cross-cell type, and the more stringent combined cross-chromosomal and cross-cell type evaluation settings (Supplementary Fig. 6f–h). Further analyses across multiple common epigenomic signals demonstrate that SUCCEED achieves the best overall performance (Fig. 4g). In contrast, Enformer, which is also supervised pre-trained on large-scale functional genomics data, exhibits limited performance on this task (Supplementary Fig. 6f–h). This limitation may be attributable to its training configuration, which uses 196,608 bp input sequences at 128 bp resolution and does not readily scale to the 1,048,576 bp input length and 1024 bp resolution required in this study. Moreover, Enformer was originally designed for non-coding variant effect analysis and employs a large convolutional channel capacity, leading to substantially increased computational costs when processing longer input sequences.

SUCCEED was pre-trained and fine-tuned exclusively on large-scale human functional genomics data. To assess whether the learned prior knowledge generalizes across species, we performed zero-shot transfer evaluations in multiple mouse tissues, including heart, liver, kidney, and lung (“Methods” and Supplementary Data 5). Despite having no exposure to mouse DNA sequences or functional genomics data, SUCCEED achieved high accuracy across several epigenomic signal prediction tasks, particularly for CTCF and H3K27ac (Supplementary Fig. 7). These results indicate that key aspects of genomic regulatory grammar are functionally conserved between human and mouse. Overall, these results demonstrate that SUCCEED effectively leverages regulatory grammar priors learned from large-scale external datasets to accurately predict cell-type-specific epigenomic profiles and to generalize across species in a zero-shot manner. This capability highlights its potential utility for studying transcriptional regulation in rare cell types and model organisms.

### Denoising and enhancing chromatin accessibility data with SUCCEED

**Fig. 5:**
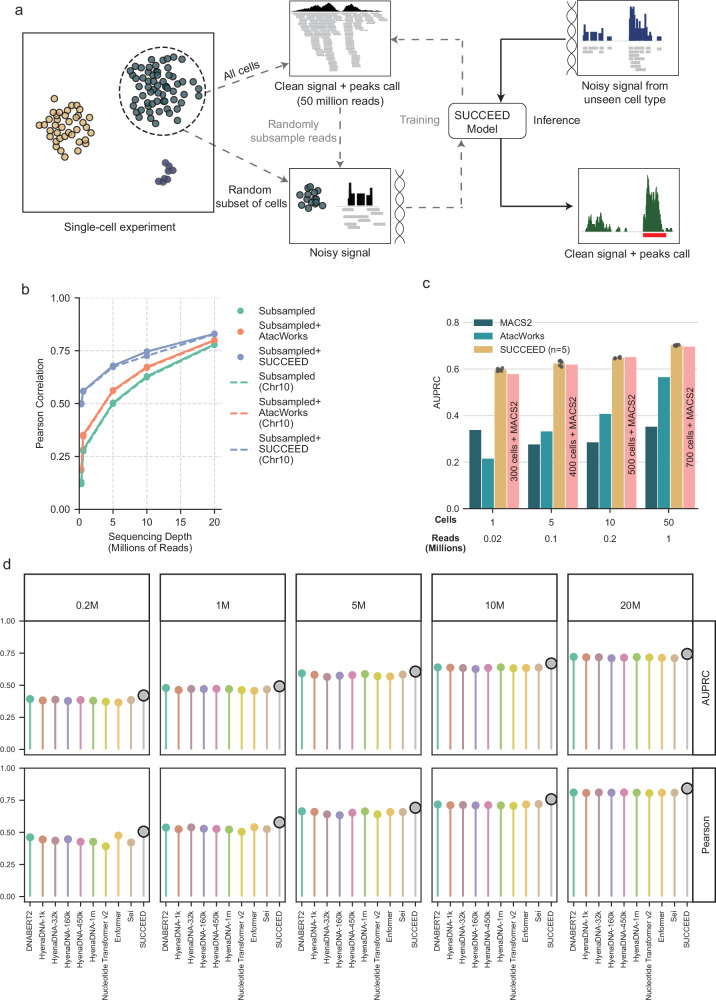
SUCCEED enhances chromatin accessibility data denoising and augmentation, outperforming other models. **a** The SUCCEED framework for chromatin accessibility profiles denoising and enhancement. The model takes 131,072 bp DNA sequences as input, combined with low-quality or noisy chromatin accessibility data. The DNA sequence is encoded through the frozen parameters of SUCCEED, while the chromatin accessibility data is processed by a Conv1D-only encoder. The two representations are concatenated and input into a dense layer to generate clean chromatin accessibility data. **b** Comparison of SUCCEED and AtacWorks on various levels of subsampling (simulating noise and low-quality), evaluated using the Pearson correlation coefficient. The solid line indicates whole-genome denoising and enhancement performance, while the dashed line represents performance on chromosome 10. **c** Performance comparison of SUCCEED, AtacWorks, and MACS2 (applied to the original input) for peak calling on scATAC-seq data at different cell numbers (1, 5, 10, and 50 cells). Bars represent the mean across five independent training runs (*n* = 5) with different random seeds; points show results from individual runs. **d** Bulk ATAC-seq denoising and enhancement. Comparison of area under the precision–recall curve (AUPRC) and Pearson correlation coefficients between predicted and experimentally measured signals across different noise levels.

ATAC-seq enables genome-wide profiling of chromatin accessibility, but its sensitivity to regulatory regions depends heavily on sequencing depth and signal-to-noise ratio^4,45^. Accurate detection of accessibility differences requires high-quality signals and sufficient coverage. However, technical factors such as sample quality, nuclear extraction, and transposase efficiency introduce systematic biases that compromise measurement accuracy. These challenges are further exacerbated in single-cell ATAC-seq (scATAC-seq), where tissue heterogeneity and sparse signals from rare cell types increase noise^46^. Given SUCCEED’s strong ability to capture the regulatory grammar of the genome, we next applied the SUCCEED framework to low-quality (sc)ATAC-seq data to enhance data quality. Based on the pre-trained SUCCEED (131,072–128 bp) model, we incorporated an encoder for low-quality or low-coverage signals together with a decoder for joint representation learning. Specifically, the pre-trained SUCCEED model is first used to extract sequence representations that capture prior knowledge, while a signal encoder learns feature representations from noisy signals. The two representations are then concatenated and fed into the decoder for joint modeling. The model takes DNA sequences and their corresponding regional noisy signals as input and outputs denoised or enhanced high-quality signals (Fig. 5a; “Methods”). We benchmarked SUCCEED against the state-of-the-art AtacWorks method, which employs a ResNet architecture to denoise accessibility data but does not incorporate DNA sequence or prior regulatory information^47^.

We first generated low-quality bulk ATAC-seq data by subsampling at different levels (“Methods”). On the test datasets, SUCCEED consistently outperformed AtacWorks in both PCC and the area under the precision-recall curve (AUPRC), for genome-wide analyses as well as for chromosome 10, which was excluded from training (Fig. 5b and Supplementary Fig. 8a). This improvement was even more pronounced within peak regions (Supplementary Fig. 8b). Notably, SUCCEED maintained high accuracy under extreme subsampling (0.2 million reads), high PCCs and AUPRC (Fig. 5b and Supplementary Fig. 8a). Using low-cell-number ATAC-seq data from early human embryonic stages (2-cell, 8-cell, ICM, hESC)^48^, SUCCEED robustly enhanced chromatin accessibility profiles (Supplementary Fig. 8d). For example, SUCCEED accurately reconstructed chromatin accessibility profile surrounding *OCT4*, a key TF during early embryo development (Fig. 5d)^49^.

We next applied SUCCEED to scATAC-seq data. Using SnapATAC2^50^, we processed raw fragment files from B cells and monocytes, randomly selecting 2400 cells to construct reference datasets and derive peak annotations (“Methods”). Sparse inputs were generated by downsampling to 1, 5, 10, or 50 cells per sample. Separate models were trained for each downsampling level and evaluated on NK cells. SUCCEED accurately reconstructed accessibility profiles even from single-cell input, achieving performance comparable to conventional methods using ~300 cells (Fig. 5c). Performance improved with cell number, and SUCCEED consistently outperformed AtacWorks across all subsampling levels. Finally, in effector CD4⁺ T cells, SUCCEED successfully recovered regulatory activity from only 50 cells with weak signal (Supplementary Fig. 8c), accurately enhancing low-quality input and identifying key regulatory elements in marker gene regions (Fig. 5e).

To further evaluate the critical role of the prior information introduced by SUCCEED in denoising and signal enhancement, we trained control models that excluded SUCCEED on the same datasets. For example, in previously unseen erythroid cell samples at a sequencing depth of 0.2 million reads, the SUCCEED-based model achieved an AUPRC of 0.38 in the peak calling task, whereas the model trained without SUCCEED reached only 0.17 (Supplementary Fig. 9a). When the evaluation was restricted to a single chromosome (chromosome 10), a consistent performance gap was observed, with AUPRC values of 0.37 and 0.18, respectively (Supplementary Fig. 9a). Further comparison of the Pearson correlation between the enhanced signals and high-quality reference data revealed the same trend at both the genome-wide level and on chromosome 10 (Supplementary Fig. 9b). In scATAC-seq signal enhancement tasks under low-cell-number conditions, SUCCEED-based models consistently outperformed models trained without SUCCEED across both NK and PBMC CD4+ cell types (Supplementary Fig. 9c, d).

We further performed a systematic comparison of SUCCEED with other self-supervised DNA foundation models and supervised models on the same datasets (“Methods” and Supplementary Data 2). Consistent with the cell-type-specific epigenomic profile prediction task, the evaluated methods differed only in their DNA sequence encoders, while sharing the same noise signal encoder and joint representation decoder. The results show that SUCCEED achieves superior overall performance in chromatin accessibility denoising and enhancement, with robust gains observed for both bulk and scATAC-seq data (Supplementary Fig. 10). Notably, Enformer also exhibits strong performance in this task, likely because its architectural design effectively accommodates the input sequence length required here and matches the target signal resolution (Supplementary Fig. 10).

To evaluate the zero-shot cross-species transferability of SUCCEED in denoising and signal enhancement, we applied the model to ATAC-seq data from early mouse embryonic development. Despite the absence of mouse data during training, SUCCEED effectively denoised and enhanced chromatin accessibility signals (Supplementary Fig. 11a). Notably, even under low-coverage conditions, the enhanced signals remained highly correlated with the original sequencing data (Supplementary Fig. 11b). Collectively, these results demonstrate that SUCCEED leverages regulatory grammar priors to robustly denoise and enhance chromatin accessibility across bulk, single-cell, and low-coverage ATAC-seq data, while generalizing across species, thereby providing a unified and scalable framework for high-resolution epigenomic profiling.

### Accurate prediction of cell-type-specific 3D chromatin architecture using SUCCEED

The 3D chromatin architecture of the same genomic locus can vary substantially across cell types and disease states, influencing gene regulation, cell identity, and replication timing^5,51,52^. High-resolution genome-wide conformation capture techniques, such as Hi-C and HiChIP, have enabled mapping of cell-type-specific regulatory interactions^53,54^. However, their high cost and technical demands limit large-scale applications, particularly for systematic studies of 3D genome organization in gene regulation^11,13,14^. To address this, Tan et al. proposed C. Origami^11^, a deep learning framework that predicts 3D chromatin conformation from DNA sequence and cell-type-specific ATAC-seq and CTCF ChIP-seq data. The model adopts an encoder-decoder architecture comprising two independent encoders, a central Transformer, and a task-specific decoder.

**Fig. 6:**
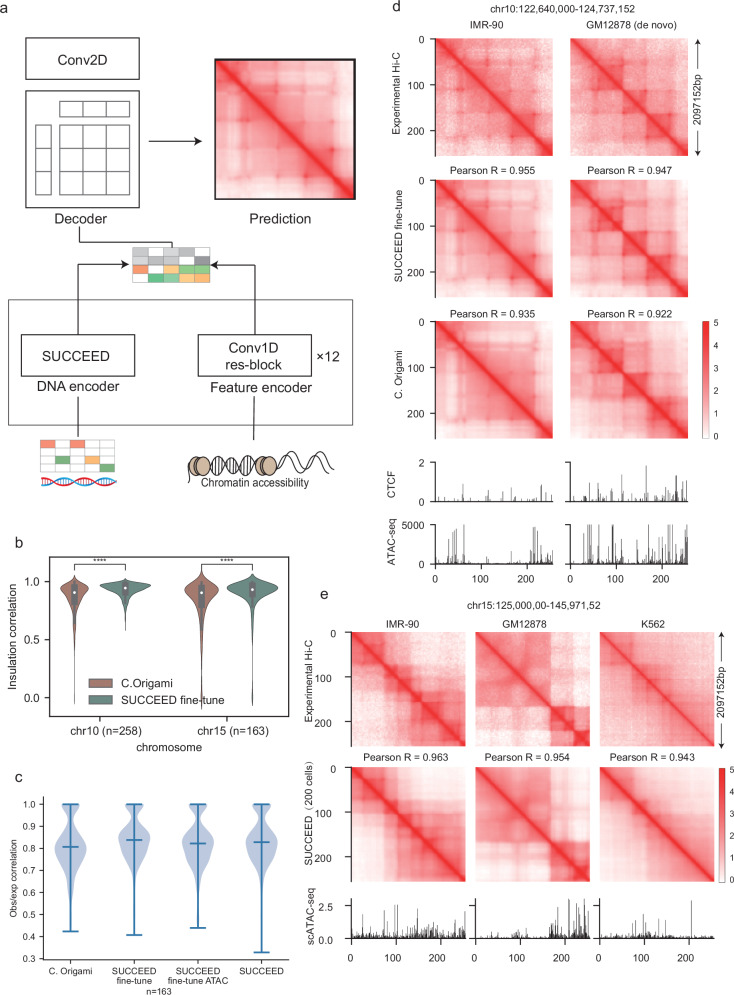
SUCCEED outperforms other methods in predicting cell-type-specific chromatin 3D structures. **a** The SUCCEED framework for predicting cell-type-specific chromatin 3D structures. The model takes as input 2,097,152 bp DNA sequences with a resolution of 8192 bp, chromatin accessibility, and CTCF ChIP-seq data. DNA sequences are encoded by SUCCEED, while chromatin accessibility data are processed by C. Origami’s feature encoder to generate compressed representations. These two representations are concatenated and passed through a decoder to predict chromatin 3D structures. **b** Performance evaluation of SUCCEED and C. Origami on the IMR-90 test and validation sets. Violin plots show the distribution of Pearson correlation coefficients between predicted and reference insulation scores, computed per genomic window on chromosome 10 and chromosome 15. The center line indicates the median and the violin width reflects the kernel density estimate. *n* denotes the number of genomic windows (chromosome 10, *n* = 258; chromosome 15, *n* = 163). Statistical significance between methods was assessed using a two-sided Mann–Whitney *U* test across genomic windows (****, *P* < 0.0001). The corresponding *P* values were 8.46 × 10^−57^ for chromosome 10 and 1.37 × 10^−22^ for chromosome 15. **c** Performan**c**e comparison of different training strategies on the IMR-90 test set. SUCCEED fine-tune denotes training in which SUCCEED parameters are unfrozen and updated during training. SUCCEED fine-tune ATAC refers to a model trained using only ATAC-seq and DNA sequences as input, with SUCCEED parameters fine-tuned during training. SUCCEED denotes training in which SUCCEED parameters are frozen and only the Feature Encoder and Decoder are optimized. Violin plots show the distribution of observed/expected correlation values across samples for each method. The width of each violin represents the kernel density estimate of the distribution. The horizontal line indicates the mean, and the vertical lines extend to the minimum and maximum values. *n* denotes the number of samples contributing to each distribution (*n* = 163 samples). **d** Experimental Hi-C data, SUCCEED fine-tune predicted Hi-C, C. Origami predicted Hi-C, CTCF ChIP-seq, and ATAC-seq signals on chromosome 10 in IMR-90 and GM12878. **e** Experim**e**ntal Hi-C data, SUCCEED fine-tune predicted Hi-C using scATAC-seq and DNA sequences (200 cells) as input, and scATAC-seq signals on chromosome 15 in IMR-90, GM12878, and K562.

However, de novo training of the dual-encoder model in C. Origami is computationally expensive and relies on both ATAC-seq and CTCF ChIP-seq data, the latter of which remain unavailable for most tissues and cell types. By contrast, SUCCEED leverages large-scale epigenomic pretraining, which enables it to learn long-range dependencies among sequence motifs (Fig. 6a). Leveraging this capability, we applied SUCCEED to predict cell-type-specific 3D chromatin architecture. Using data from the IMR-90 cell line, chromosome 10 was used as the validation set, chromosome 15 as the test set, and the remaining chromosomes for training. We benchmarked C. Origami against three SUCCEED training strategies: freezing pretrained parameters (SUCCEED), unfreezing pretrained parameters (SUCCEED fine-tune), and using only ATAC-seq data (SUCCEED fine-tune ATAC-seq). Across all strategies, SUCCEED converged more rapidly and exhibited improved training stability (Supplementary Fig. 12a).

On both validation and test sets, SUCCEED produced contact matrices that closely matched experimental data based on insulation scores (Fig. 6b and Supplementary Fig. 12b). Notably, it accurately reconstructed 3D chromatin architecture even without CTCF ChIP-seq input (Fig. 6c and Supplementary Fig. 12c). To assess cross-cell-type generalization, we trained models on multiple datasets and evaluated performance on unseen cell types. SUCCEED trained on the IMR-90 cell line performed well on the GM12878 cell line, and identified specific interactions in GM12878 cell line (Fig. 6d and Supplementary Fig. 12d). These results show that integrating pretrained SUCCEED substantially improves both predictive accuracy and training efficiency in modeling cell-type-specific 3D genome organization.

The advent of single-cell Hi-C has provided a powerful means for characterizing 3D chromatin architecture and its regulatory mechanisms across distinct cell types^55,56^. However, the technique remains experimentally challenging and yields highly sparse contact matrices, limiting its scalability for broader applications. Given the broad availability of scATAC-seq data, we tested SUCCEED’s utility for predicting cell-type-specific 3D chromatin structure^57,58^. After establishing that SUCCEED could accurately reconstruct 3D chromatin architecture using only bulk ATAC-seq data as input, we further tested whether this capability could be extended to pseudo-bulk ATAC-seq profiles generated from scATAC-seq data. We trained models using Hi-C data and pseudo-bulk ATAC-seq profiles generated from scATAC-seq data for the IMR-90 and GM12878 cell lines, and  evaluated these models on the K562 cell line. Even when trained on ≤200 cells, SUCCEED maintained high accuracy, with only modest declines in generalization (Supplementary Fig. 12e, f). It effectively captured accessibility patterns from sparse input and reconstructed high-fidelity 3D features (Fig. 6e). These findings indicate that SUCCEED enables accurate prediction of 3D chromatin architecture from small-scale scATAC-seq data, offering a scalable solution for large-scale 3D genome modeling.

We also compared SUCCEED with other self-supervised DNA foundation models and supervised models (“Methods” and Supplementary Data 2). Specifically, all methods used DNA foundation models to extract sequence representations and differed only in their DNA sequence encoders, while sharing the same ATAC-seq and CTCF ChIP-seq signal encoders and a joint representation decoder. The results indicate that SUCCEED and Sei achieve broadly comparable performance in predicting cell-type-specific 3D chromatin architecture, with their relative performance varying by data modality. When bulk ATAC-seq is used as input, SUCCEED attains slightly higher accuracy. In contrast, under single-cell ATAC-seq input, Sei—pretrained on large-scale functional genomics data—shows comparable or marginally superior performance (Supplementary Figs. 13 and 14). Results are reported for both chromosome 10 and chromosome 15; however, because chromosome 10 was used during validation, the evaluation on chromosome 15 provides a less biased assessment of generalization performance. In contrast, Enformer performs less well, and the remaining self-supervised models show similar performance to Enformer but are consistently inferior to SUCCEED and Sei (Supplementary Figs. 13 and 14).

We further evaluated SUCCEED’s ability to predict 3D chromatin architecture across species in a zero-shot setting (Supplementary Fig. 15). Specifically, we applied SUCCEED, trained exclusively on human data, to mouse early embryonic stages (8-cell and mESC) and the mouse cell line Patski (Supplementary Data 5). The results show that, using only ATAC-seq signals together with learned sequence representations, the predicted Hi-C maps exhibit high concordance with experimentally measured data in terms of insulation scores, suggesting a degree of structural conservation in topologically associating domain (TAD) formation between human and mouse (Supplementary Fig. 15a). In contrast, the correlation between predicted and experimental Hi-C data at the observed/expected level is lower than that achieved on human datasets, indicating the presence of species-specific differences in local 3D chromatin architecture. We performed a visual comparison between the predicted Hi-C maps and experimentally measured Hi-C data on mouse chromosome 3. The results show that, under a zero-shot setting, the model can accurately reconstruct three-dimensional chromatin organization across different mouse cell types and tissues using only ATAC-seq signals, demonstrating robust cross-species generalization (Supplementary Fig. 15b). These results indicate that representations learned through supervised training on large-scale epigenomic datasets are better suited for modeling and predicting cell-type-specific three-dimensional chromatin structure.

## Discussion

In this study, we introduce SUCCEED, a data-driven pretrained foundation model for genomics. The model adopts an optimized hybrid architecture: a one-dimensional convolutional network efficiently identifies conserved sequence motifs associated with cis-regulatory elements such as promoters and enhancers; these local features are then passed to a Transformer module, which captures long-range dependencies by modeling interactions among distal genomic regions. This hierarchical design enables the joint encoding of local motif signals and global regulatory context, thereby providing a more accurate representation of gene regulatory logic. Unlike self-supervised foundation models trained solely on DNA sequence, SUCCEED adopts a supervised pretraining strategy inspired by prior supervised DNA models, including AlphaGenome, Borzoi, Enformer, and Sei, and is pretrained on large-scale functional genomics data. Evaluation on additional datasets demonstrated SUCCEED’s strong transferability: with only minimal parameter fine-tuning, it achieved performance comparable to or exceeding that of de novo models, while substantially reducing computational cost and improving training efficiency.

Building on these advances, we introduced a series of transfer strategies to address diverse genomic tasks. In predicting cell-type-specific epigenomic profiles, SUCCEED outperformed the state-of-the-art EPCOT model. It also demonstrated signal denoising and enhancement, accurately reconstructing high-quality chromatin accessibility signals from low-coverage bulk ATAC-seq and sparse scATAC-seq inputs, substantially surpassing AtacWorks. When applied to modeling cell-type-specific 3D chromatin architecture, SUCCEED showed marked improvements in contact matrix accuracy and maintained strong performance even without CTCF input. Notably, SUCCEED was able to reconstruct 3D chromatin structures from small-scale scATAC-seq data, enabling cell-type-specific analysis. Further comparisons indicate that SUCCEED and Sei, both pretrained in a supervised manner on large-scale functional genomics data, generally outperform self-supervised models trained solely on DNA sequence across multiple application scenarios. These observations are consistent with recent findings by Tang et al. who similarly reported advantages of supervised pretraining over self-supervised sequence-only approaches^59^. In addition, SUCCEED exhibits zero-shot cross-species transferability, enabling accurate predictions in previously unseen cell types or species, highlighting its potential utility for studying transcriptional regulation in rare cell populations and model organisms. Overall, these results demonstrate that supervised pretraining on large-scale functional genomics data endows SUCCEED with stable and consistent performance in modeling cell-type-specific regulatory features, enabling effective transfer across data modalities, task settings, and species, from epigenomic signal reconstruction to 3D chromatin architecture analysis.

Despite its broad applicability, SUCCEED has several aspects that warrant further refinement. First, SUCCEED is currently trained in a supervised manner exclusively on human functional genomics data from the ENCODE consortium. Although ENCODE provides high-quality and well-standardized datasets, its coverage does not fully capture the diversity of tissues, cell types, and biological states present in vivo. Integrating additional functional genomics resources, such as Roadmap Epigenomics, FANTOM, and emerging single-cell epigenomic datasets, represents a promising direction for further improving model generalization, particularly in disease contexts, developmental stages, and rare cell types. Second, the computational cost of SUCCEED increases substantially with input sequence length, highlighting the need for more efficient sequence representations and attention mechanisms. In our comparisons, Sei—trained in a supervised manner on large-scale functional genomics data—achieves competitive performance using a purely convolutional architecture. SUCCEED, in contrast, adopts a hybrid convolution–Transformer architecture designed to capture long-range regulatory interactions. While this design enables SUCCEED to model distal genomic dependencies, it also introduces additional computational cost compared with convolution-only models. Extending convolutional architectures with efficient mechanisms for modeling long-range dependencies may therefore further improve the balance between predictive performance and computational efficiency. In addition, the current architecture shows limited capacity to integrate information across genomic scales ranging from 128 to 2 Mb, highlighting the challenge of developing unified multi-scale models to enhance generalization. Developing models that jointly represent multiple regulatory layers—including single-nucleotide sequence features, TF binding, nucleosome occupancy, chromatin loops, TADs, and chromatin compartments—represents an important direction for future research. Such multi-scale frameworks are likely to be important for advancing sequence-based modeling of hierarchical gene regulation. In parallel, although SUCCEED demonstrates zero-shot generalization across cell types and species, its transferability could be further enhanced by incorporating few-shot or meta-learning strategies during fine-tuning, enabling efficient adaptation to rare or previously uncharacterized cell types under limited data availability.

Nevertheless, SUCCEED provides a data-driven, transferable, and scalable foundation model for genomics, enabling unified analyses across tasks, modalities, and scales. We envision that it could serve as a central framework for integrative multi-omics studies, facilitating the investigation of cell-type-specific regulatory programs and the decoding of noncoding regulatory mechanisms.

## Methods

### Data collection and processing for pretraining

We trained the model using human functional genomics data. Input DNA sequences were derived from the UCSC hg38 reference genome (https://hgdownload.soe.ucsc.edu/goldenPath/hg38/bigZips/hg38.fa.gz). To construct the training targets for SUCCEED, we collected epigenomic datasets from the ENCODE consortium, including DNase-seq, ATAC-seq, TF ChIP-seq, and histone modification ChIP-seq experiments^31^. Only samples aligned to the hg38 assembly were retained. In total, we curated 6389 human epigenomic tracks, excluding experiments annotated in the ENCODE metadata as involving drug treatments, genetic perturbations, or other non-standard experimental conditions. For experiments with multiple biological or technical replicates, replicates from the same experiment were merged prior to inclusion.

To mitigate batch effects arising from differences in experimental protocols and laboratories, all tracks were processed using the standardized ENCODE normalization and preprocessing pipelines. Specifically, for ATAC-seq tracks, we used the Signal *p*-value bigWig files provided by ENCODE. The same Signal p-value bigWig files were used for TF and histone modification ChIP-seq data, whereas DNase-seq tracks used read-depth–normalized signal bigWig files. A complete list of the pretraining targets, including ENCODE accession numbers, assay types, cell or tissue annotations, laboratory information, replicate identifiers, and associated experimental metadata, is available at https://github.com/bioczsun/SUCCEED/blob/main/data/target_6389.txt

To align DNA sequences with functional genomics signals, we adopted the data processing pipeline from the Basenji2 repository (https://github.com/calico/basenji)^39^. The reference genome was segmented according to the model’s required input length and output resolution, and for each genomic segment, the corresponding signal values were extracted from the functional genomics datasets. Specifically, with an input sequence length of 131,072 bp and an output resolution of 128 bp, each input sequence was partitioned into 1024 non-overlapping bins of 128 bp. The signal values within each bin were averaged to generate target values that matched the model’s output resolution. To mitigate the impact of extreme signal values, the resulting signal intensities were clipped to the range of 0–32.

### SUCCEED framework

The architecture of SUCCEED is similar to Enformer^37^ and Borzoi^12^, both of which combine convolutional neural networks (CNNs) and Transformer in a hybrid design, but SUCCEED is more lightweight overall. Specifically, the architecture consists of four major components: a convolutional stem for early motif extraction, a multi-stage downsampling convolutional tower, a transformer encoder for long-range dependency modeling, and a pointwise predictive head with multi-task outputs.

#### Convolutional stem

The convolutional stem is used to extract local motifs from the input DNA sequences. CNNs are highly effective at learning hierarchical feature representations from sequential data and are particularly suited for detecting local patterns, such as DNA motifs, which are the key building blocks of regulatory elements. The stem helps the model learn these motifs in the early layers, enabling the subsequent layers to focus on more complex interactions. This component is crucial for the model’s ability to identify and capture biologically meaningful sequence patterns relevant to gene regulation. The input genomic sequence is provided as a one-hot encoded tensor of shape *(B, 4, L)*. The network begins with a stem convolutional block composed of:

1. A 1D convolution (kernel size = 19, channels = 4 to dim*/2*) to capture short-range sequence patterns, analogous to detecting TF binding motifs.
2. Batch normalization to stabilize early-stage activations.
3. A residual convolutional block, where a ConvBlock with feature dimensionality *dim/2* is wrapped in a residual connection to improve optimization and preserve low-level motif features.
4. A max-pooling operation (stride = 8), providing the first stage of spatial downsampling while retaining salient motif information.

This stem produces a feature map of size (*B*, dim*/2, L/8*) and serves as the local feature extractor.

#### Hierarchical convolutional tower

To progressively aggregate information across increasing receptive fields, the model employs a multi-stage convolutional tower. The downsampling tower enables the model to reduce spatial resolution progressively, allowing it to integrate information from different genomic regions effectively. This structure aids in capturing both local motifs and broader regulatory features at different genomic resolutions. The number of stages is determined by downsample. At each stage *i*, the input and output channel sizes (cᵢ, cᵢ₊₁*) are selected from an exponentially spaced sequence interpolating from dim*/2* to dim, ensuring stable growth in representational capacity.

Each stage consists of:

1. A ConvBlock with a 5 bp kernel for mid-range pattern extraction.
2. A residual ConvBlock with a 1 × 1 convolution, enabling channel mixing and nonlinear feature refinement.
3. A max-pooling layer with stride 2, which halves the sequence length.

This design provides controlled hierarchical compression of the input, yielding a final representation of shape: (*B*, dim, *L*/(*8×2*^num_downsample^)).

#### Adaptive sequence normalization

To allow downstream transformer processing on a fixed-length sequence, the convolutional output is passed through an adaptive average pooling layer that rescales the sequence dimension to a constant size of 1024 bins. This produces a tensor of shape (*B*, dim, 1024), where each position represents a smoothed summary of a genomic region. The tensor is then transposed to (*B*, 1024, dim) to match transformer input conventions.

#### Transformer encoder

The Transformer encoder is employed to model long-range dependencies in the data, which is critical for genomic tasks where regulatory elements often interact over long genomic distances. The self-attention mechanism in the Transformer allows the model to capture global relationships and dependencies between distant regions of the genome, which are essential for understanding complex regulatory networks. The ability to model such long-range interactions is one of the key advantages of the Transformer architecture over CNNs, particularly for tasks such as enhancer-promoter interactions and higher-order chromatin structures. Long-range dependencies are modeled using a stack of transformer blocks, each composed of a multi-head self-attention module and a position-wise feed-forward network. Positional information is introduced through RoPE, which are applied within the self-attention mechanism by rotating the query and key vectors according to their absolute positions, allowing the attention operation to encode relative positional offsets without modifying the input embeddings. The transformer encoder produces a contextualized sequence representation of shape *(B*, 1024, dim*)*.

Inspired by recent advances in large language models, several architectural components of SUCCEED were further optimized to improve scalability and efficiency. RMSNorm replaces LayerNorm in the transformer blocks, yielding better training stability and reduced memory consumption. Grouped query attention is adopted in place of standard multi-head attention, enabling more query heads to share a smaller set of key/value heads and substantially improving inference efficiency. Gated activation units (SwiGLU/SiLU) are employed in the feed-forward layers to enhance representational capacity. In the convolutional tower, standard max-pooling is used instead of attention-based pooling, simplifying the architecture and providing more robust downsampling. Collectively, these enhancements—largely motivated by techniques emerging from modern LLM systems—improve the model’s computational efficiency and scalability, enabling support for longer genomic sequences and a wide range of downstream applications. The entire architecture is implemented in PyTorch using a modular and extensible codebase.

#### Target length cropping

The cropping layer removes 64 positions from each end of the sequence to avoid computing losses at distal boundary regions. As noted in the Enformer study, these positions are disadvantaged because they can only observe regulatory elements on the side facing the sequence center, while lacking access to elements beyond the sequence boundary. After cropping, the output has shape (*B*, target length, dim).

#### Pointwise feature refinement and multi-task heads

The component enhances feature representation by rearranging the input sequence, expanding the channel dimension using a 1 × 1 ConvBlock, and introducing regularization and nonlinearity through a dropout layer (*p* = 0.1) and GELU activation. A final pointwise convolution block is applied to convert the contextualized embeddings into predictive features:

1. The sequence is rearranged to (*B*, dim, target_len).
2. A 1 × 1 ConvBlock expands the channel dimension from dim to 2 × dim.
3. The tensor is rearranged back to (*B*, target_len, 2 × dim).
4. A dropout layer (*p* = 0.1) and GELU activation introduce regularization and nonlinearity.

Finally, the pointwise predictive head with multi-task outputs is designed to enable the model to perform several tasks simultaneously, such as predicting chromatin accessibility, enhancer activity, and TF binding. Multi-task learning allows the model to share information across related tasks, which improves generalization and robustness. This architecture is particularly effective for genomic data, where different tasks often share underlying regulatory principles. The model attaches multiple prediction heads, where each head consists of:

1. A linear layer mapping (2 × dim) → *Fₖ* (the output dimensionality of task *k*)
2. A Softplus activation, ensuring non-negative outputs consistent with epigenomic signal distributions.

The model returns a dictionary of predictions: (task name*: y*_*k*_∣*k∈*output_heads).

### SUCCEED pre-training

We trained and evaluated the model across all experiments, using the Poisson negative log-likelihood loss function as used in Enformer and Basenji2. During model pretraining, we followed the data partitioning strategy used in Enformer and Basenji2, in which the genome is segmented into fixed-resolution samples and split into training, validation, and test sets using an 8:1:1 ratio. Two data augmentation strategies were applied during training: reverse-complementing the input sequences and introducing a random 1–3 bp shift. Optimization was performed using the AdamW optimizer in PyTorch with a learning rate of 1 × 10^−4^, and gradients were clipped to a maximum global norm of 0.2. Early stopping was employed with validation loss as the monitoring metric and a patience of 8 epochs (Supplementary Data 3).

### Enformer benchmark

We conducted benchmarking based on the open-source code of Enformer (https://github.com/google-deepmind/deepmind-research/tree/master/enformer). All data used for model training and evaluation were obtained from publicly available repositories (https://console.cloud.google.com/storage/browser/basenji_barnyard/data). The dataset includes 34,021 training sequences, 2213 validation sequences, and 1937 test sequences from the human genome, as well as 29,295 training sequences, 2209 validation sequences, and 2017 test sequences from the mouse genome. For each human sequence, the dataset includes 2131 TF ChIP-seq tracks, 1860 histone modification ChIP-seq tracks, 684 DNase-seq or ATAC-seq tracks, and 638 CAGE signal tracks. For the mouse genome, each sample contains 308 TF ChIP-seq tracks, 750 histone modification ChIP-seq, 228 DNase-seq or ATAC-seq, and 357 CAGE tracks. Following the procedures described in the original Enformer paper, we extended the input sequence length from the default 131,072 bp to 196,608 bp using the hg38 reference genome and made corresponding adjustments to the Basenji2 dataset.

The Enformer model weights used for benchmarking were obtained from publicly available resources (https://www.kaggle.com/models/deepmind/enformer). To ensure a fair benchmark, we retrained a SUCCEED model on the dataset provided in the original Enformer paper. Model performance was evaluated on an independent test set by calculating the PCC between predicted signals and observed signals.

### Benchmark for self-supervised DNA language models

We conducted systematic benchmarking of SUCCEED against HyenaDNA, DNABERT, DNABERT2, and NucleotideTransformer on classical promoter prediction and splice-site prediction tasks. The promoter benchmarks follow the six tasks provided in GenBench—promoter prediction, no-TATA promoter prediction, TATA promoter prediction, core promoter prediction, no-TATA core promoter prediction, and TATA core promoter prediction—all formulated as binary classification^60^. In addition, we evaluated a human splice-site prediction task from GenBench, defined as a multi-task classification problem. All experiments used the training and test splits predefined in GenBench.

Baseline results for HyenaDNA^21^, DNABERT^22^, DNABERT2^25^, and Nucleotide Transformer^23^ were obtained from the GenBench paper, which reports performance on the same predefined training and test splits. We did not re-fine-tune these baseline models, as the GenBench results were obtained using the official implementations and recommended hyperparameters and therefore represent their strongest reported performance. SUCCEED was evaluated on the same GenBench test sets to ensure comparability. For SUCCEED, we fine-tuned the pre-trained model on each downstream training set. Given that self-supervised DNA foundation models are typically benchmarked on relatively short sequences, we restricted full fine-tuning to the convolutional module of SUCCEED and appended a lightweight multilayer perceptron (MLP) classifier. Binary cross-entropy (BCE) loss was used for optimization. The comparisons between SUCCEED and self-supervised language models on cross-cell-type regulatory signal prediction, ATAC-seq signal enhancement, and cell-type-specific chromatin structure prediction are detailed in the “Methods” sections corresponding to these tasks. We evaluate performance using the accuracy metric defined in GenBench for both binary and multi-class classification tasks:

1 $${Accuracy}=\frac{{TP}+{TN}}{{TP}+{TN}+{FP}+{FN}}\,$$

where TP, TN, FP, and FN denote true positives, true negatives, false positives, and false negatives, respectively.

### Model interpretability

To dissect the sequence representations learned by the SUCCEED pre-trained model for DNA-to-epigenomic signal prediction and their contributions to distinct chromatin mark outputs, we performed interpretability analyses along two complementary approaches: (i) extraction of sequence motifs associated with first-layer convolutional filters and (ii) gradient-based attribution using the Input × Gradient method to quantify nucleotide-resolution contributions.

Motifs were derived from feature maps of the first convolutional layer. For each filter, we selected positions with activations exceeding 0.8× the filter-specific maximum, extracted the corresponding windows from the original one-hot–encoded sequences, aligned the subsequences, and constructed a position weight matrix (PWM) by aggregating nucleotide frequencies across aligned instances.

To localize the most predictive sequence positions, we computed Input × Gradient attributions using the Captum library. Gradients of the target output with respect to the input sequence were backpropagated and multiplied element-wise by the one-hot input, yielding attribution scores for each nucleotide position. These scores capture the sensitivity of model predictions to local sequence perturbations while restricting contributions to the observed nucleotide channels.

### Cross-scale and dataset testing

#### Cross-scale transfer

SUCCEED was initially trained only on input sequences of 131 k–128 bp (131,072 bp), a scale sufficient for most genomic prediction tasks. However, long-range regulatory interactions in the genome can extend beyond 1 Mb, and many downstream applications rely on modeling sequences at megabase resolution. To enable SUCCEED to operate across tasks with varying genomic contexts, we generated datasets of multiple input lengths and resolutions using the preprocessing pipeline from Basenji2: 131 k–128 bp (131,072 bp), 524 k–512 bp (524,288 bp), 1M–1024 bp (1,048,576 bp), and 2M–2048 bp (2,097,152 bp).

We first trained a model from scratch for each input length and resolution, and then used the 131 k–128 bp model as a starting point for transfer learning to the remaining resolutions. Two transfer strategies were evaluated: fine-tuning only the classification head and jointly fine-tuning the Transformer layers and classification head. Hyperparameters and loss functions were kept consistent with those used during pretraining. After training, model performance was assessed on an independent held-out test set using the Pearson correlation between predictions and ground truth as the primary evaluation metric.

#### Cross-dataset transfer

The model is transferred to epigenomic profiles from previously unseen cell types or tissues. Single-cell ATAC-seq (scATAC-seq) data for multiple human brain cell types were obtained from the CATlas database (https://decoder-genetics.wustl.edu/catlasv1/catlas_humanbrain)^43^. Pseudo-bulk profiles were generated based on cell type annotations, resulting in bigWig files for 128 distinct cell types. Following the preprocessing pipeline, signal values were extracted from the corresponding bigWig files to construct model inputs at 131 k–128 bp resolution.

We trained two models: one initialized from scratch on the human brain scATAC-seq dataset, and another obtained by fully fine-tuning the 131 k–128 bp model pretrained on the broader ENCODE dataset. Both models were evaluated on the same independent test set using identical hyperparameter configurations, and performance was quantified using the Pearson correlation between predicted and observed signals.

We evaluated the predictive performance of the model for chromatin accessibility along two dimensions: cell type and genomic region. Specifically, for each cell type and each test genomic interval of 131,072 bp, we aligned the predicted chromatin accessibility signal with the corresponding observed signal at matched genomic positions and computed the PCC between them. Performance summarized by cell type was obtained by aggregating correlation coefficients across all test intervals for a given cell type, thereby quantifying overall predictive concordance within that cell type. Conversely, performance summarized by region was obtained by aggregating correlation coefficients across cell types for a given genomic interval, reflecting variation in predictive difficulty and robustness across different genomic regions.

### Cell-type-specific epigenomic profiles prediction

#### Dataset processing

We used the same ENCODE dataset as EPCOT^44^. Four cell lines (K562, MCF-7, GM12878, and HepG2) were used for model training, and two additional cell lines (IMR-90 and A549) served as independent cross-cell-type test sets. Two feature construction strategies were employed: first, we selected epigenomic signals present across all four training cell lines, yielding 46 features; second, following the EPCOT protocol, we included signals detected in at least two cell lines, resulting in 208 features.

Following the Basenji2 workflow, we partitioned the genome into consecutive 1,048,576 bp regions (contigs) by adjusting the input length and binning window, and extracted the corresponding epigenomic tracks for each contig. This procedure produced target matrices of shape (*N, L, C*), where *N* is the number of contigs, *L* the number of bins, and *C* the number of epigenomic features.

For the ATAC-seq inputs, we obtained aligned BAM files for each cell type from ENCODE. We then generated bigWig tracks using bamCoverage with RPGC normalization, and extracted the ATAC-seq signal for each contig to construct the model inputs. The labels used during the model training process are the bigWig files of Signal P-values after standardized processing provided by ENCODE.

#### Model training

We retrained a 1M–1024 bp SUCCEED pre-trained model. To ensure strict comparability with EPCOT, we excluded all cell-type-specific epigenomic signals used in downstream prediction tasks from the pre-training stage. After pre-training, we added a lightweight encoder to capture cell-type-specific chromatin accessibility and kept all pre-trained parameters frozen during downstream training, updating only the accessibility encoder and the final classification head.

The model takes 1,048,576 bp genomic sequences together with matched chromatin accessibility profiles as input. DNA sequences are encoded by the frozen SUCCEED model, whereas cell-type-specific accessibility signals are processed by an independent encoder. The resulting representations are concatenated and passed to a classification head to predict 46 histone modification and transcription-factor-binding features. Training was performed with a batch size of 32 using the Adam optimizer and a learning rate of 0.001. Following the EPCOT training strategy, we also constructed a model predicting 208 epigenomic features, which shared the same architecture and hyperparameters, differing only in the dimensionality of the output layer. To quantify the contribution of pre-training, we performed an ablation experiment by training a model without SUCCEED pre-training, replacing the pre-trained sequence encoder with a randomly initialized one while keeping all other configurations unchanged. All models were optimized using the Poisson negative log-likelihood loss (Supplementary Data 3).

#### Model performance evaluation

The EPCOT model weights used for benchmarking were obtained from the official repository (https://github.com/liu-bioinfo-lab/EPCOT), and the input datasets were downloaded from the sources referenced therein (Supplementary Data 4). All models were evaluated on an identical held-out test set, with chromosome 2 designated for validation, chromosomes 10 and 21 for testing, and all remaining chromosomes for training. Cross-chromosomal evaluations were performed on chromosomes 10 and 20 in the K562, GM12878, HepG2, and MCF-7 cell lines, and cross-cell-type evaluations were conducted in IMR-90, A549, and H1. Model performance was quantified using the PCC between predicted and observed signals.

#### Benchmarking performance against other DNA foundation models

Pretrained weights for Enformer, DNABERT2, HyenaDNA, and Nucleotide Transformer were obtained from the Hugging Face repository, whereas pretrained Sei models were downloaded from Zenodo (Supplementary Data 1). All models were evaluated using their publicly released pretrained parameters. For SUCCEED, the pre-generated 1,048,576 bp DNA sequences were directly provided as input to extract sequence representations. For Enformer, owing to its input length constraint, each 1,048,576 bp DNA sequence was partitioned into eight subsequences of 131,072 bp, representations were extracted independently, and every eight consecutive 128 bp intervals were subsequently aggregated into a single 1024 bp bin representation. For the remaining models, each 1,048,576 bp DNA sequence was divided into 1024 non-overlapping segments of 1024 bp, which were separately processed to obtain per-bin representations. All models shared the same ATAC-seq signal encoder as SUCCEED. Decoding was performed using the prediction heads employed during pretraining, applied to the joint representations. A separate prediction head was used for each omic signal, resulting in a total of 46 heads corresponding to 46 cell-type-specific epigenomic profiles.

### Denoising and enhancing ATAC-seq data

#### Dataset processing

For the denoising and enhancement of bulk ATAC-seq data, we followed the data processing protocol established by AtacWorks and utilized its publicly available dataset. Specifically, we selected 50 million reads from each of four human immune cell types (B cells, natural killer cells, CD4⁺ T cells, and CD8⁺ T cells) to construct high-quality reference datasets. Peaks were called at full sequencing depth using MACS2^61^ to generate precise chromatin accessibility profiles. To simulate noise arising from varying sequencing depths, the reference data were downsampled to between 200,000 and 20 million reads, thereby creating low-coverage datasets. The data from the erythroblasts cell line and early human embryonic development also followed the aforementioned processing method but were not involved in the model training process.

For the scATAC-seq enhancement task, we utilized B-cell and monocyte datasets provided by AtacWorks. Using SnapATAC2^50^, we aggregated 2400 cells from each cell type (corresponding to approximately 50 million reads) to generate high-quality ATAC-seq signal tracks and peak annotations. To construct low-quality samples, random subsets of 1, 5, 10, or 50 cells were drawn from each population, thereby simulating low-coverage data. Model training was performed in a supervised manner using paired clean and noisy data. To assess generalization, natural killer (NK) cells, which were unseen during training, were used as an independent test set.

#### Model training

We employed a pretrained 131 kb–128 bp sequence encoder to extract DNA sequence representations, keeping its parameters frozen during downstream training. An independent encoder was used to extract features from low-coverage or low-quality noisy signals. The representations from both encoders were concatenated and passed through a linear layer for denoising, enhancement, and peak calling. To evaluate the effects of large-scale supervised pretraining, we performed an ablation study by training a sequence-processing model without pretraining as a control. During training, we used chromosomes 10 and 20 for testing and validation, respectively, with the remaining chromosomes allocated for training. The model was trained using MSE and BCE loss functions, weighted equally, with a batch size of 32, AdamW optimizer, and a learning rate of 0.001. Early stopping with a patience of 20 epochs was applied. AtacWorks model weights were obtained from the official repository (https://github.com/NVIDIA-Genomics-Research/AtacWorks). This model predicts based on the coverage of each genomic base, disregarding DNA sequence information, and utilizes a ResNet-like architecture. The training loss function combined MSE, 1-Pearson correlation, and BCE (Supplementary Data 3).

#### Model performance evaluation

We evaluated the performance of AtacWorks on cell lines unseen during training, assessing denoising, enhancement, and peak calling of bulk ATAC-seq data. Evaluations were conducted on the whole genome and on chromosome 10, which was not included in the training process. The evaluation metrics followed those used in the original AtacWorks paper: PCC for denoising and enhancement tasks, and AUPRC for peak calling. For human early embryonic development data, we used the SUCCEED model to generate denoised and enhanced data from low-coverage input, and measured the correlation between the original and processed data using the PCC.

#### Benchmarking performance against other DNA foundation models

Pretrained weights for Enformer, DNABERT2, HyenaDNA, and Nucleotide Transformer were obtained from the Hugging Face repository, whereas pretrained Sei models were downloaded from Zenodo (Supplementary Data 1). All models were evaluated using their publicly released pretrained parameters. For SUCCEED, pre-generated 131,072 bp DNA sequences were directly provided as input to extract sequence representations. For Enformer, owing to its architectural characteristics, sequences of the same length (131,072 bp) were used as input, and representations were extracted from the Transformer layer outputs. For the remaining models, each 131,072 bp DNA sequence was partitioned into 1024 segments of 128 bp, which were independently processed to obtain per-bin representations. All models shared the same ATAC-seq signal encoder as SUCCEED, and a single-layer MLP was used as the decoder for chromatin accessibility denoising and enhancement.

### Predicting cell-type-specific 3D chromatin structure

#### Data and processing

The datasets used for benchmarking against C. Origami^11^ were obtained from the publicly available resources released in its original publication and include Hi-C, ATAC-seq, and CTCF ChIP-seq data for the IMR-90 and GM12878 cell lines. For cross-species evaluation and zero-shot transfer experiments, Hi-C data were used after ICE normalization without additional preprocessing. For the ATAC-seq inputs, counts per million (CPM)-normalized signals were generated using bamCoverage^62^, which helps mitigate the effects of sequencing depth variability and batch effects. Hi-C contact maps for four cell lines (IMR-90, K562, GM12878, and HCT116) were obtained from GEO and 4DN databases. We downloaded mcool files aligned to the hg38 reference genome at 10-kb resolution and processed the Hi-C data using only the reversible natural logarithm transformation. CTCF ChIP-seq and ATAC-seq data were sourced from the ENCODE database; hg38-aligned BAM files were downloaded and normalized using CPM. scATAC-seq data were also acquired from ENCODE, and downsampling was performed with SnapATAC2 to generate subsets with varying cell numbers. The training data structure followed that of C. Origami and consisted of DNA sequence, CTCF binding signal, ATAC-seq signal, and Hi-C matrices. Chromosome 10 was reserved for validation, chromosome 15 for testing, and all remaining chromosomes for training. Model inputs comprised 2,097,152 bp DNA sequences and the corresponding two types of signals, and the output corresponding to the Hi-C matrix for the corresponding genomic region.

#### Model training and performance evaluation

For model training, we used a batch size of 8 and the Adam optimizer with a learning rate of 0.002. A cosine learning rate scheduler with a period of 200 epochs was employed to stabilize training. Three data augmentation strategies, consistent with those in C. Origami, were applied: (1) a 2-Mb window was selected and randomly shifted within a 0.36-Mb range; (2) the DNA sequence was reverse complemented, and the target Hi-C matrix was flipped with a probability of 0.5; (3) Gaussian noise with mean zero and standard deviation 0.1 was added to all input signals. Both C. Origami and SUCCEED models were trained on the same dataset. C. Origami consists of an encoder and a decoder; in SUCCEED, the DNA sequence encoder was replaced with the 2M–8192 bp pre-trained model. Multiple versions of SUCCEED were trained, including a variant with all parameters frozen, a fine-tuned version, and SUCCEED fine-tune ATAC, which uses only DNA sequence and ATAC-seq signal as input. All four models were evaluated on the same test set. Cell-type-specific Hi-C prediction from scATAC-seq followed a similar procedure: DNA sequence and scATAC-seq subsets with varying cell numbers were used as input, and the model output the corresponding Hi-C matrix. We trained the model using Hi-C data from IMR-90 cells, following the training protocol described in C. Origami. Chromosome 10 was used as the validation set, chromosome 15 as the test set, and the remaining chromosomes were used for training. We systematically evaluated the performance of four models on chromosome 10 and chromosome 15 in the IMR-90 cell line, as well as at the whole-chromosome scale in the GM12878 cell line.

#### Benchmarking performance against other DNA foundation models

The pretrained weights for Enformer, DNABERT2, HyenaDNA, and Nucleotide Transformer were obtained from the Hugging Face repository, whereas the pretrained Sei model was downloaded from Zenodo (Supplementary Data 1). All models were evaluated using their publicly released pretrained parameters. For SUCCEED, a pre-generated 2,097,152 bp DNA sequence was provided as input, and representations were extracted from the output of length (2,097,152–8192) bp. For Enformer, the 2,097,152 bp sequence was partitioned into 16 subsequences of 131,072 bp, which were processed independently. The resulting outputs at 128 bp resolution were aggregated to 8192 bp, and representations were taken from the post-Transformer layers. For the remaining models, the 2,097,152 bp sequence was divided into 256 segments of 8192 bp, each of which was fed into the corresponding model to extract bin-level representations. We employed the same ATAC-seq and CTCF ChIP-seq encoders as C. Origami and used its provided decoder to predict cell-type-specific 3D chromatin architecture.

#### Insulation score correlation

Model performance was assessed using the correlation of insulation scores. The insulation score is defined as the ratio of the maximal average contact intensity in the flanking regions to the contact intensity at the center region. To avoid division by zero in unmappable regions, a pseudocount based on the mean contact intensity across the entire chromosome was added. For an analysis window containing *n* interactions, the insulation score is given by:

2 $$	{Insulation} =\\ 	 \frac{\max \left(\frac{1}{n}{\sum }_{n}({left}\,{intensity}),\frac{1}{n}{\sum }_{n}({Right}\,{intensity})\right)+\,{pseudocount}}{\frac{1}{n}{\sum }_{n}({Center}\,{intensity})+\,p{seudocount}}$$

The pseudocount was set as the mean contact intensity within a 2 Mb window of the respective chromosome.

#### Distance-stratified correlation calculations

Distance-stratified correlation was calculated as the PCC between the predicted offset diagonals and the corresponding ground truth values.

### Cross-species zero-shot transfer

For the task of predicting cell-type-specific epigenomic profiles, we used the mouse mm10 reference genome together with ATAC-seq data from four tissues—heart, liver, kidney, and lung (Supplementary Data 5). To meet the input requirements of SUCCEED, BAM files were converted to RPGC-normalized bigWig files using bamCoverage. Model performance was assessed by computing the Pearson correlation between genome-wide predicted epigenomic signals and the corresponding experimentally measured profiles provided by the ENCODE consortium.

For denoising and signal enhancement, data from early mouse embryonic development were obtained from GSE66390 (Supplementary Data 5). Noise was simulated by downsampling the full datasets to varying depths using samtools. The mm10 reference genome and the downsampled noisy data were used as inputs, and performance was evaluated by the Pearson correlation between the genome-wide enhanced signals and the original high-quality data.

For cell-type-specific three-dimensional chromatin structure prediction, to mitigate the effects of sequencing depth variability, we retrained SUCCEED on CPM-normalized data based on the pre-trained model. BAM files from different mouse tissues and cell lines were subsequently converted to CPM-normalized bigWig files using bamCoverage as model inputs (Supplementary Data 5). Model performance was evaluated by comparing the predicted and experimentally measured Hi-C data at the genome-wide scale using correlations of insulation scores, observed/expected (OE) values, and distance-stratified contact frequencies.

### Reporting summary

Further information on research design is available in the Nature Portfolio Reporting Summary linked to this article.

## Supplementary information

Supplementary Information

Transparent Peer Review file

Reporting Summary

Description of Additional Supplementary Files

Supplementary Data 1-5

## Source data

Source Data

## Data Availability

The training, validation, and test datasets used to evaluate Enformer were obtained from https://console.cloud.google.com/storage/browser/basenji_barnyard/data. All data used for SUCCEED training were sourced from ENCODE, from which we downloaded all human ATAC-seq, DNase-seq, and ChIP-seq datasets aligned to the hg38 reference genome, excluding those generated under drug treatment or abnormal conditions. All datasets used for predicting cell-type-specific epigenomic signals were also sourced from ENCODE, with all samples corresponding to cell types involved in downstream tasks excluded during pre-training. Data for chromatin accessibility denoising and enhancement tasks were obtained from https://atacworks-paper.s3.us-east-2.amazonaws.com. Human early embryonic development data were retrieved from the GEO dataset GSE101571, and PBMC data from GEO dataset GSE96772. Hi-C datasets for IMR-90, GM12878, and K562 used for predicting cell-type-specific 3D chromatin structures were obtained from the original C. Origami study (GSE63525 [https://www.ncbi.nlm.nih.gov/geo/query/acc.cgi?acc=GSE63525]). Hi-C data for HCT116 were obtained from the 4D Nucleome Data Portal (accession: 4DNESKLFZ31S). ATAC-seq datasets for IMR-90, GM12878, K562, and HCT-116 were obtained from the ENCODE database with the following accession numbers: ENCSR200OML (IMR-90), ENCSR095QNB (GM12878), ENCSR483RKN (K562), and ENCSR872WGW (HCT-116). Datasets used to generate cell-type-specific Hi-C data from scATAC-seq were obtained from the ENCODE database with the following accession numbers: ENCSR778RZT (IMR-90), ENCSR680NPV (GM12878), and ENCSR217VXJ (K562). Source data are provided with this paper.

## Code availability

The code for SUCCEED is available at https://github.com/bioczsun/SUCCEED.

## References

[1] Andersson, R. & Sandelin, A. Determinants of enhancer and promoter activities of regulatory elements. *Nat. Rev. Genet.* **21**, 71–87 (2020). 31605096 10.1038/s41576-019-0173-8

[2] Gaulton, K. J., Preissl, S. & Ren, B. Interpreting non-coding disease-associated human variants using single-cell epigenomics. *Nat. Rev. Genet.* **24**, 516–534 (2023). 37161089 10.1038/s41576-023-00598-6 PMC10629587

[3] Grandi, F. C., Modi, H., Kampman, L. & Corces, M. R. Chromatin accessibility profiling by ATAC-seq. *Nat. Protoc.* **17**, 1518–1552 (2022). 35478247 10.1038/s41596-022-00692-9 PMC9189070

[4] Klemm, S. L., Shipony, Z. & Greenleaf, W. J. Chromatin accessibility and the regulatory epigenome. *Nat. Rev. Genet.* **20**, 207–220 (2019). 30675018 10.1038/s41576-018-0089-8

[5] Rowley, M. J. & Corces, V. G. Organizational principles of 3D genome architecture. *Nat. Rev. Genet.* **19**, 789–800 (2018). 30367165 10.1038/s41576-018-0060-8 PMC6312108

[6] Kim, S. & Wysocka, J. Deciphering the multi-scale, quantitative cis-regulatory code. *Mol. Cell* **83**, 373–392 (2023). 36693380 10.1016/j.molcel.2022.12.032 PMC9898153

[7] Sasse, A., Chikina, M. & Mostafavi, S. Unlocking gene regulation with sequence-to-function models. *Nat. Methods* **21**, 1374–1377 (2024). 39122947 10.1038/s41592-024-02331-5

[8] Eraslan, G., Avsec, Ž, Gagneur, J. & Theis, F. J. Deep learning: new computational modelling techniques for genomics. *Nat. Rev. Genet.* **20**, 389–403 (2019). 30971806 10.1038/s41576-019-0122-6

[9] Novakovsky, G., Dexter, N., Libbrecht, M. W., Wasserman, W. W. & Mostafavi, S. Obtaining genetics insights from deep learning via explainable artificial intelligence. *Nat. Rev. Genet.* **24**, 125–137 (2023). 36192604 10.1038/s41576-022-00532-2

[10] Greener, J. G., Kandathil, S. M., Moffat, L. & Jones, D. T. A guide to machine learning for biologists. *Nat. Rev. Mol. Cell Biol.* **23**, 40–55 (2022). 34518686 10.1038/s41580-021-00407-0

[11] Tan, J. et al. Cell-type-specific prediction of 3D chromatin organization enables high-throughput in silico genetic screening. *Nat. Biotechnol.* **41**, 1140–1150 (2023). 36624151 10.1038/s41587-022-01612-8 PMC10329734

[12] Linder, J., Srivastava, D., Yuan, H., Agarwal, V. & Kelley, D. R. Predicting RNA-seq coverage from DNA sequence as a unifying model of gene regulation. *Nat. Genet*. **57**, 949–961 (2025). 10.1038/s41588-024-02053-6 PMC11985352 39779956

[13] Zhou, J. Sequence-based modeling of three-dimensional genome architecture from kilobase to chromosome scale. *Nat. Genet.* **54**, 725–734 (2022). 35551308 10.1038/s41588-022-01065-4 PMC9186125

[14] Fudenberg, G., Kelley, D. R. & Pollard, K. S. Predicting 3D genome folding from DNA sequence with Akita. *Nat. Methods* **17**, 1111–1117 (2020). 33046897 10.1038/s41592-020-0958-x PMC8211359

[15] de Almeida, B. P., Reiter, F., Pagani, M. & Stark, A. DeepSTARR predicts enhancer activity from DNA sequence and enables the de novo design of synthetic enhancers. *Nat. Genet.* **54**, 613–624 (2022). 35551305 10.1038/s41588-022-01048-5

[16] Linder, J. et al. Interpreting neural networks for biological sequences by learning stochastic masks. *Nat. Mach. Intell.* **4**, 41–54 (2022). 35966405 10.1038/s42256-021-00428-6 PMC9373874

[17] Kelley, D. R., Snoek, J. & Rinn, J. L. Basset: learning the regulatory code of the accessible genome with deep convolutional neural networks. *Genome Res.* **26**, 990–999 (2016). 27197224 10.1101/gr.200535.115 PMC4937568

[18] Avsec, Ž et al. Base-resolution models of transcription-factor binding reveal soft motif syntax. *Nat. Genet.* **53**, 354–366 (2021). 33603233 10.1038/s41588-021-00782-6 PMC8812996

[19] Chen, K. M., Wong, A. K., Troyanskaya, O. G. & Zhou, J. A sequence-based global map of regulatory activity for deciphering human genetics. *Nat. Genet.* **54**, 940–949 (2022). 35817977 10.1038/s41588-022-01102-2 PMC9279145

[20] Novakovsky, G., Fornes, O., Saraswat, M., Mostafavi, S. & Wasserman, W. W. ExplaiNN: interpretable and transparent neural networks for genomics. *Genome Biol.* **24**, 154 (2023). 37370113 10.1186/s13059-023-02985-y PMC10303849

[21] Nguyen, E. et al. Hyenadna: long-range genomic sequence modeling at single nucleotide resolution. *Adv. Neural Inf. Process. Syst.* **36**, 43177–43201 (2023).

[22] Ji, Y., Zhou, Z., Liu, H. & Davuluri, R. V. DNABERT: pre-trained bidirectional encoder representations from transformers model for DNA-language in genome. *Bioinformatics* **37**, 2112–2120 (2021). 33538820 10.1093/bioinformatics/btab083 PMC11025658

[23] Dalla-Torre, H. et al. Nucleotide transformer: building and evaluating robust foundation models for human genomics. *Nat. Methods* **22**, 287–297 (2025). 39609566 10.1038/s41592-024-02523-z PMC11810778

[24] Nguyen, E. et al. Sequence modeling and design from molecular to genome scale with Evo. *Science* **386**, eado9336 (2024). 39541441 10.1126/science.ado9336 PMC12057570

[25] Zhou, Z. et al. DNABERT-2: efficient foundation model and benchmark for multi-species genomes. In International Conference on Learning Representations (2024).

[26] Avsec, Ž. et al. Advancing regulatory variant effect prediction with AlphaGenome. *Nature* **649**, 1206–1218 (2026). 10.1038/s41586-025-10014-0 PMC12851941 41606153

[27] Grattafiori, A. et al. The Llama 3 herd of models. Preprint at 10.48550/arXiv.2407.21783 (2024).

[28] Shazeer, N. GLU variants improve Transformer. Preprint at 10.48550/arXiv.2002.05202 (2020).

[29] Zhang, B. & Sennrich, R. Root mean square layer normalization. *Adv. Neural Inf. Process. Syst.* **32** (2019).

[30] Su, J. et al. Roformer: enhanced transformer with rotary position embedding. *Neurocomputing* **568**, 127063 (2024).

[31] Consortium EP An integrated encyclopedia of DNA elements in the human genome. *Nature* **489**, 57 (2012). 22955616 10.1038/nature11247 PMC3439153

[32] Lizio, M. et al. Gateways to the FANTOM5 promoter level mammalian expression atlas. *Genome Biol.* **16**, 1–14 (2015). 25723102 10.1186/s13059-014-0560-6 PMC4310165

[33] Kelley, D. R. et al. Sequential regulatory activity prediction across chromosomes with convolutional neural networks. *Genome Res.* **28**, 739–750 (2018). 29588361 10.1101/gr.227819.117 PMC5932613

[34] Bailey, T. L., Johnson, J., Grant, C. E. & Noble, W. S. The MEME suite. *Nucleic Acids Res.* **43**, W39–W49 (2015). 25953851 10.1093/nar/gkv416 PMC4489269

[35] Rauluseviciute, I. et al. JASPAR 2024: 20th anniversary of the open-access database of transcription factor binding profiles. *Nucleic Acids Res.* **52**, D174–D182 (2024). 37962376 10.1093/nar/gkad1059 PMC10767809

[36] Shrikumar, A., Greenside, P. & Kundaje, A. Learning important features through propagating activation differences. In *Proceedings of the 34th International Conference on Machine Learning* **70**, 3145–3153 (PMLR, 2017).

[37] Avsec, Ž et al. Effective gene expression prediction from sequence by integrating long-range interactions. *Nat. Methods* **18**, 1196–1203 (2021). 34608324 10.1038/s41592-021-01252-x PMC8490152

[38] Zhou, J. & Troyanskaya, O. G. Predicting effects of noncoding variants with deep learning–based sequence model. *Nat. Methods* **12**, 931–934 (2015). 26301843 10.1038/nmeth.3547 PMC4768299

[39] Kelley, D. R. Cross-species regulatory sequence activity prediction. *PLoS Comput. Biol.* **16**, e1008050 (2020). 32687525 10.1371/journal.pcbi.1008050 PMC7392335

[40] Jindal, G. A. & Farley, E. K. Enhancer grammar in development, evolution, and disease: dependencies and interplay. *Dev. Cell* **56**, 575–587 (2021). 33689769 10.1016/j.devcel.2021.02.016 PMC8462829

[41] Schoenfelder, S. & Fraser, P. Long-range enhancer–promoter contacts in gene expression control. *Nat. Rev. Genet.* **20**, 437–455 (2019). 31086298 10.1038/s41576-019-0128-0

[42] Benegas, G., Ye, C., Albors, C., Li, J. C. & Song, Y. S. Genomic language models: opportunities and challenges. *Trends Genet*. **41**, 286–302 (2025). 10.1016/j.tig.2024.11.013 39753409

[43] Li, Y. E. et al. A comparative atlas of single-cell chromatin accessibility in the human brain. *Science* **382**, eadf7044 (2023). 37824643 10.1126/science.adf7044 PMC10852054

[44] Zhang, Z., Feng, F., Qiu, Y. & Liu, J. A generalizable framework to comprehensively predict epigenome, chromatin organization, and transcriptome. *Nucleic Acids Res.* **51**, 5931–5947 (2023). 37224527 10.1093/nar/gkad436 PMC10325920

[45] Corces, M. R. et al. An improved ATAC-seq protocol reduces background and enables interrogation of frozen tissues. *Nat. Methods* **14**, 959–962 (2017). 28846090 10.1038/nmeth.4396 PMC5623106

[46] Fang, R. et al. Comprehensive analysis of single cell ATAC-seq data with SnapATAC. *Nat. Commun.* **12**, 1337 (2021). 33637727 10.1038/s41467-021-21583-9 PMC7910485

[47] Lal, A. et al. Deep learning-based enhancement of epigenomics data with AtacWorks. *Nat. Commun.* **12**, 1507 (2021). 33686069 10.1038/s41467-021-21765-5 PMC7940635

[48] Wu, J. et al. Chromatin analysis in human early development reveals epigenetic transition during ZGA. *Nature* **557**, 256–260 (2018). 29720659 10.1038/s41586-018-0080-8

[49] Fogarty, N. M. et al. Genome editing reveals a role for OCT4 in human embryogenesis. *Nature* **550**, 67–73 (2017). 28953884 10.1038/nature24033 PMC5815497

[50] Zhang, K., Zemke, N. R., Armand, E. J. & Ren, B. A fast, scalable and versatile tool for analysis of single-cell omics data. *Nat. Methods* **21**, 217–227 (2024). 38191932 10.1038/s41592-023-02139-9 PMC10864184

[51] Tang, Z. et al. CTCF-mediated human 3D genome architecture reveals chromatin topology for transcription. *Cell* **163**, 1611–1627 (2015). 26686651 10.1016/j.cell.2015.11.024 PMC4734140

[52] Jerkovic, I. & Cavalli, G. Understanding 3D genome organization by multidisciplinary methods. *Nat. Rev. Mol. Cell Biol.* **22**, 511–528 (2021). 33953379 10.1038/s41580-021-00362-w

[53] Mumbach, M. R. et al. HiChIP: efficient and sensitive analysis of protein-directed genome architecture. *Nat. Methods* **13**, 919–922 (2016). 27643841 10.1038/nmeth.3999 PMC5501173

[54] Lieberman-Aiden, E. et al. Comprehensive mapping of long-range interactions reveals folding principles of the human genome. *Science* **326**, 289–293 (2009). 19815776 10.1126/science.1181369 PMC2858594

[55] Stevens, T. J. et al. 3D structures of individual mammalian genomes studied by single-cell Hi-C. *Nature* **544**, 59–64 (2017). 28289288 10.1038/nature21429 PMC5385134

[56] Zhang, R., Zhou, T. & Ma, J. Multiscale and integrative single-cell Hi-C analysis with Higashi. *Nat. Biotechnol.* **40**, 254–261 (2022). 34635838 10.1038/s41587-021-01034-y PMC8843812

[57] Gao, V. R. et al. ChromaFold predicts the 3D contact map from single-cell chromatin accessibility. *Nat. Commun.* **15**, 9432 (2024). 39487131 10.1038/s41467-024-53628-0 PMC11530433

[58] Schuette, G., Lao, Z. & Zhang, B. ChromoGen: diffusion model predicts single-cell chromatin conformations. *Sci. Adv.* **11**, eadr8265 (2025). 39888999 10.1126/sciadv.adr8265 PMC11784829

[59] Tang, Z., Somia, N., Yu, Y. & Koo, P. K. Evaluating the representational power of pre-trained DNA language models for regulatory genomics. *Genome Biol.* **26**, 203 (2025). 40660356 10.1186/s13059-025-03674-8 PMC12261763

[60] Liu, Z. et al. GenBench: a benchmarking suite for systematic evaluation of genomic foundation models. Preprint at 10.48550/arXiv.2406.01627 (2024).

[61] Zhang, Y. et al. Model-based analysis of ChIP-Seq (MACS). *Genome Biol.* **9**, R137 (2008). 18798982 10.1186/gb-2008-9-9-r137 PMC2592715

[62] Ramírez, F., Dündar, F., Diehl, S., Grüning, B. A. & Manke, T. deepTools: a flexible platform for exploring deep-sequencing data. *Nucleic Acids Res.* **42**, W187–W191 (2014). 24799436 10.1093/nar/gku365 PMC4086134

