# Supplementary material for: Large-scale data-driven pre-trained DNA models enhance performance across diverse genomics tasks
Source: Nat Commun. 2026 May 14;17:6442. doi: 10.1038/s41467-026-73129-6 (PMC13377173; doi:10.1038/s41467-026-73129-6)
Supplement: Supplementary file 1 — Supplementary Information [file 41467_2026_73129_MOESM1_ESM.pdf]

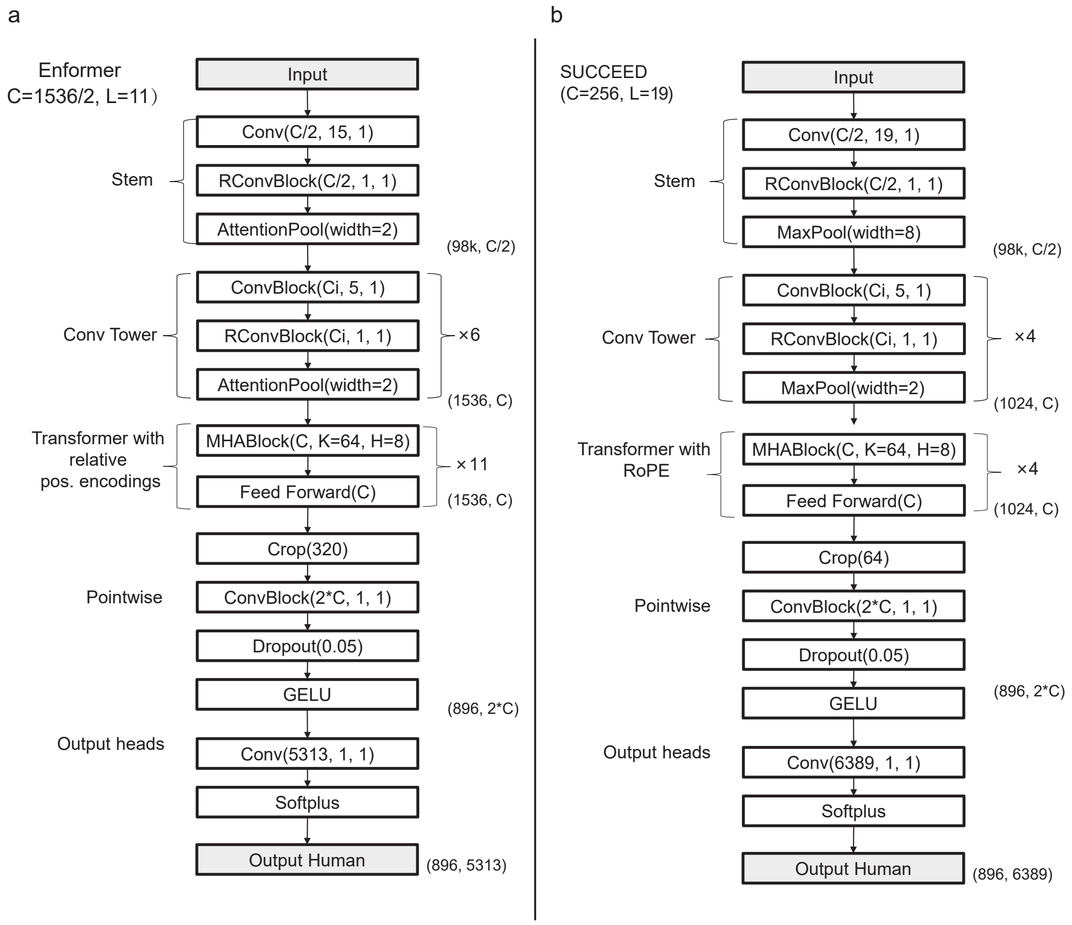

Supplementary Figure 1 | Architectures of Enformer and SUCCEED. a, The Enformer architecture, consisting of a Conv1D stem followed by a size-2 attention pooling layer, a 6-layer Conv1D convolutional tower with a size-2 attention pooling layer, 11 Transformer layers (utilizing relative position encoding as described in the Transformer-XL paper), a Crop layer to trim sequences to the required length, and an output head for 5313 human and 1689 mouse targets. **b**, The SUCCEED architecture, comprising a Conv1D stem followed by a size-8 max pooling layer, a 4-layer Conv1D convolutional tower with a size-2 max pooling layer, 11 Transformer layers (using ROPE position encoding), a Crop layer to trim sequences to the required length, and an output head for 6389 human targets.

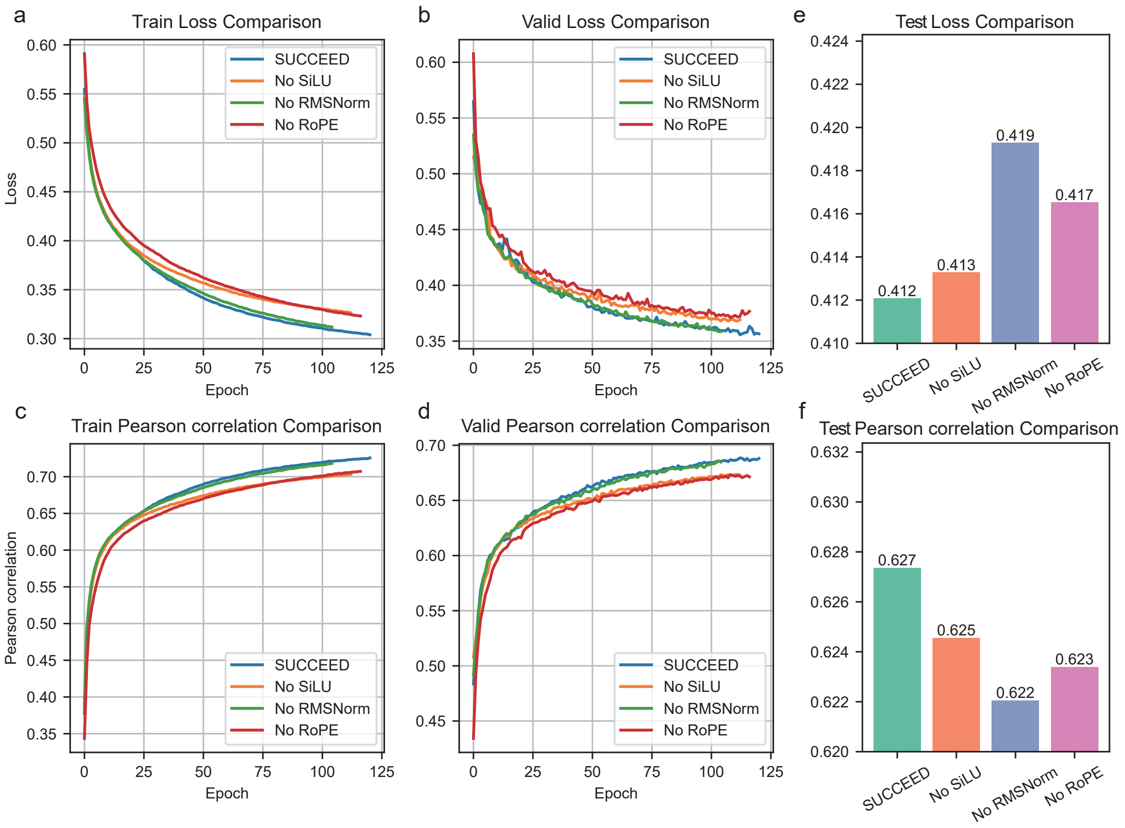

Supplementary Figure 2 | Ablation analysis of the SUCCEED framework. **a**, Poisson negative log-likelihood (NLL) loss on the training set during training for different model variants. SUCCEED denotes the full model; No SiLU replaces the SiLU activation with ReLU; No RMSNorm replaces RMSNorm with LayerNorm; No RoPE replaces rotary positional encoding with sinusoidal absolute positional encoding. **b**, Poisson NLL loss on the validation set during training for different model variants. **c**, Pearson correlation coefficient between predicted and experimentally measured signals on the training set during training for different model variants. **d**, Pearson correlation coefficient between predicted and experimentally measured signals on the validation set during training for different model variants. **e**, Poisson NLL loss between predicted and experimentally measured signals on the test set for the best-performing model of each variant. **f**, Pearson correlation coefficient between predicted and experimentally measured signals on the test set for the best-performing model of each variant.

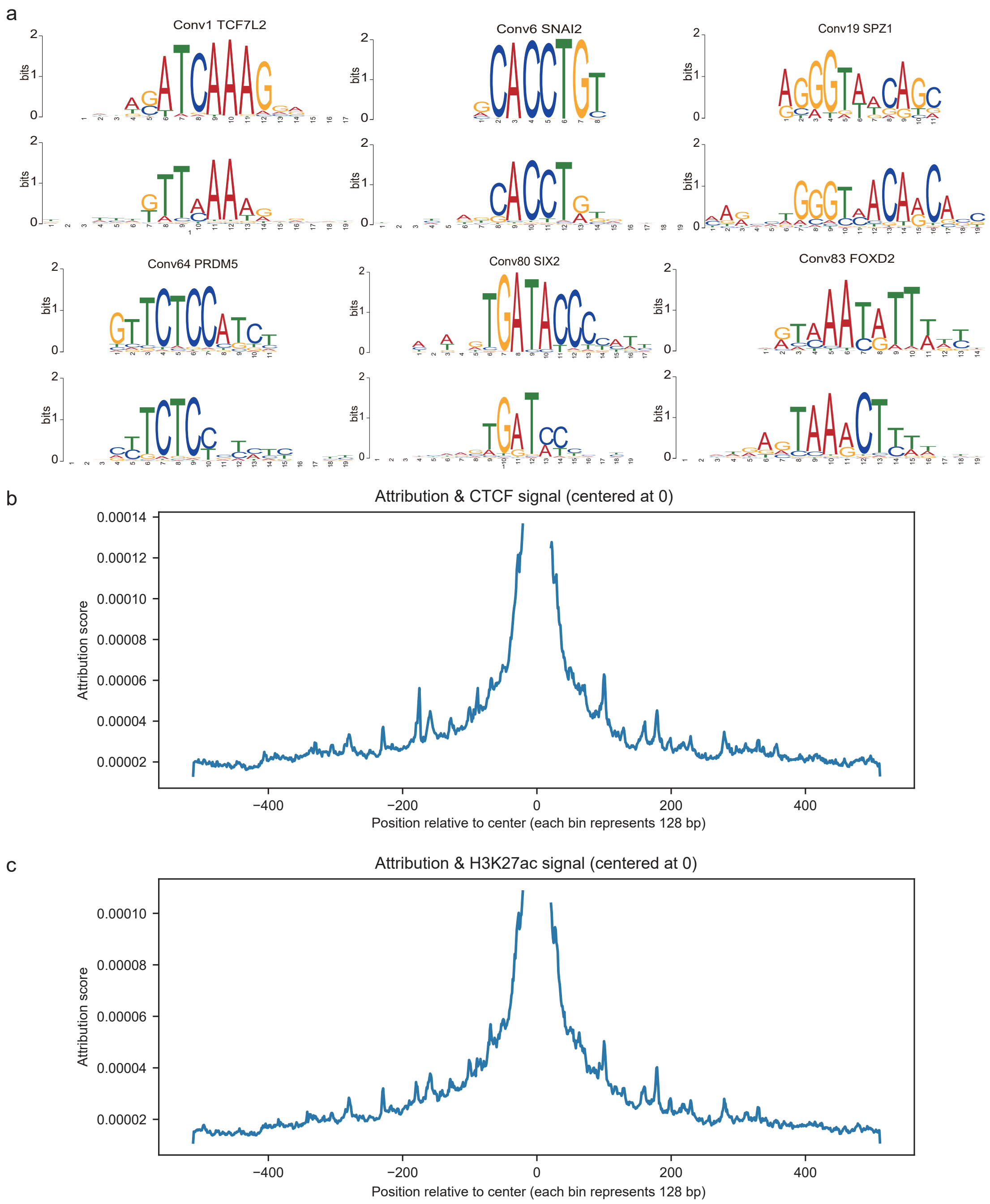

Supplementary Figure 3 | Interpretability analysis of the SUCCEED pre-trained model. a, Sequence motifs learned by the first convolutional layer of the SUCCEED model. **b**, Attribution scores computed using the Input × Gradient method for the CTCF ChIP-seq signal prediction task, aggregated across all samples; the x-axis denotes relative genomic positions. **c**, Attribution scores computed using the Input × Gradient method for the H3K27ac ChIP-seq signal prediction task, aggregated across all samples; the x-axis denotes relative genomic positions.

a

Input length	Training strategy	EPOCH	GPU usage	Batchsize	Validation R
524288	Fine-tune	17	28.28G	8	0.696
	De nove	124	28.28G	8	0.673
1048576	Fine-tune	42	25.46G	4	0.706
	De nove	91	24.98G	4	0.675
2097152	Fine-tune	40	23.94G	2	0.71
	De nove	79	23.95G	2	0.661

b

Input length	Fine-tune strategy	Total params	Trainable params	GPU usage	Batchsize
524288	Head	11474677	3277557	10.31G	8
	Transformer	11474677	10624245	14.52G	8
1048576	Head	11474677	3277557	10.21G	4
	Transformer	11474677	10624245	12.37G	4
2097152	Head	11474677	3277557	10.17G	2
	Transformer	11474677	10624245	10.29G	2

c

Methods	GPU usage	Time
Fine-tune	6G	3m
De novo	22G	11m

d

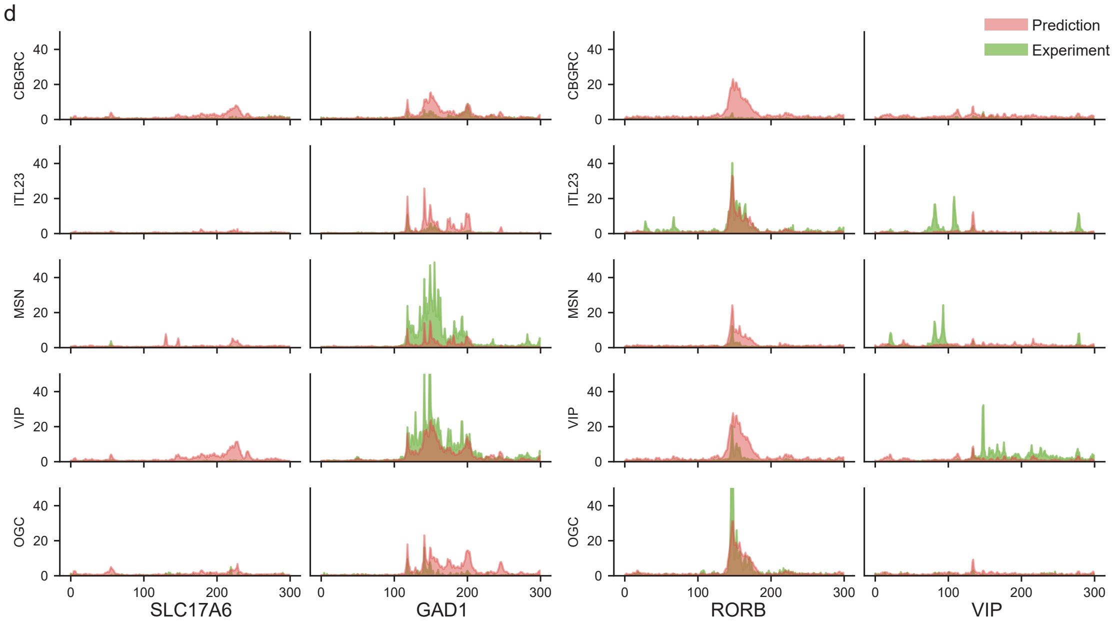

Supplementary Figure 4 | SUCCEED multi-scale de novo training and fine-tuning efficiency. a, Comparison of parameters for SUCCEED with different input sequence lengths during de novo pretraining and transfer learning. EPOCH indicates the number of training epochs required for convergence, with training stopping when the validation loss no longer decreases after 5 epochs. Validation R represents the Pearson correlation coefficient on the independent test set. **b**, Parameter comparison for fine-tuning different portions of SUCCEED with varying input sequence lengths. Head refers to fine-tuning only the final classification head, with all other parameters frozen. Transformer+Head denotes fine-tuning

both the Transformer module and the final classification head, while other parameters remain frozen. **c**, De novo training and fine-tuning efficiency on the human brain scATAC-seq dataset. Time indicates the time (in minutes) required for training each epoch with the same batch size. **d**, Representative examples of observed versus predicted genomic tracks at different gene loci in the human brain scATAC-seq test set regions.

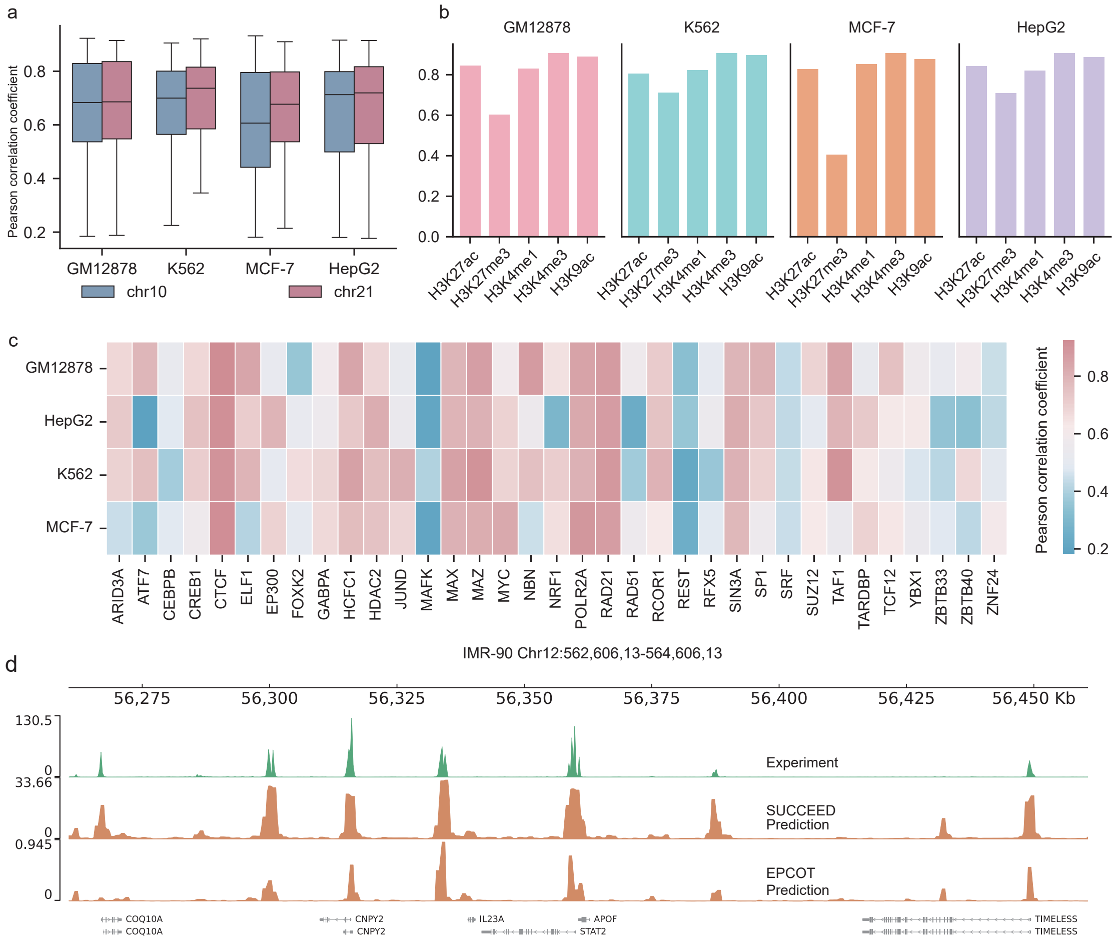

Supplementary Figure 5 | Performance comparison of SUCCEED and EPCOT. a, Performance comparison of SUCCEED on independent test and validation sets across multiple datasets, evaluated using the Pearson correlation coefficient. **b**, Performance of SUCCEED in predicting histone modification markers across multiple cell lines. **c**, Performance of SUCCEED in predicting transcription factor binding sites across multiple cell lines. **d**, Representative examples of observed versus predicted genomic tracks in the IMR-90 cell line, which the model has not encountered before.

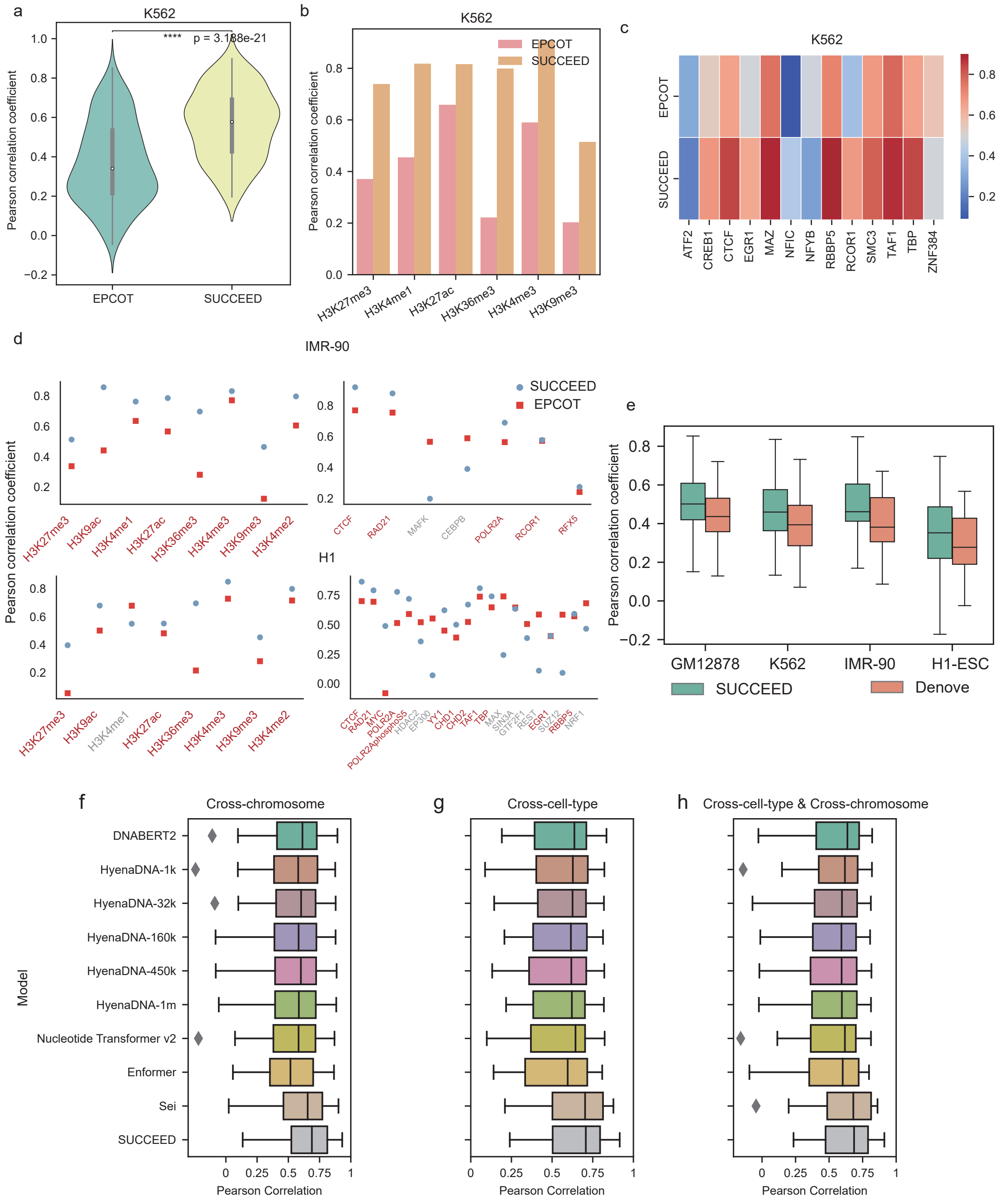

Supplementary Figure 6 | Performance comparison of DNA foundation models for predicting cell type-specific epigenomic profiles, including SUCCEED and EPCOT under different training strategies. **a**, Overall performance of SUCCEED surpassing EPCOT when trained following the EPCOT strategy (evaluated using the Pearson correlation coefficient and t-test). **b**, Performance of SUCCEED in predicting multiple histone modification markers under the EPCOT training strategy, outperforming EPCOT. **c**, Performance of SUCCEED in predicting multiple transcription factor binding sites under the EPCOT training strategy, outperforming EPCOT. **d**, Cross-cell type performance comparison of SUCCEED under the EPCOT training strategy, with red on the x-axis indicating that SUCCEED outperforms EPCOT and gray indicating the opposite. **e**, Comparison of performance between SUCCEED using a pre-

trained model (with frozen parameters) and a de novo trained sequence encoder under the EPCOT training strategy. **f**, Cross-chromosome generalization. Pearson correlation coefficients between predicted and experimentally measured epigenomic signals on held-out chromosomes (chr10 and chr21) in the training cell lines. **g**, Cross-cell type generalization. Pearson correlation coefficients between predicted and experimentally measured epigenomic signals in unseen cell lines (IMR-90 and A549) evaluated on training chromosomes (excluding chr10 and chr21). **h**, Cross-cell type and cross-chromosome generalization. Pearson correlation coefficients between predicted and experimentally measured epigenomic signals on held-out chromosomes (chr10 and chr21) in unseen cell lines (IMR-90 and A549).

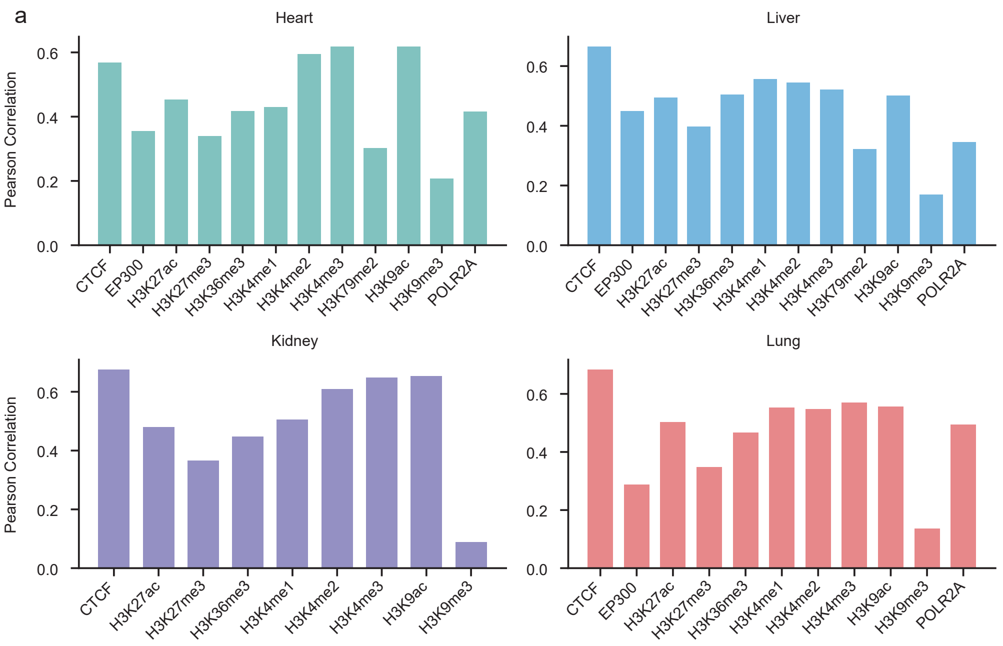

Supplementary Figure 7 | Cross-species zero-shot prediction of mouse epigenomic landscapes by SUCCEED. a, Pearson correlation coefficients between epigenomic signals predicted by SUCCEED in a zero-shot manner and experimentally measured epigenomic signals across four mouse tissues (heart, liver, kidney and lung).

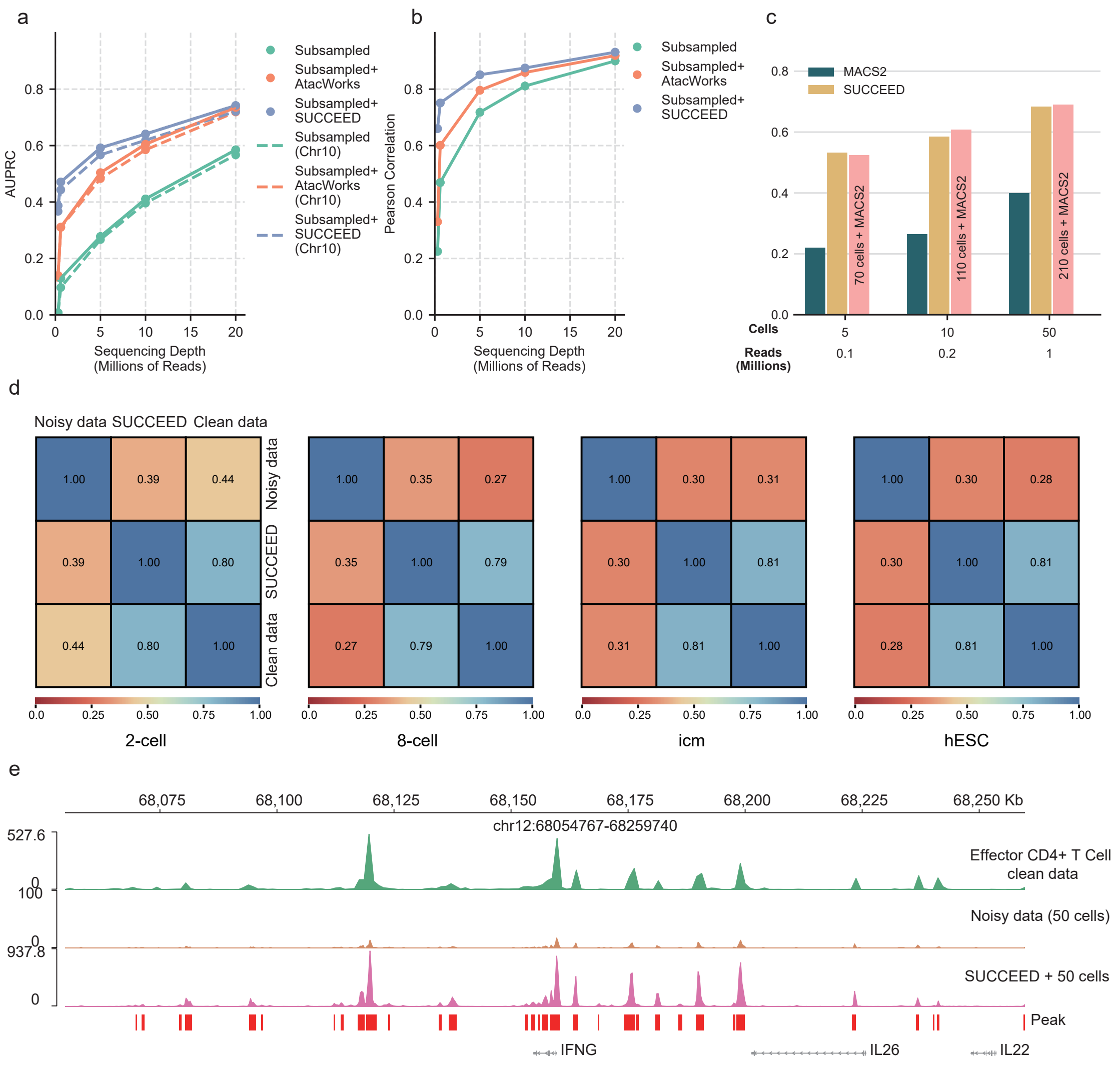

Supplementary Figure 8 | SUCCEED improves denoising of low-quality or noisy chromatin accessibility data. **a**, Performance comparison of SUCCEED, AtacWorks, and raw noisy data in calling peaks on noisy data. The solid line represents whole-genome denoising and enhancement, while the dashed line shows performance on chromosome 10. **b**, Comparison of denoising capabilities between subsampled data, AtacWorks, and SUCCEED, focusing only on peak regions. **c**, Performance of SUCCEED and subsampled data (using MACS2) in predicting peak regions on the PBMC effector CD4+ T cell dataset, evaluated using AUPRC. **d**, Denoising and enhancement performance of SUCCEED across different cell stage datasets in human early embryonic development, evaluated using the Pearson correlation coefficient. **e**, Representative tracks from raw data, noisy data, and denoised data by SUCCEED near the marker gene IFNG in scATAC-seq data from PBMC effector CD4+ T cells, a dataset unseen by the model.

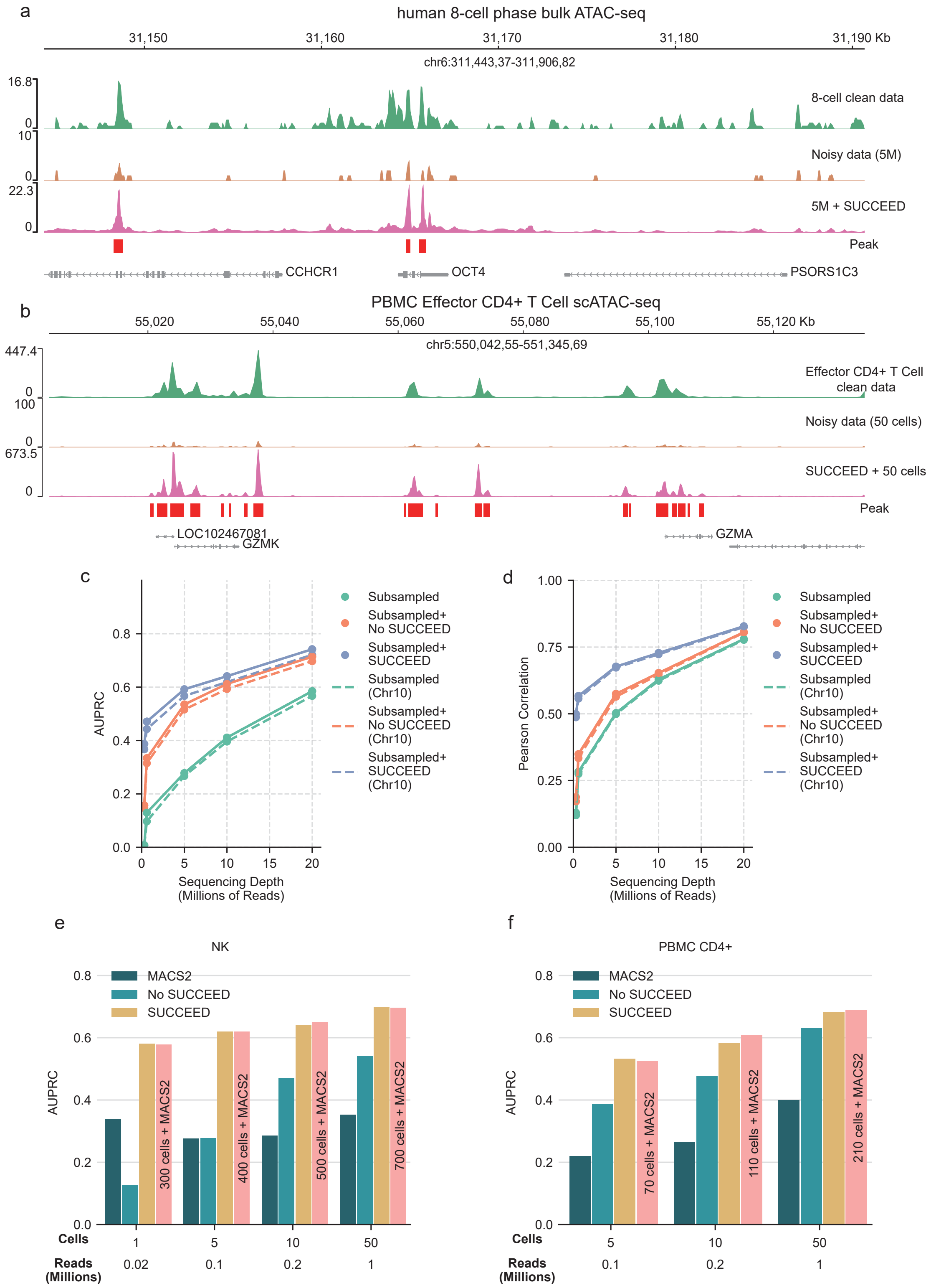

Supplementary Figure 9 | Ablation analysis of model architectures and training strategies for denoising and enhancing chromatin accessibility signals, with representative cases illustrating denoising improvement. **a**, Representative genomic tracks of raw, noisy, and SUCCEED-denoised ATAC-seq data from the 8-cell stage of human early embryonic development. These examples are

derived from a dataset that was not included in the model training. **b**, Representative tracks from raw data, noisy data, and denoised data by SUCCEED in scATAC-seq data from PBMC Effector CD4+ T cells, focusing on the marker gene *GZMK*, in a model that has not seen this data before. **c**, Area under the precision-recall curve (AUPRC) for chromatin accessibility peak denoising and enhancement in erythroid cells across different noise levels, comparing models trained with different training strategies. **d**, Pearson correlation coefficients between denoised ATAC-seq signals and experimentally measured high-quality signals in erythroid cells across different noise levels, comparing models trained with different training strategies. **e**, AUPRC for single-cell ATAC-seq peak denoising and enhancement in NK cells across varying numbers of cells, comparing models trained with different training strategies. **f**, Pearson correlation coefficients between denoised single-cell ATAC-seq signals and experimentally measured high-quality signals in PBMC CD4+ cells across varying numbers of cells, comparing models trained with different training strategies.

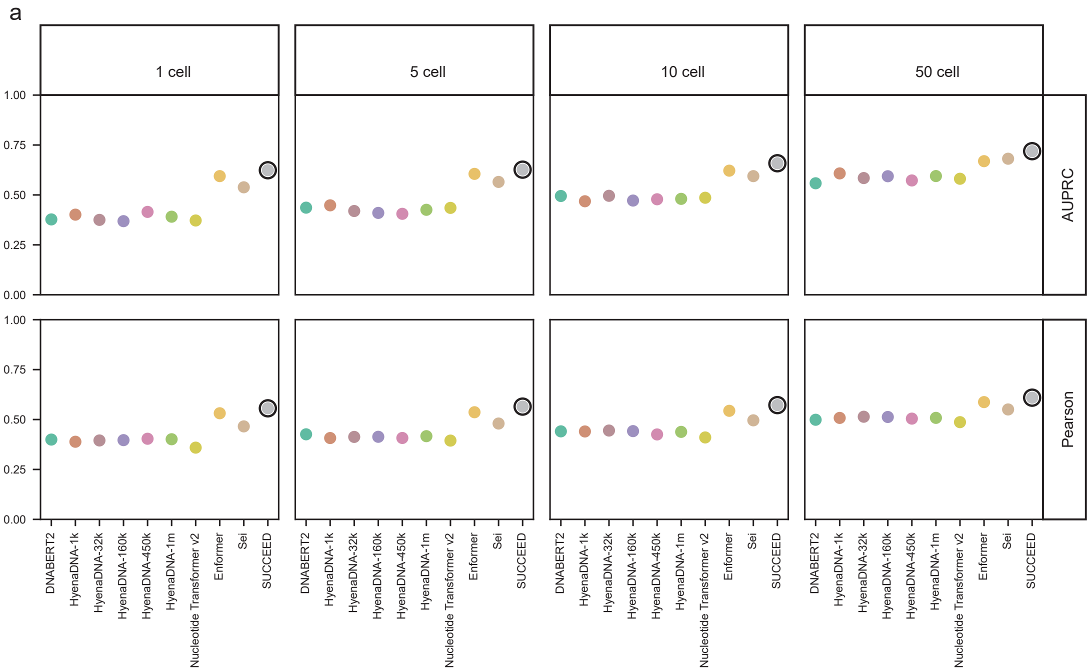

Supplementary Figure 10 | Performance comparison of DNA foundation models for denoising and enhancing chromatin accessibility data. **a**, Single-cell ATAC-seq enhancement. Comparison of AUPRC and Pearson correlation coefficients between predicted and experimentally measured chromatin accessibility signals across varying cellular coverage levels.

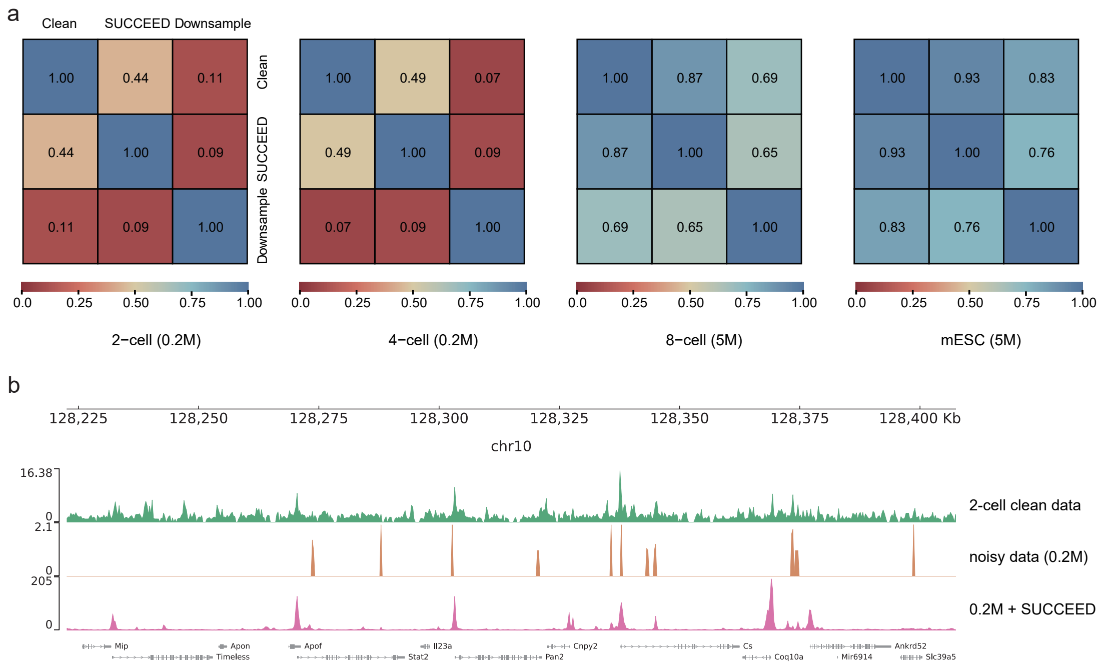

Supplementary Figure 11 | Cross-species zero-shot denoising and enhancement of ATAC-seq data by SUCCEED. a, Comparison of denoising and enhancement performance of SUCCEED during early mouse embryonic development across four stages (2-cell, 4-cell, 8-cell and mESC). Sequencing depths of 0.2M and 5M correspond to 200,000 and 5,000,000 reads, respectively. **b**, Comparison of chromatin accessibility signals after SUCCEED-based enhancement with low-coverage input signals and the original signals.

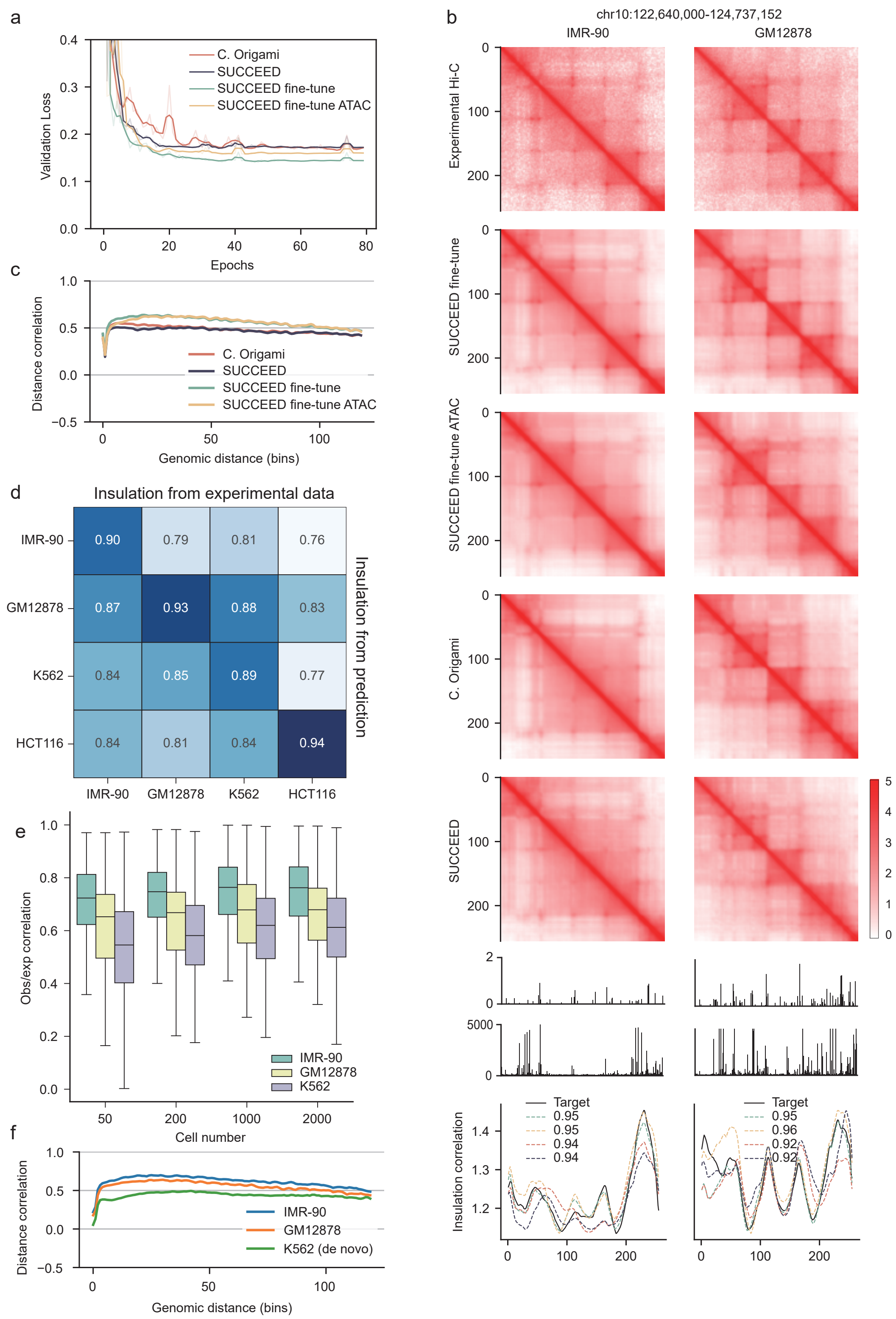

Supplementary Figure 12 | SUCCEED accurately predicts cell type-specific chromatin 3D structures. **a**, Loss comparison of four models on the validation set. The x-axis represents Epoch, and the y-axis represents the validation MSE loss. **b**, Experimental Hi-C data, SUCCEED fine-tune predicted Hi-C, SUCCEED fine-tune ATAC predicted Hi-C, C. Origami predicted Hi-C, and SUCCEED (with frozen

parameters) predicted Hi-C for IMR-90 and GM12878 on chromosome 10. The bottom panel shows the Pearson correlation coefficients of the insulation scores between the predicted Hi-C and experimental Hi-C data, from top to bottom: Target, SUCCEED fine-tune, SUCCEED fine-tune ATAC, C. Origami, and SUCCEED. **c**, Distance correlation coefficients for different models. The y-axis represents the Observed/Expected correlation coefficient, and the x-axis represents the distance in the genome. **d**, Pearson correlation between predicted and experimental Hi-C matrices calculated for different cell types using insulation scores. **e**, Observed/Expected correlation coefficients between SUCCEED fine-tune ATAC predicted Hi-C and experimental Hi-C using scATAC-seq data from different cell numbers. **f**, Distance correlation coefficients between SUCCEED predicted Hi-C and experimental Hi-C using scATAC-seq data from different cell numbers. The y-axis represents the Observed/Expected correlation coefficient, and the x-axis represents genomic distance.

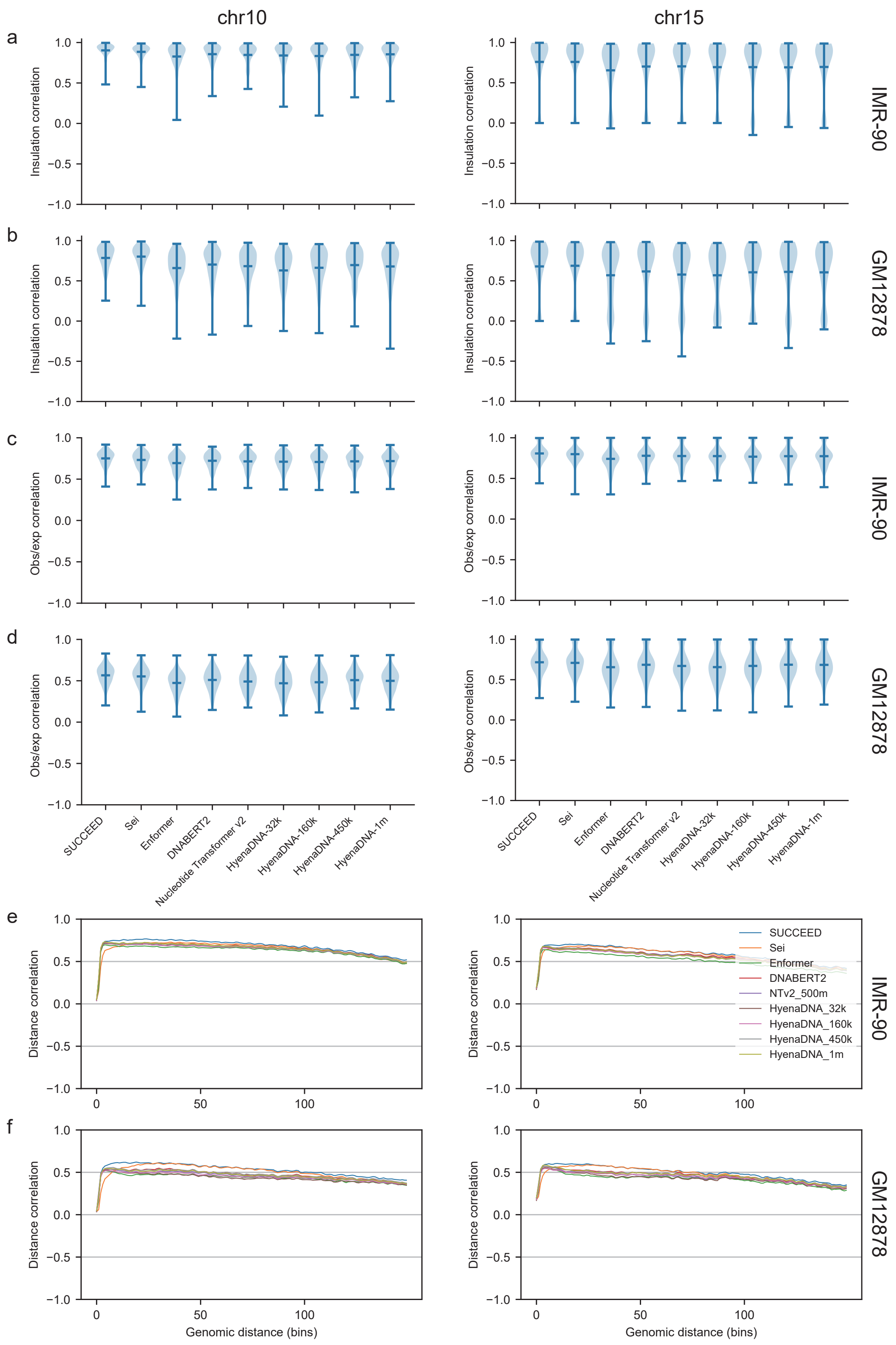

Supplementary Figure 13 | Performance comparison of DNA foundation models for predicting cell-type-specific 3D chromatin organization. **a**, Comparison of insulation score concordance between predicted and experimentally measured Hi-C contact maps on held-out chromosomes in the training cell line IMR-90. **b**, Comparison of insulation score concordance between predicted and exper-

imentally measured Hi-C contact maps on held-out chromosomes in the unseen cell line GM12878. **c**, Comparison of observed/expected (O/E) contact frequency concordance between predicted and experimentally measured Hi-C contact maps on held-out chromosomes in the training cell line IMR-90. **d**, Comparison of observed/expected (O/E) contact frequency concordance between predicted and experimentally measured Hi-C contact maps on held-out chromosomes in the unseen cell line GM12878. **e**, Comparison of distance-stratified Pearson correlation coefficients between predicted and experimentally measured Hi-C contact maps on held-out chromosomes in the training cell line IMR-90. **f**, Comparison of distance-stratified Pearson correlation coefficients between predicted and experimentally measured Hi-C contact maps on held-out chromosomes in the unseen cell line GM12878.

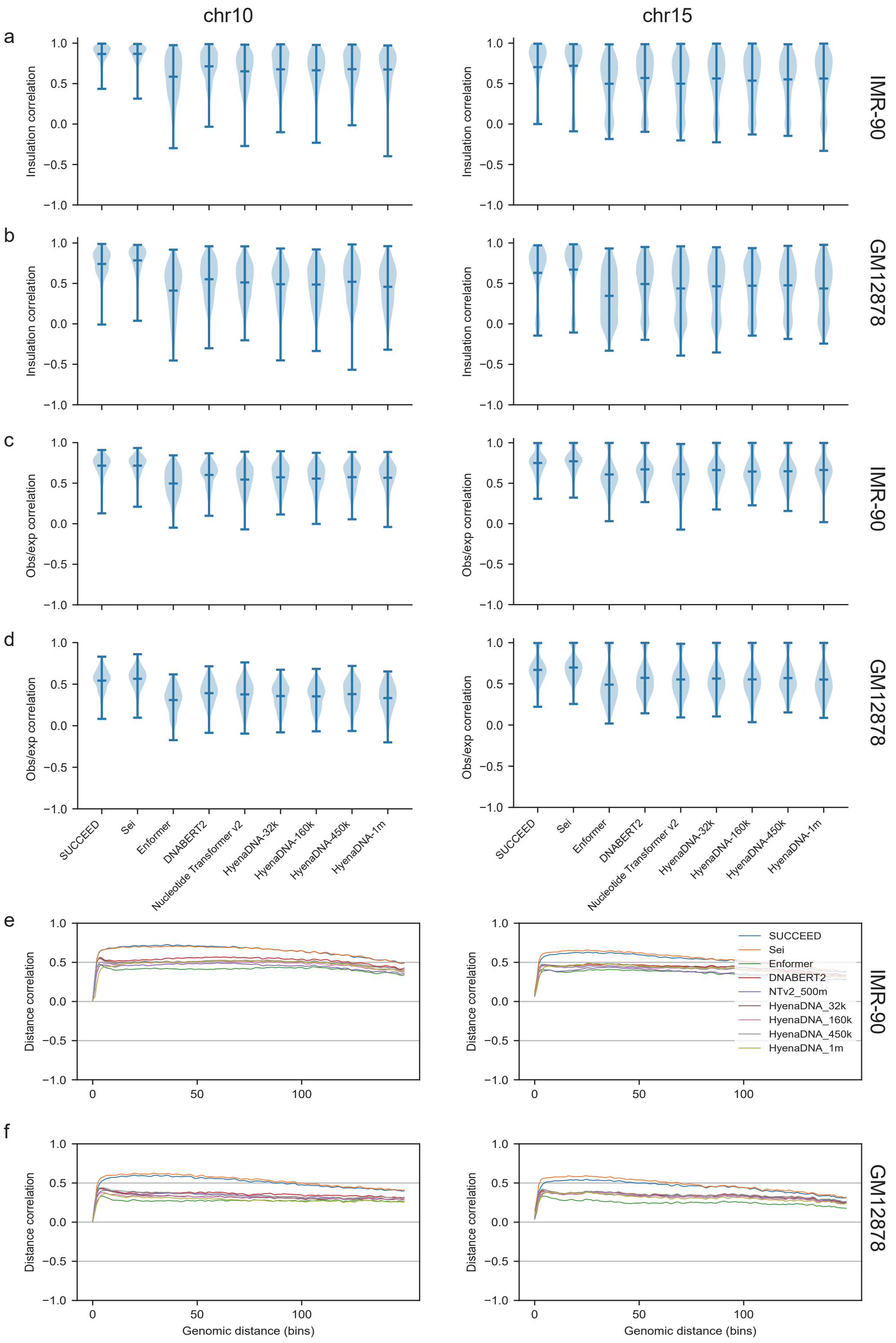

Supplementary Figure 14 | Performance comparison of DNA foundation models for predicting cell type–specific 3D chromatin organization from single-cell ATAC-seq data. **a**, Comparison of insulation score concordance between Hi-C contact maps predicted from scATAC-seq and experimentally measured Hi-C data on held-out chromosomes in the training cell line IMR-90. **b**, Comparison of

insulation score concordance between Hi-C contact maps predicted from scATAC-seq and experimentally measured Hi-C data on held-out chromosomes in the unseen cell line GM12878. **c**, Comparison of observed/expected (O/E) contact frequency concordance between Hi-C contact maps predicted from scATAC-seq and experimentally measured Hi-C data on held-out chromosomes in the training cell line IMR-90. **d**, Comparison of observed/expected (O/E) contact frequency concordance between Hi-C contact maps predicted from scATAC-seq and experimentally measured Hi-C data on held-out chromosomes in the unseen cell line GM12878. **e**, Comparison of distance-stratified Pearson correlation coefficients between Hi-C contact maps predicted from scATAC-seq and experimentally measured Hi-C data on held-out chromosomes in the training cell line IMR-90. **f**, Comparison of distance-stratified Pearson correlation coefficients between Hi-C contact maps predicted from scATAC-seq and experimentally measured Hi-C data on held-out chromosomes in the unseen cell line GM12878.

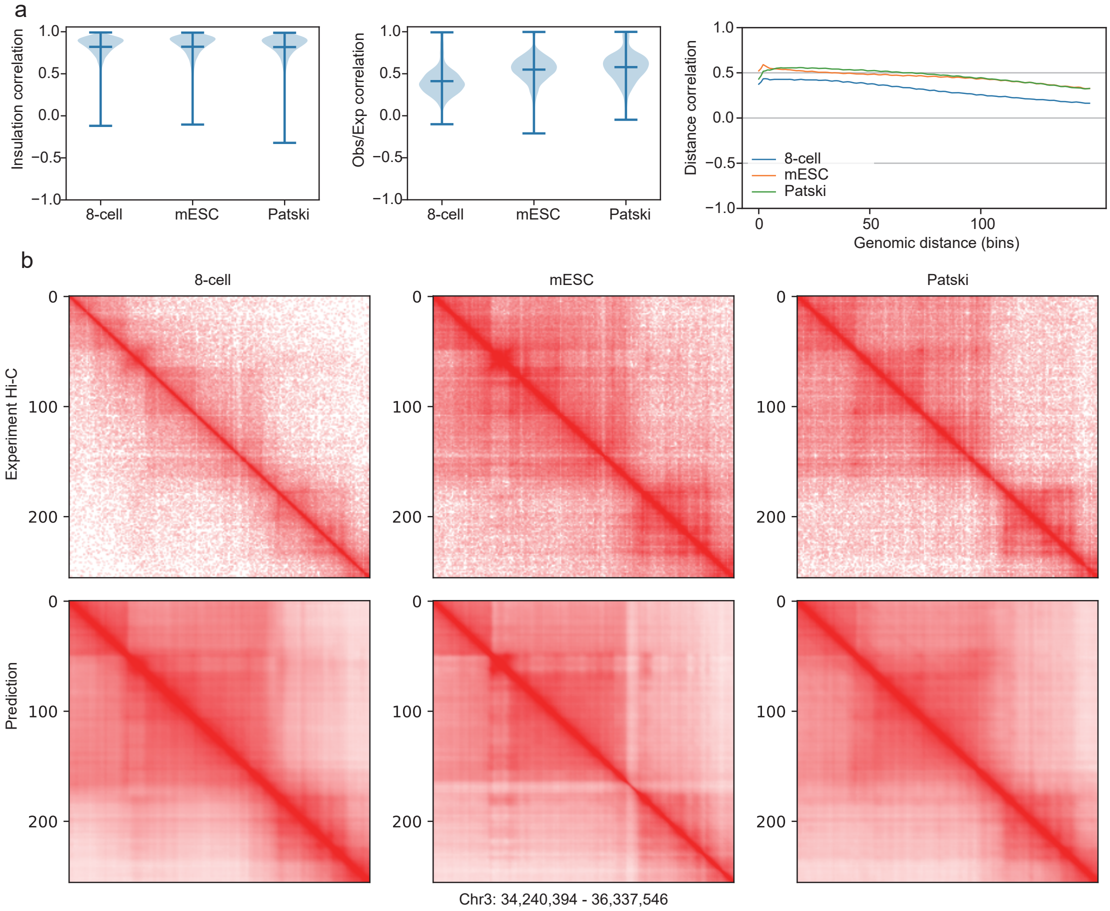

Supplementary Figure 15 | Cross-species zero-shot prediction of 3D chromatin organization in mouse tissues and cell lines by SUCCEED. a, Pearson correlation coefficients between predicted and experimentally measured Hi-C contact maps across different mouse tissues and cell lines (8-cell, mESC and Patski). From left to right, correlations are shown for insulation scores, observed/expected (O/E) contact frequencies and distance-stratified contact frequencies. Hi-C contact maps were predicted by SUCCEED from DNA sequence and ATAC-seq signals. **b**, Representative visualization of predicted and experimentally measured Hi-C contact maps on chromosome 3 (chr3) across different mouse tissues and cell lines.
