# Supplementary material for: Large-scale data-driven pre-trained DNA models enhance performance across diverse genomics tasks
Source: Nat Commun. 2026 May 14;17:6442. doi: 10.1038/s41467-026-73129-6 (PMC13377173; doi:10.1038/s41467-026-73129-6)
Supplement: Supplementary file 4 — Description of Additional Supplementary Files [file 41467_2026_73129_MOESM4_ESM.pdf]

Description of Additional Supplementary Files

Supplementary Data 1: Summary of datasets used in SUCCEED pretraining.

Supplementary Data 2: Summary of pretrained model sources across different foundation models.

Supplementary Data 3: Hyperparameters used for model training across different tasks.

Supplementary Data 4: Datasets used for benchmarking different downstream tasks.

Supplementary Data 5: Prediction of mouse cell-type-specific epigenomic landscapes, Denoising and enhancement of chromatin accessibility data during early mouse embryonic development, and Prediction of mouse cell-type-specific three-dimensional chromatin organization.
