# Supplementary material for: Large-scale data-driven pre-trained DNA models enhance performance across diverse genomics tasks
Source: Nat Commun. 2026 May 14;17:6442. doi: 10.1038/s41467-026-73129-6 (PMC13377173; doi:10.1038/s41467-026-73129-6)
Supplement: Supplementary file 2 — Transparent Peer Review file [file 41467_2026_73129_MOESM2_ESM.pdf]

nature portfolio

Peer Review File

Large-scale data-driven pre-trained DNA models enhance performance across diverse genomics tasks

Corresponding Author: Professor Hebing Chen

This file contains all reviewer reports in order by version, followed by all author rebuttals in order by version.

Version 0:

Reviewer comments:

Reviewer #1

(Remarks to the Author)
Sun et al. present SUCCEED, a new DNA foundation model trained on thousands ENCODE tracks spanning multiple tissues and cell types. The approach is trained in a supervised way on specific signals (chromatin accessibility, histone modifications, transcription factor binding) and thus belong to the category of "supervised foundation models", by opposition to the self-supervised models trained only on DNA sequences.

The authors first show that the approach has comparable accuracy as Enformer (another well known supervised foundation model) to predict signals used in the learning phase. Next, they assess its ability to transfer the learned DNA grammar to new models trained on different other tasks: 1) the prediction of new scATAC-seq signal after fine-tuning; 2) the prediction of new cell-type specific signals by integrating chromatin accessibility; 3) the denoising of low depth ATAC-seq data; 4) the prediction of 3D chromatin conformation by integrating specific ATAC-seq data.

Several dozens DNA foundation models have been proposed since 2023, using different architectures, different training strategies and different tokenisations. A majority of them are self-supervised (Nucleotide Transformer, DNABERT, HyenaDNA, ...) but other well known approaches are supervised (Enformer, Borzoi, Sei, ...). Although the performances of the approach presented here seem interesting there are in my opinion two important issues in this paper:

- First, and most importantly, it is not clear how the approach compares to all other foundation models (supervised and self-supervised) on the transfer tasks. The authors have compared the accuracy of SUCCEED and Enformer for predicting signals used for training, but how do SUCCEED compares to Enformer, DNABERT2, HyenaDNA, and Sei on the different transfer tasks? This is specifically the purpose of foundation models, so they should be compared on these tasks.

- Second, the description of methods and results are usually very short. There is no precise description of the different models, no discussion of the choices in the architectures and no assessment of these decisions. This should be done at least for the main SUCCEED model. For example, what is the benefit in term of accuracy or

training time to replace ReLU/GeLU by SwiGLU/SiLU? What gain in accuracy the rotary positional embedding has allowed? What is the effect of the replacement of the LayerNorm by RMSNorm?

The same issue also applies on experiments, where the details of the exact plans are missing. For example, what is exactly done in the comparison of Fig. 2d? What are these 7 tasks? How are conducted the fine-tunings? How are measured the accuracies?

Without these details and additional experiments, it is difficult to gauge the impact of the results or even their accuracy. For example, in the denoising task, what is exactly the gain of SUCCEED, i.e. what would be the accuracy of the same model trained using only the noisy signal (without SUCCEED)?

Additionally, it seems that there are some problems in the experiments. Specifically, in the comparisons with EPCOT, for several marks such as H3K27ac, H3K4me1, MAFK, and CEBPB, the accuracy of EPCOT is better on cross-lines (Fig. 4.e) than on the cell lines used during training (4.c & 4.d), which is quite suspect IMO.

(Remarks on code availability)

Reviewer #2

(Remarks to the Author)
This paper presents SUCCEED, a supervised multi-task DNA foundation model pre-trained on a massive scale of 6,389 ENCODE tracks. Departing from masked self-supervised learning approaches, SUCCEED employs a hybrid architecture that synergistically combines one-dimensional convolutional layers for local motif detection and Transformer layers for capturing long-range regulatory dependencies. This design enables the model to learn the complex syntax of cis-regulatory grammar directly from DNA sequence to functional outputs. Benchmarking demonstrates that SUCCEED achieves performance on par with or superior to the established Enformer model across various tasks like CAGE, TF binding, and histone modification prediction, despite being a more lightweight model. Crucially, the authors showcase SUCCEED's exceptional transferability and scalability. Through efficient fine-tuning strategies, the model generalizes robustly to unseen cell types, different sequence lengths, and resolutions. Its capabilities extend beyond standard prediction to more challenging applications: it effectively denoises and enhances both bulk and single-cell ATAC-seq data, outperforming AtacWorks; it accurately predicts cell-type-specific epigenomic landscapes (histone marks, TF binding), surpassing EPCOT; and it infers 3D chromatin architecture from DNA sequence and ATAC-seq data alone, rivaling or exceeding the performance of C. Origami without requiring CTCF ChIP-seq input. In conclusion, SUCCEED positions itself as a powerful, versatile, and scalable foundation model that unifies inference across multiple genomic tasks, modalities, and scales, leveraging large-scale supervised pre-training to capture a generalizable understanding of genomic regulatory logic.

Some detailed suggestions below aim to enhance the study’s scientific rigor. Addressing them would further solidify the conclusions and broaden the impact of this valuable research.

1. Considering the training on human data, it would be fascinating to explore the model's zero-shot or few-shot capabilities on data from model organisms. This could shed light on the conservation of the learned regulatory grammar and broaden its application in comparative genomics.

2. To further capture the hierarchical nature of genome regulation, it might be beneficial to investigate more explicit multi-scale modeling techniques within the architecture. This could potentially allow for even more seamless integration of features from nucleosome to topologically associating domain scales.

3. To empower biologists with deeper insights, integrating advanced model interpretation techniques as a standard component of the framework could help in precisely identifying causative sequence motifs and regulatory interactions underlying the predictions.

4. The model's remarkable transferability could be extended by incorporating few-shot or meta-learning paradigms into the fine-tuning process. This would be particularly valuable for predicting regulatory landscapes in rare or novel cell types where data is extremely scarce.

5. The manuscript states in both the Methods and Results sections (“We obtained 6,389

human epigenomic profiles from ENCODE, excluding those generated under drug treatment or abnormal conditions") that 6,389 tracks were used for model pretraining. However, it does not provide the detailed selection criteria. It remains unclear whether biological/technical replicates were removed, whether batch effects across laboratories were normalized, and what specific filters were applied to exclude abnormal samples. I recommend that the authors provide a complete list of all tracks in the Supplementary Materials, along with experimental metadata and the normalization pipeline.

6. In the Enformer benchmark section, the authors mention that they "retrained a SUCCEED model on the dataset provided in the original Enformer paper" and extended the input length to 196,608 bp. However, several comparisons appear to use inconsistent input lengths, resolutions, or preprocessing steps among SUCCEED, Enformer, EPCOT, and C.Origami, which may affect fairness. The authors should clearly describe, for each comparison, the training and evaluation protocol, including input length, whether model weights were frozen, batch size, optimizer, learning-rate schedule, early-stopping criteria, and random seeds. A supplementary table aligning these settings across models would substantially improve transparency and fairness.

7. The manuscript reports many averaged performance metrics (e.g., PCC and AUPRC in Figures 2–5) without specifying the number of independent runs or providing confidence intervals/standard deviations. Given that deep learning models are sensitive to random initialization and data partitioning, this omission raises concerns about robustness. The authors should repeat each key comparison (particularly those with Enformer, EPCOT, AtacWorks, and C.Origami) at least five times and report mean ± SD or bootstrap 95% confidence intervals, including full tables in the Supplementary Materials to support reproducibility.

(Remarks on code availability)
The code is running good and instruction is clear.

Reviewer #3

(Remarks to the Author)
In this manuscript, the authors present SUCCEED, a supervised multi-task DNA foundation model pretrained on 6,389 ENCODE tracks, combining a hybrid CNN-Transformer architecture to model both local sequence motifs and long-range regulatory dependencies. SUCCEED demonstrates competitive or superior performance across a range of genomic tasks, including epigenomic landscape prediction, chromatin accessibility denoising, and 3D chromatin architecture modeling. Notably, the model supports multi-resolution inputs, effective transfer learning, and generalization across unseen cell types.

Overall, this is a timely, well-executed, and impactful study. The authors convincingly argue that supervised foundation models — when trained on large-scale functional genomics datasets — can outperform self-supervised models that rely solely on masked language modeling. SUCCEED contributes a scalable and extensible framework that addresses key challenges in genome modeling, including task-specific retraining and underutilization of available epigenomic resources.

The suggestions below are intended to enhance the manuscript:

Major Comments

1. Data source diversity
The current pretraining exclusively uses data from the ENCODE database. While ENCODE offers high-quality and standardized datasets, it is not fully representative of all tissues, cell types, and biological contexts. The authors are encouraged to discuss whether incorporating additional data sources (e.g., Roadmap Epigenomics, FANTOM, or scENCODE) could further improve generalization.

2. Handling of label noise and batch effects
As the model is trained in a supervised setting, label noise and batch effects from sequencing depth, protocol variations, and experimental conditions are inevitable. The authors should explicitly describe any strategies used to mitigate these issues (e.g., quality control thresholds, normalization, data augmentation, or loss weighting). This would be especially relevant in denoising tasks and across-cell-type transfer evaluations.

3. Mechanistic architecture rationale
While the manuscript describes the use of convolutional layers for motif detection and Transformers for long-range interactions, a deeper explanation of how these components interact — including the rationale for architectural choices like rotary embeddings, grouped query attention, and their biological relevance — would strengthen the understanding of the model's design.

4. Cross-species transferability
All experiments are conducted on the human genome (hg38). Since several recent genomic foundation models demonstrate potential for cross-species generalization, it would be valuable to include experiments (or at least discussion) on

SUCCEED's performance when applied to non-human genomes (e.g., mouse, zebrafish). This would enhance the model's relevance for comparative genomics and evolutionary studies.

5. Biological interpretation of multi-resolution modeling
The model supports various input lengths and resolutions (e.g., 131k-128bp to 2M-2048bp). However, the manuscript lacks a biological interpretation of how these scales map to genomic features (e.g., nucleosomes, topologically associating domains, enhancers). A brief discussion linking resolution to biological phenomena would clarify the motivation for supporting multiple resolutions.

Minor Comments

1. Methodological details
The Methods section, while comprehensive, would benefit from clearer structure and concise descriptions of:
(1) Data partitioning strategies (e.g., how training/test chromosomes were selected)
(2) Specific hyperparameters (e.g., batch size, learning rate, optimizer)
(3) Early stopping criteria and fine-tuning protocols (especially in multi-task and cross-cell-type settings)

2. Clarification in Figure 1
In the diagram summarizing model capabilities (Fig. 1), the explanation of "unseen datasets" is somewhat ambiguous. Please clarify whether it refers to unseen cell types, data modalities, or genomic regions, and how transfer learning is achieved in each case.

3. Error bars in Figure 2b
Some histone modification bar plots in Figure 2b appear to lack error bars, while others include them. Please ensure consistency and clarify whether the error bars represent standard deviation or standard error across features/tasks.

4. Axis labels in Figure 3c and 3d
The terms "cell type" and "region" in the scatter plots require clarification. Do they refer to specific cell populations and genomic loci, respectively? Please define these labels either in the figure caption or main text for interpretability.

Conclusion

This work presents a significant step forward in the development of scalable and biologically informed genomic foundation models. With some clarifications and improved methodological transparency, the paper is well-positioned for publication in Nature Communications.

(Remarks on code availability)

Version 1:

Reviewer comments:

Reviewer #1

(Remarks to the Author)
This second version has been substantially improved. Method descriptions have been expanded and the manuscript now includes some rational about architectural choices as well as assessments of key neural-network components. Most importantly, the authors have included comparisons against several other well known foundation models, an aspect that was absent from the first manuscript.

This last point is particularly important. Dozen foundation models have been already proposed in last years, so these comparisons should be a central point for any new approach. As such, I do not think that they are sufficiently highlighted in the new manuscript and I would recommend rewriting certain parts to highlight them better:

1/ first, rather than being exclusively in supp. figures, these results should appear (at least partly) in the main figures 4, 5 & 6.

2/ I would recommend to rewrite some parts that present the performances of Succeed in a way that is a little bit biased. Notably:

- l.146-184: it is difficult to say that Succeed "outperforms" the models trained from scratch as both models achieve almost identical accuracy (I doubt the difference is even statistically significant).

- l.287-289: Succeed seems to perform slightly better solely on cross-chromosome comparisons, which are not of prime interest here. The goal of the task is to "characterize epigenomic profiles in unseen cell types" (l.223-224), which corresponds to the cross-cell-type comparison where Sei and Succeed have very similar accuracy.
- l.446-449: We cannot really say "The results show that SUCCEED achieves the best performance among all methods". Succeed seems to perform slightly better than Sei in bulk ATAC-seq (supp. fig. 13) but slightly worse in single cell (supp. fig. 14). It is also misleading to present the results achieved on chromosome 10 and 15 on the same level (see also Fig. 6). Chromosome 10 has been used for validation purpose, so the measured accuracies tend to be optimistically biased. Hence the only valid comparisons should be done on chromosome 15.

3/ I would also recommend to entirely rewrite the Discussion to discuss the performance of the other foundations models. Overall, Succeed has very similar performances as Sei, sometimes slightly better for certain tasks, and slightly worse for others.

Additionally, given their very similar performance, Succeed and Sei should also be compared in terms of model complexity (number of variables) and computation time for learning.

4/ The abstract should also be rewritten to include these comparisons and conclusions.

5/ Given the results achieved by Succeed and the other foundation models an important recent reference is missing IMO. The paper of Tang et al. 2025 (Genome Biology) reaches very similar conclusions as this paper (supervised models are better than self-supervised models). This should also be discussed in the Discussion.

(Remarks on code availability)

Reviewer #2

(Remarks to the Author)
The research method of this work is advanced, and the research content is of great significance. All the issues I am concerned about have been resolved, and I agree to accept this manuscript.

(Remarks on code availability)
The code for this work is feasible and efficient.

Reviewer #3

(Remarks to the Author)
This manuscript presents SUCCEED, a large-scale supervised multi-task DNA foundation model trained on 6,389 ENCODE tracks, integrating convolutional neural networks and Transformer architectures to jointly model local sequence motifs and long-range regulatory dependencies. Overall, the study is carefully designed, technically sound, and addresses an important problem in genomic sequence-to-function modeling.

Noteworthy contributions and significance:
The most notable contribution of this work is the demonstration that supervised pretraining on large-scale functional genomics data can yield foundation models with strong transferability across tasks, resolutions, and biological contexts, including epigenomic profile prediction, chromatin accessibility denoising, and 3D chromatin architecture inference. The comprehensive evaluation across bulk and single-cell ATAC-seq data, multiple cell types, and varying sequence lengths highlights the versatility and scalability of the proposed framework. The comparison with established methods such as Enformer, EPCOT, AtacWorks, and C. Origami is thorough and, in the revised version, appears fair and well controlled.

The work is clearly of relevance to the genomics and computational biology communities, particularly in the context of ongoing debates regarding supervised versus self-supervised foundation models for biological sequence modeling.

Soundness of methodology and evidence:
The methodology is generally sound, and the conclusions are well supported by extensive experimental evidence. The authors have substantially improved the manuscript by:

- Providing clearer architectural descriptions and motivations;
- Adding ablation studies to assess the impact of key architectural choices (e.g., SwiGLU, RMSNorm, rotary positional embeddings);
- Expanding benchmarking to include a broader set of foundation models under unified downstream decoders;
- Addressing reproducibility through repeated runs and reporting variability.

These additions significantly strengthen the technical rigor and transparency of the work.

Clarity and reproducibility:
The revised Methods section is detailed and largely sufficient to enable reproduction of the main experiments. Data preprocessing, normalization strategies, and training protocols are described clearly, and the inclusion of supplementary tables listing pretraining tracks and experimental configurations is particularly helpful.

Minor points and suggestions:
While the manuscript is already strong, a few minor clarifications could further improve clarity:
1. In the main text, it may be helpful to consistently emphasize when SUCCEED achieves comparable versus superior performance, particularly in tasks where differences with strong baselines (e.g., Sei or Enformer) are modest.
2. A brief reminder in the main text that some comparisons are constrained by inherent architectural limits of baseline models (e.g., maximum input length) could help non-specialist readers interpret the benchmarks more carefully.
3. The Discussion section appropriately outlines future directions (e.g., explicit multi-scale architectures, few-shot/meta-learning); these points could optionally be highlighted more concisely to avoid overextending the scope.

Overall assessment:
In summary, this is a high-quality and timely study that makes a meaningful contribution to the field of genomic foundation models. The revised manuscript addresses prior concerns in a thorough and convincing manner, and I believe it is suitable for publication in Nature Communications.

(Remarks on code availability)

Open Access This Peer Review File is licensed under a Creative Commons Attribution 4.0 International License, which permits use, sharing, adaptation, distribution and reproduction in any medium or format, as long as you give appropriate credit to the original author(s) and the source, provide a link to the Creative Commons license, and indicate if changes were made.
In cases where reviewers are anonymous, credit should be given to 'Anonymous Referee' and the source.
The images or other third party material in this Peer Review File are included in the article's Creative Commons license, unless indicated otherwise in a credit line to the material. If material is not included in the article's Creative Commons license and your intended use is not permitted by statutory regulation or exceeds the permitted use, you will need to obtain permission directly from the copyright holder.
To view a copy of this license, visit https://creativecommons.org/licenses/by/4.0/

Response to Reviewer #1

Reviewer #1 (Remarks to the Author):

Sun et al. present SUCCEED, a new DNA foundation model trained on thousands ENCODE tracks spanning multiple tissues and cell types. The approach is trained in a supervised way on specific signals (chromatin accessibility, histone modifications, transcription factor binding) and thus belong to the category of "supervised foundation models", by opposition to the self-supervised models trained only on DNA sequences.

The authors first show that the approach has comparable accuracy as Enformer (another well known supervised foundation model) to predict signals used in the learning phase. Next, they assess its ability to transfer the learned DNA grammar to new models trained on different other tasks: 1) the prediction of new scATAC-seq signal after fine-tuning; 2) the prediction of new cell-type specific signals byintegrating chromatin accessibility; 3) the denoising of low depth ATAC-seq data; 4) the prediction of 3D chromatin conformation by integrating specific ATAC-seq data.

Several dozens DNA foundation models have been proposed since 2023, using different architectures, different training strategies and different tokenisations. A majority of them are self-supervised (Nucleotide Transformer, DNABERT, HyenaDNA, ...) but other well known approaches are supervised (Enformer, Borzoi, Sei, ...). Although the performances of the approach presented here seem interesting there are in my opinion two important issues in this paper:

- First, and most importantly, it is not clear how the approach compares to all other foundation models (supervised and self-supervised) on the transfer tasks. The authors have compared the accuracy of SUCCEED and Enformer for predicting signals used for training, but how do SUCCEED compares to Enformer, DNABERT2, HyenaDNA, and Sei on the different transfer tasks? This is specifically the purpose of foundation models, so they should be compared on these tasks.

Response:

We sincerely appreciate the reviewer for this important and constructive comment. We acknowledge that the original manuscript did not include comparisons with other genomic foundation models on downstream transfer tasks. In the revised version, we have substantially expanded the experimental evaluation to include direct comparisons between SUCCEED and multiple representative genomic foundation models, including Enformer, DNABERT2, HyenaDNA, Nucleotide Transformer, and Sei, across a diverse set of downstream transfer tasks (Supplementary Fig. 6, Supplementary Fig. 10, Supplementary Fig. 13 and Supplementary Fig. 14).

Specifically, we assessed these models on (1) prediction of cell-type-specific epigenomic profiles, (2) denoising and enhancement of chromatin accessibility signals, and (3) prediction of cell-type-specific three-dimensional chromatin organization. For each downstream task, we first generated sequence segments in BED format as model inputs. Pretrained models for Enformer, DNABERT2, HyenaDNA, and Nucleotide Transformer were obtained from the Hugging Face repository, while pretrained Sei models were downloaded from Zenodo (Supplementary Table 2). All models were evaluated using their publicly released pretrained weights. As the majority of existing genomic foundation models are designed to operate on input sequence lengths of at most ~20 kb, we adopted a consistent bin-based representation strategy to enable fair comparison across architectures. Specifically, for long genomic regions (e.g., 1048576bp), we partitioned each sequence into 1,024 bins of 1, 024 bp and extracted representations for each bin accordingly. It is challenging to apply these DNA self-supervised foundational models on DNA sequences longer than 20k; therefore, for downstream task benchmarks, we only train task-specific decoders. For each downstream task, we ensured that all models shared the same downstream prediction architecture, and that training, validation, and evaluation were conducted on identical datasets with matched data splits and evaluation metrics. Across all evaluated downstream tasks, this controlled benchmarking consistently shows that models pretrained on large-scale functional genomics data outperform sequence-only self-supervised models. Notably, SUCCEED achieves the best overall performance across the evaluated tasks, including cell-type-specific epigenomic landscape prediction and chromatin accessibility denoising and enhancement, with performance gains remaining robust under cross-chromosomal, cross-cell-type, and combined generalization settings (Supplementary Figs. 6 and 10). In the prediction of cell-type-specific three-dimensional chromatin architecture, SUCCEED performs on par with Sei, whereas sequence-only self-supervised models and Enformer consistently show lower performance under the same evaluation protocol (Supplementary Figs. 13 and 14). Enformer's reduced performance in this setting likely reflects limitations in scaling its original architecture to substantially longer genomic contexts. Taken together, these results indicate that supervised pretraining on large-scale functional genomics data can also yield regulatory representations with strong transferability, maintaining robust performance across diverse genomic tasks and sequence scales. This observation suggests that future DNA foundation model research need not be restricted to purely self-supervised paradigms, and that supervised signals can serve as an effective complement.

The corresponding benchmarking results have been added to the revised manuscript and are presented in Supplementary Figs. 6, 10, 13 and 14.

Revised manuscript:

Page 8, Line 278: "*We further conducted a systematic comparison of SUCCEED with representative self-supervised DNA foundation models and supervised models on the same datasets (Supplementary Table 2). Specifically, the evaluated methods differed only in their DNA sequence encoders, while sharing an identical ATAC-seq signal encoder and joint representation decoder (Methods). We observed that SUCCEED and Sei, which are both pretrained on large-scale functional genomics data, consistently achieve higher performance than self-supervised sequence-only models when predicting cell-type-specific epigenomic signals (Supplementary Fig. 6). This advantage remains robust across cross-chromosomal, cross-cell type, and the more stringent combined cross-chromosomal and cross-cell type evaluation settings (Supplementary Fig. 6). Further analyses across multiple common epigenomic signals demonstrate that SUCCEED achieves the best overall performance (Supplementary Fig. 6). In contrast, Enformer, which is also supervised pre-trained on large-scale functional genomics data, exhibits limited performance on this task. This limitation may be attributable to its training configuration, which uses 196,608bp input sequences at 128bp resolution and does not readily scale to the 1,048,576bp input length and 1,024bp resolution required in this study. Moreover, Enformer was originally designed for non-coding variant effect analysis and employs a large convolutional channel capacity, leading to substantially increased computational costs when processing longer input sequences.*"

Page 11, Line 370: "*We further performed a systematic comparison of SUCCEED with other self-supervised DNA foundation models and supervised models on the same datasets (Methods and Supplementary Table 2). Consistent with the cell-type-specific epigenomic profile prediction task, the evaluated methods differed only in their DNA sequence encoders, while sharing the same noise signal encoder and joint representation decoder. The results show that SUCCEED achieves superior overall performance in chromatin accessibility denoising and enhancement, with robust gains observed for both bulk and single-cell ATAC-seq data (Supplementary Fig. 10). Notably, Enformer also exhibits strong performance in this task, likely because its architectural design effectively accommodates the input sequence length required here and matches the target signal resolution (Supplementary Fig. 10).*"

Page 13, Line 442: "*We also compared SUCCEED with other self-supervised DNA foundation models and supervised models (Methods and Supplementary Table 2). Specifically, all methods used DNA foundation models to extract sequence representations and differed only in their DNA sequence encoders, while sharing the same ATAC-seq and CTCF ChIP-seq signal encoders and a joint representation*

decoder. The results show that SUCCEED achieves the best performance among all methods in predicting cell-type-specific three-dimensional chromatin architecture, with Sei—trained in a supervised manner on large-scale functional genomics data—exhibiting comparably strong performance (Supplementary Fig. 13, Supplementary Fig. 14). In contrast, Enformer performs less well, and the remaining self-supervised models show similar performance to Enformer but are consistently inferior to SUCCEED and Sei (Supplementary Fig. 13, Supplementary Fig. 14).”

<u>Newly added figure:</u>

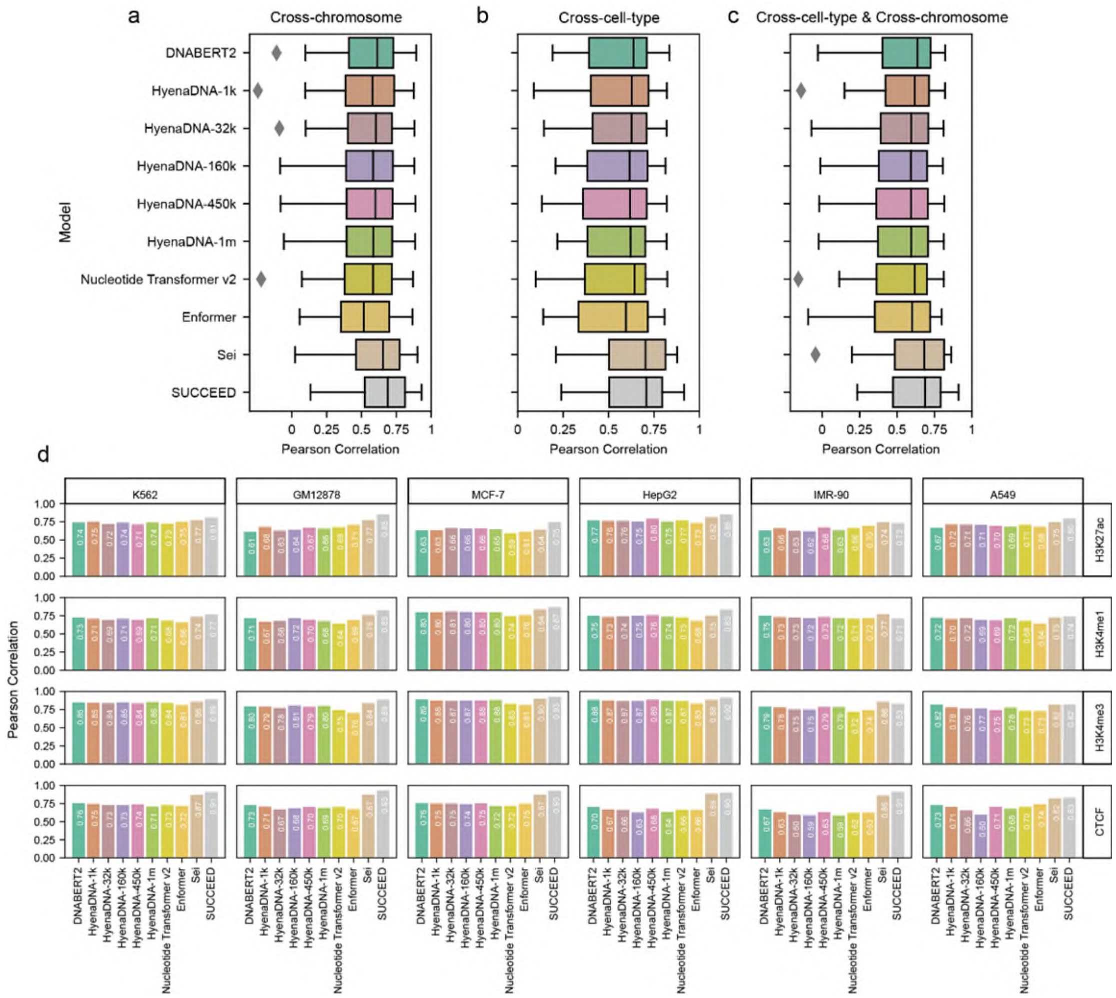

Supplementary Fig. 6 | Performance comparison of DNA foundation models for predicting cell type-specific epigenomic profiles. a,** Cross-chromosome generalization. Pearson correlation coefficients between predicted and experimentally measured epigenomic signals on held-out chromosomes (chr10 and chr21) in the training cell lines. **b,** Cross-cell type generalization. Pearson correlation coefficients between predicted and experimentally measured epigenomic signals in unseen cell lines (IMR-90 and A549) evaluated on training chromosomes (excluding chr10 and chr21). **c,

Cross-cell type and cross-chromosome generalization. Pearson correlation coefficients between predicted and experimentally measured epigenomic signals on held-out chromosomes (chr10 and chr21) in unseen cell lines (IMR-90 and A549). ***d,*** *Performance comparison of models across multiple commonly profiled epigenomic marks in different cell lines.*

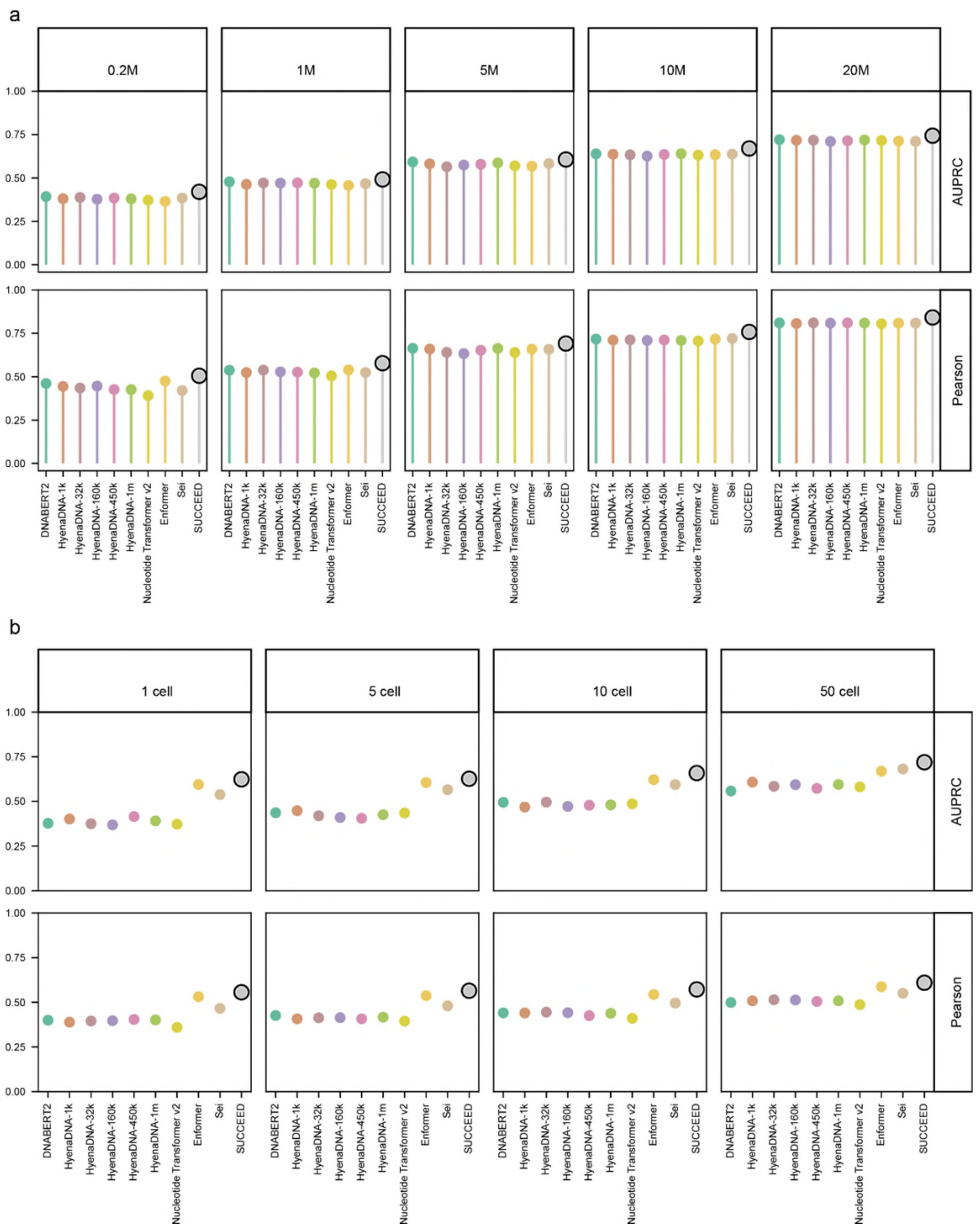

Supplementary Fig. 10 | Performance comparison of DNA foundation models for denoising and enhancing chromatin accessibility data. a, *Bulk ATAC-seq denoising and enhancement. Comparison of area under the precision–recall curve (AUPRC) and*

Pearson correlation coefficients between predicted and experimentally measured signals across different noise levels. ***b,*** *Single-cell ATAC-seq enhancement. Comparison of AUPRC and Pearson correlation coefficients between predicted and experimentally measured chromatin accessibility signals across varying cellular coverage levels.*

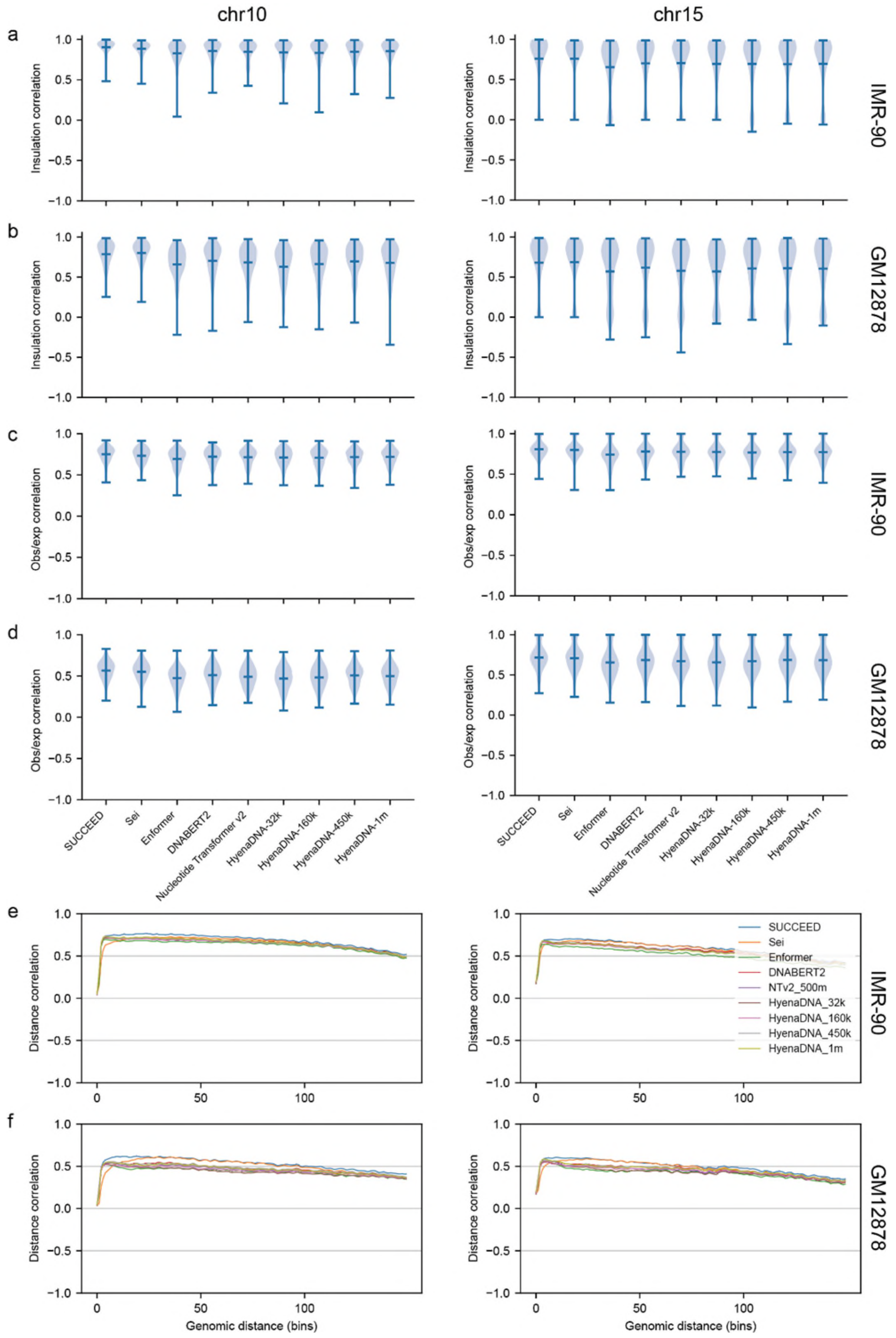

Supplementary Fig. 13 | Performance comparison of DNA foundation models for

predicting cell-type-specific 3D chromatin organization. a, *Comparison of insulation score concordance between predicted and experimentally measured Hi-C contact maps on held-out chromosomes in the training cell line IMR-90.* ***b,*** *Comparison of insulation score concordance between predicted and experimentally measured Hi-C contact maps on held-out chromosomes in the unseen cell line GM12878.* ***c,*** *Comparison of observed/expected (O/E) contact frequency concordance between predicted and experimentally measured Hi-C contact maps on held-out chromosomes in the training cell line IMR-90.* ***d,*** *Comparison of observed/expected (O/E) contact frequency concordance between predicted and experimentally measured Hi-C contact maps on held-out chromosomes in the unseen cell line GM12878.* ***e,*** *Comparison of distance-stratified Pearson correlation coefficients between predicted and experimentally measured Hi-C contact maps on held-out chromosomes in the training cell line IMR-90.* ***f,*** *Comparison of distance-stratified Pearson correlation coefficients between predicted and experimentally measured Hi-C contact maps on held-out chromosomes in the unseen cell line GM12878.*

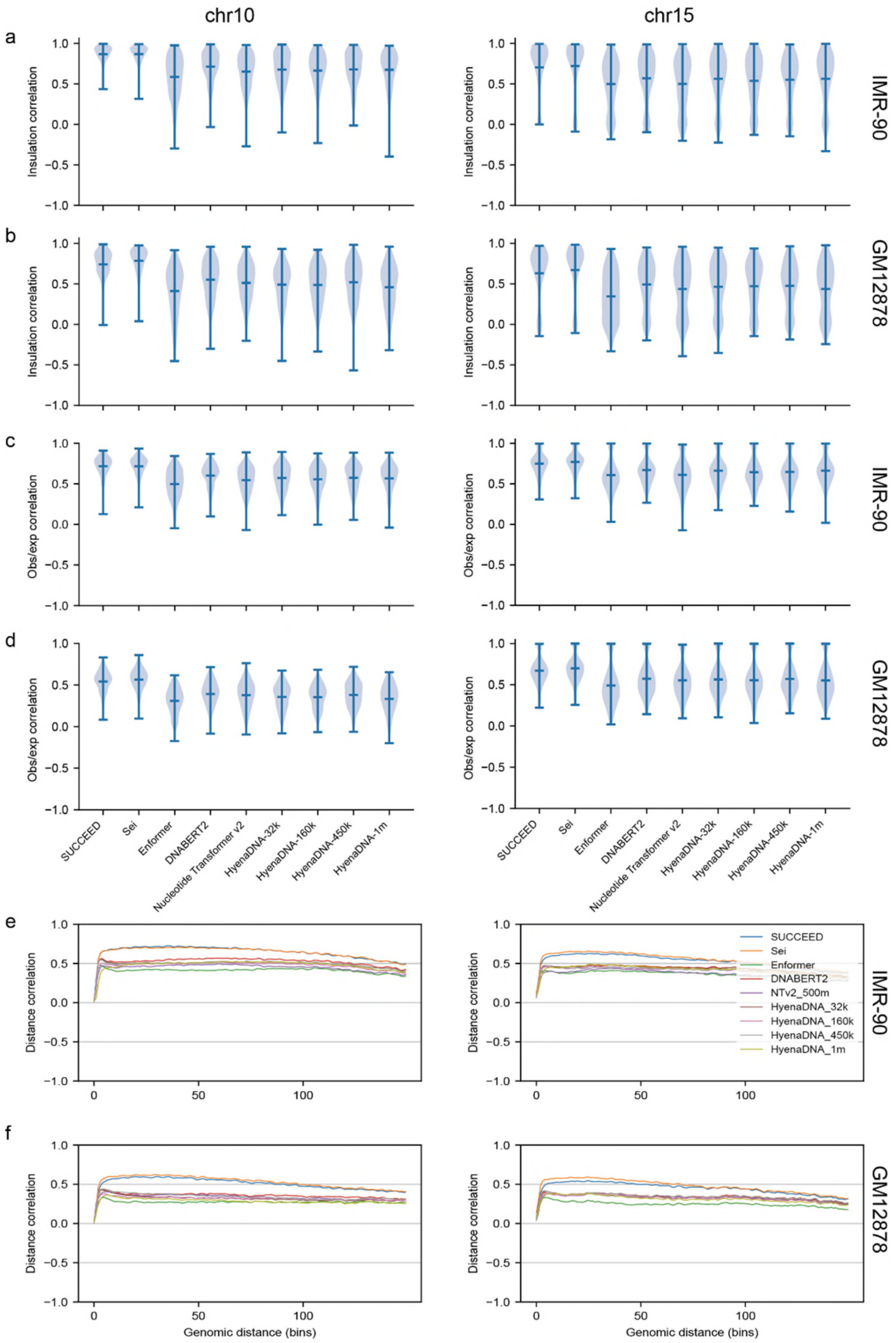

Supplementary Fig. 14 | Performance comparison of DNA foundation models for predicting cell type–specific 3D chromatin organization from single-cell ATAC-seq

data.* *a,* *Comparison of insulation score concordance between Hi-C contact maps predicted from scATAC-seq and experimentally measured Hi-C data on held-out chromosomes in the training cell line IMR-90.* ***b, *Comparison of insulation score concordance between Hi-C contact maps predicted from scATAC-seq and experimentally measured Hi-C data on held-out chromosomes in the unseen cell line GM12878.* ***c,*** *Comparison of observed/expected (O/E) contact frequency concordance between Hi-C contact maps predicted from scATAC-seq and experimentally measured Hi-C data on held-out chromosomes in the training cell line IMR-90.* ***d,*** *Comparison of observed/expected (O/E) contact frequency concordance between Hi-C contact maps predicted from scATAC-seq and experimentally measured Hi-C data on held-out chromosomes in the unseen cell line GM12878.* ***e,*** *Comparison of distance-stratified Pearson correlation coefficients between Hi-C contact maps predicted from scATAC-seq and experimentally measured Hi-C data on held-out chromosomes in the training cell line IMR-90.* ***f,*** *Comparison of distance-stratified Pearson correlation coefficients between Hi-C contact maps predicted from scATAC-seq and experimentally measured Hi-C data on held-out chromosomes in the unseen cell line GM12878.*

- Second, the description of methods and results are usually very short. There is no precise description of the different models, no discussion of the choices in the architectures and no assessment of these decisions. This should be done at least for the main SUCCEED model. For example, what is the benefit in term of accuracy or training time to replace ReLU/GeLU by SwiGLU/SiLU? What gain in accuracy the rotary positional embedding has allowed? What is the effect of the replacement of the LayerNorm by RMSNorm?

Response:

We sincerely thank the reviewer for this important suggestion. We acknowledge that the previous version did not provide sufficient detail regarding the SUCCEED model architecture, the rationale behind our design choices, and their empirical assessment. In the revised manuscript, we have substantially expanded the Methods section to provide a comprehensive description of the model architecture, including detailed specifications of the activation functions, normalization layers, and positional encoding schemes. We further clarify the motivations underlying these architectural choices and include additional implementation details and training configurations to ensure transparency and reproducibility.

In addition, we conducted an ablation study on the SUCCEED pre-trained model to quantitatively assess the impact of each architectural modification (Supplementary

Fig. 2). This analysis evaluates the effects of replacing ReLU/GELU with SiLU/SwiGLU, LayerNorm with RMSNorm, and removing rotary positional embeddings (RoPE).

The results show that each component provides modest but consistent improvements in both optimization behavior and final performance. Specifically, removing the SiLU/SwiGLU activation results in slightly higher training and validation losses and a small decrease in test Pearson correlation (from 0.627 to 0.625), whereas replacing RMSNorm with LayerNorm reduced the test correlation from 0.627 to 0.622. Similarly, removing RoPE slightly reduces test performance to 0.623 (Supplementary Fig. 2).

In addition to predictive performance, our architectural choices were guided by considerations of training stability and computational efficiency, drawing on recent advances in large language models such as LLaMA. SiLU/SwiGLU activations are widely adopted in modern transformer models due to their smooth gradients and stable optimization behavior, with computational costs comparable to ReLU or GeLU. RMSNorm avoids mean subtraction and is therefore slightly more computationally efficient than LayerNorm, while preserving effective normalization, which is advantageous for large-scale training. Rotary positional embeddings (RoPE) are parameter-free and integrate seamlessly into the attention mechanism without increasing parameter count or attention complexity. For the downstream model architectures, we adopted the encoder and decoder designs from state-of-the-art methods for each task, as these frameworks have been validated to deliver robust and effective performance in prior studies.

While none of these architectural changes alone yields a dramatic performance gain, the full SUCCEED architecture consistently achieves the best results across loss and correlation metrics. These findings indicate that the selected design choices contribute incremental but complementary benefits that improve training stability and downstream performance when combined.

All corresponding details and visualizations have been added to the revised manuscript.

<u>**Revised manuscript:**</u>

Page 3, Line 73: "*Specifically, the architecture consists of four main components: a convolutional frontend for early motif extraction, a multi-stage downsampling convolutional tower, a Transformer encoder for modeling long-range dependencies, and a pointwise prediction head with multitask outputs (Methods). A one-dimensional convolutional neural network identifies conserved local sequence motifs within cis-*

regulatory elements, such as promoters and enhancers. The downsampling tower enables the model to reduce spatial resolution progressively, allowing it to integrate information from different genomic regions effectively. These local features are passed to a Transformer module, which captures long-range dependencies by modeling interactions between distal genomic regions. Finally, a pointwise multi-task output head is used to support concurrent prediction of multiple regulatory signals, including chromatin accessibility, enhancer activity, and transcription factor binding. This hierarchical design enables joint encoding of local motif signals and the global regulatory context, thereby enhancing the model's ability to represent gene regulatory logic. To reduce computational cost, we optimized several architectural components by decreasing the number of convolutional kernels while increasing kernel stride to more effectively capture sequence motifs. In addition, motivated by recent advances in large language models, we systematically optimized key components of our architecture[12, 26, 27]. We incorporated the SwiGLU activation into the Transformer modules, where its gating mechanism adaptively modulates information flow, substantially enhancing representational flexibility and expressive capacity compared with piecewise-linear activations such as ReLU[28]. In parallel, we replaced conventional LayerNorm with RMSNorm. By normalizing activations solely based on their root mean square, RMSNorm eliminates mean subtraction, reduces computational overhead, and preserves the directional information of representations, thereby improving training stability in large-scale Transformer models[29]. Furthermore, we introduced rotary positional embeddings to jointly encode the relative and absolute positional information of regulatory elements within sequences, strengthening the model's ability to capture and exploit sequence structure[30]."

Newly added Reference:

"27.Grattafiori A, et al. The llama 3 herd of models. arXiv preprint arXiv:240721783, (2024).

28. Shazeer N. Glu variants improve transformer. arXiv preprint arXiv:200205202, (2020).

29. Zhang B, Sennrich R. Root mean square layer normalization. Advances in neural information processing systems ***32****, (2019).*

30. Su J, Ahmed M, Lu Y, Pan S, Bo W, Liu Y. Roformer: Enhanced transformer with rotary position embedding. Neurocomputing ***568****, 127063 (2024)."*

<u>Newly added figure</u>:

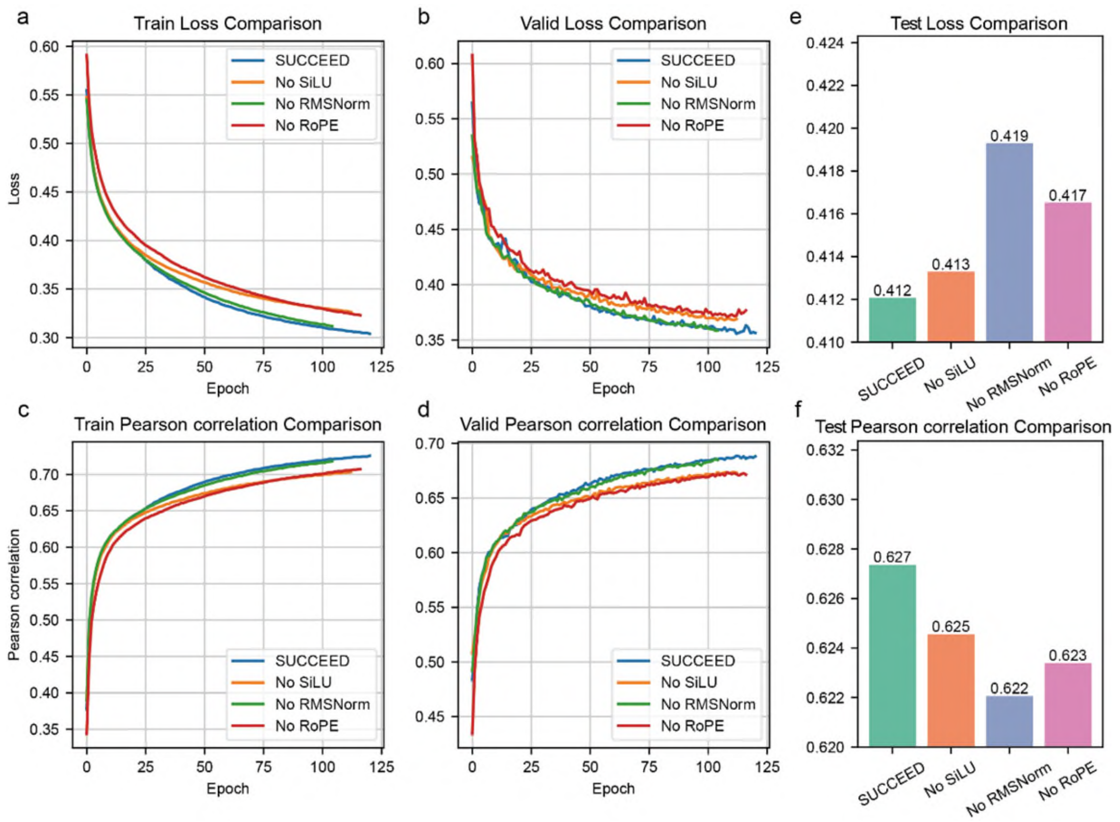

Supplementary Fig. 2 | Ablation analysis of the SUCCEED framework. ***a***, *Poisson negative log-likelihood (NLL) loss on the training set during training for different model variants. SUCCEED denotes the full model; No SiLU replaces the SiLU activation with ReLU; No RMSNorm replaces RMSNorm with LayerNorm; No RoPE replaces rotary positional encoding with sinusoidal absolute positional encoding.* ***b***, *Poisson NLL loss on the validation set during training for different model variants.* ***c***, *Pearson correlation coefficient between predicted and experimentally measured signals on the training set during training for different model variants.* ***d***, *Pearson correlation coefficient between predicted and experimentally measured signals on the validation set during training for different model variants.* ***e***, *Poisson NLL loss between predicted and experimentally measured signals on the test set for the best-performing model of each variant.* ***f***, *Pearson correlation coefficient between predicted and experimentally measured signals on the test set for the best-performing model of each variant.*

The same issue also applies on experiments, where the details of the exact plans are missing. For example, what is exactly done in the comparison of Fig. 2d? What are these 7 tasks? How are conducted the fine-tunings? How are measured the accuracies? Without these details and additional experiments, it is difficult to gauge the impact of the results or even their accuracy. For example, in the denoising task, what is exactly the gain of SUCCEED, i.e. what would be the accuracy of the same model trained using

only the noisy signal (without SUCCEED)?

<u>Response:</u>

We thank the reviewer for this important comment. We apologize for not providing sufficient detail in the original manuscript. Below, we provide a detailed clarification of the experimental design in Fig. 2d, the definition of the seven tasks, the fine-tuning protocol, the accuracy metrics, together with additional ablation experiments on the denoising task comparing SUCCEED against training using only noisy signals.

Fig. 2d presents a benchmark evaluation of representative genomic foundation models on a set of commonly used DNA sequence–based biological prediction tasks. These tasks have been widely adopted in prior work on DNA foundation models. In particular, seven downstream tasks are considered in this study: promoter prediction, non-TATA promoter prediction, TATA promoter prediction, core promoter prediction, non-TATA core promoter prediction, and TATA core promoter prediction, all formulated as binary classification tasks, as well as human splice site prediction, which is treated as a multi-task classification problem.

Because the input sequences for these downstream tasks are typically short (0.07k–15k bp), we removed the long-range interaction module of SUCCEED and fine-tuned the model by retaining only the convolutional backbone from the pretrained model. Given the limited sequence lengths, the receptive field of the convolutional module is sufficient to span the entire input. Following the GenBench protocol, a multilayer perceptron (MLP) was appended to the convolutional module as a task-specific classification head. Under the de novo setting, the same architecture and training protocol were used; however, the convolutional module was trained from random initialization without pretrained weights.

The reported results for HyenaDNA, DNABERT, DNABERT2, and Nucleotide Transformer are taken from the official GenBench repository (https://github.com/jimmylihui/GenBench), in which all models are fully fine-tuned following the GenBench protocol. Both SUCCEED fine-tuning and denovo training follow the same GenBench data splits, and all evaluations are performed on held-out test sets. We evaluate performance using the accuracy metric defined in GenBench for both binary and multi-class classification tasks:

$$Accuracy = \frac{TP + TN}{TP + TN + FP + FN}$$

where TP, TN, FP, and FN denote true positives, true negatives, false positives, and false negatives, respectively. We added a new subsection, "*Benchmark for self-*

supervised DNA language models”, to the Methods section to provide a more detailed description of the benchmarking procedure against other DNA foundation models.

For the chromatin accessibility denoising task, we conducted an ablation experiment to explicitly assess the contribution of SUCCEED. Specifically, we trained a model using only the noisy signal as input, without applying SUCCEED, and compared its performance against the SUCCEED-based model on the same held-out test data. As shown in Supplementary Fig. 8, removing SUCCEED leads to a consistent decrease in performance across evaluation metrics. This degradation is particularly pronounced under low sequencing depth and low cell-number regimes. For example, in previously unseen erythroid cell samples at a sequencing depth of 0.02 million reads, the SUCCEED-based model achieved an AUPRC of 0.38 in the peak-calling task, whereas the model trained without SUCCEED reached only 0.17. When the evaluation was restricted to a single chromosome (Chr10), a consistent performance gap was observed, with AUPRC values of 0.37 and 0.18, respectively. Further comparison of the Pearson correlation between the enhanced signals and high-quality reference data revealed the same trend at both the genome-wide level and on Chr10. In scATAC-seq signal enhancement tasks under low-cell-number conditions, SUCCEED-based models consistently outperformed models trained without SUCCEED across both NK and PBMC CD4+ cell types. These results indicate that SUCCEED contributes substantially to denoising performance, especially in low-coverage settings, suggesting that supervision from large-scale functional genomics data improves robustness in chromatin accessibility denoising tasks.

To improve clarity, we have revised the corresponding parts of the manuscript.

<u>Revised manuscript:</u>

Page 5, Line 139: “*We benchmarked SUCCEED against existing DNA foundation models on seven widely used genomic tasks that are commonly employed for model evaluation. Specifically, the tasks include promoter prediction, non-TATA promoter prediction, TATA promoter prediction, core promoter prediction, non-TATA core promoter prediction, and TATA core promoter prediction, all formulated as binary classification problems, as well as human splice-site prediction, which is treated as a multi-task classification task. Across all seven tasks, SUCCEED was evaluated on identical datasets under both fine-tuning and training-from-scratch settings (Methods). The fine-tuned SUCCEED model achieved a superior mean accuracy of 0.906, outperforming the model trained from scratch (mean accuracy of 0.891), thereby demonstrating the effectiveness of large-scale supervised pretraining for genomic prediction tasks (Fig. 2d). We compared SUCCEED with DNA foundation models on*

*these tasks and found that it achieves performance comparable to, or exceeding, that of larger self-supervised models across most genomic benchmarks (*Fig. 2d and Methods*).*"

Page 11, Line 356: "*To further evaluate the critical role of the prior information introduced by SUCCEED in denoising and signal enhancement, we trained control models that excluded SUCCEED on the same datasets. For example, in previously unseen erythroid cell samples at a sequencing depth of 0.2 million reads, the SUCCEED-based model achieved an AUPRC of 0.38 in the peak-calling task, whereas the model trained without SUCCEED reached only 0.17 (Supplementary Fig. 9a). When the evaluation was restricted to a single chromosome (Chr10), a consistent performance gap was observed, with AUPRC values of 0.37 and 0.18, respectively (Supplementary Fig. 9a). Further comparison of the Pearson correlation between the enhanced signals and high-quality reference data revealed the same trend at both the genome-wide level and on Chr10(Supplementary Fig. 9b). In scATAC-seq signal enhancement tasks under low-cell-number conditions, SUCCEED-based models consistently outperformed models trained without SUCCEED across both NK and PBMC CD4+ cell types (Supplementary Fig. 9c, d).*"

The Methods section has been expanded to clarify the definitions of the seven tasks, the fine-tuning and training-from-scratch protocols, and the evaluation metrics **(Page 21, Line 709)**.

Newly added figure:

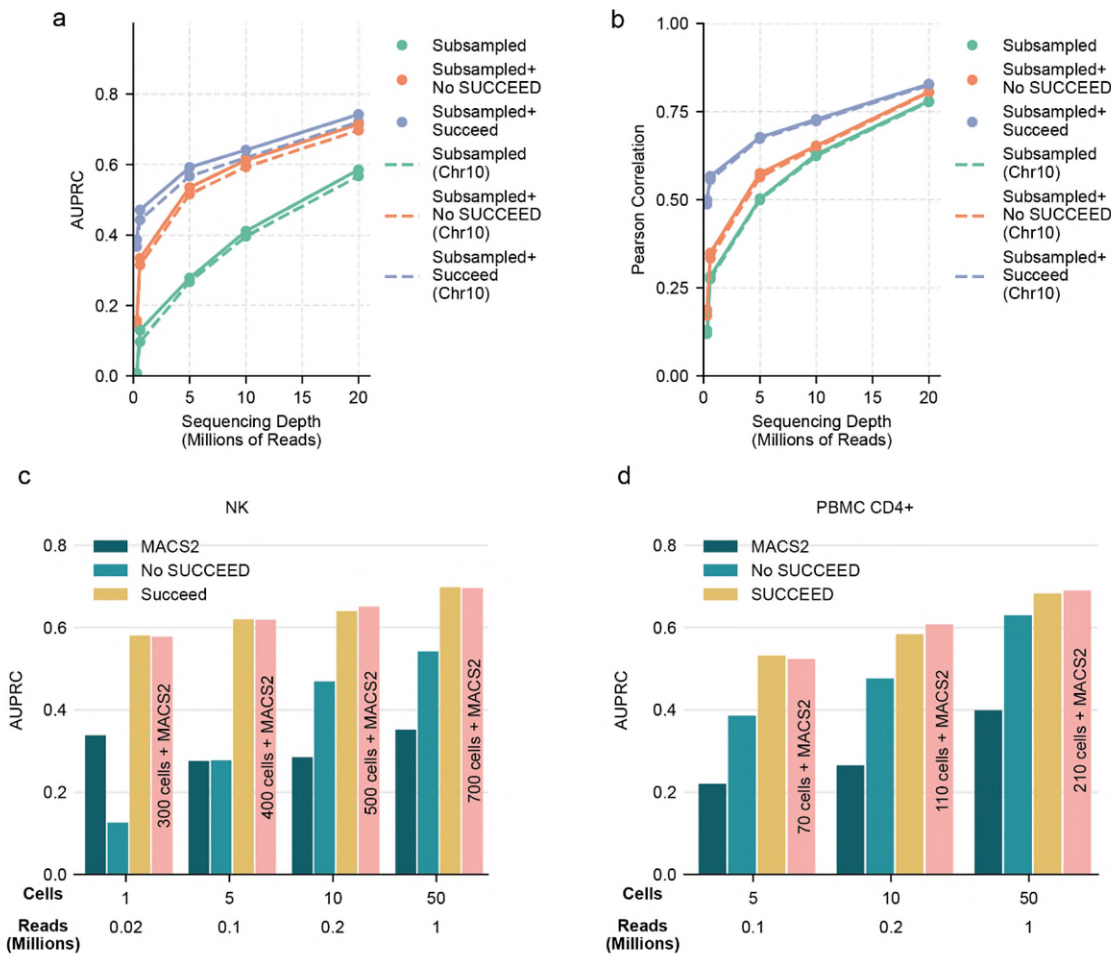

Supplementary Fig. 9 | Ablation analysis of model architectures and training strategies for denoising and enhancing chromatin accessibility data. a, *Area under the precision-recall curve (AUPRC) for chromatin accessibility peak denoising and enhancement in erythroid cells across different noise levels, comparing models trained with different training strategies.* ***b,*** *Pearson correlation coefficients between denoised ATAC-seq signals and experimentally measured high-quality signals in erythroid cells across different noise levels, comparing models trained with different training strategies.* ***c,*** *AUPRC for single-cell ATAC-seq peak denoising and enhancement in NK cells across varying numbers of cells, comparing models trained with different training strategies.* ***d,*** *Pearson correlation coefficients between denoised single-cell ATAC-seq signals and experimentally measured high-quality signals in PBMC CD4+ cells across varying numbers of cells, comparing models trained with different training strategies.*

Additionally, it seems that there are some problems in the experiments. Specifically, in the comparisons with EPCOT, for several marks such as H3K27ac, H3K4me1, MAFK, and CEBPB, the accuracy of EPCOT is better on cross-lines (Fig. 4.e) than on the cell

lines used during training (4.c & 4.d), which is quite suspect IMO.

Response:

We thank the reviewer for pointing out this inconsistency. We traced this issue to a mismatch in input signal preprocessing when reproducing the EPCOT baseline. Specifically, our initial implementation used ENCODE-provided bigWig files, which are typically normalized by sequencing depth or signal significance, whereas EPCOT's original pipeline employs RPGC-normalized DNase-seq/ATAC-seq signals. This discrepancy alters the signal distribution and dynamic range and affects different cell lines to varying extents. As a result, for some training cell lines, the input features deviated from those used during EPCOT's original training, whereas certain cross-cell-line datasets happened to be more closely aligned, leading to the apparent performance inversion. To address this issue, we re-evaluated the EPCOT baseline using RPGC-normalized DNase-seq or ATAC-seq signals. Specifically, we downloaded the processed data from the link provided in the original EPCOT publication and generated predictions using the publicly available pretrained model (Supplementary Table 3). We updated all affected results and figures (Fig. 4b-g and Supplementary Fig. 5d-g). After correction, EPCOT's performance became consistent between training and cross-cell-line evaluations, and the anomalous pattern was no longer observed. Under this unified and fair preprocessing setting, SUCCEED continues to outperform EPCOT across most tasks. The main conclusions of the study therefore remain unchanged. In addition, we systematically re-examined the evaluation protocols for all remaining downstream tasks and conducted multiple independent rounds of model training and assessment to minimize the impact of implementation- and evaluation-related variability on the reported results.

In the revised manuscript, we provide a comprehensive and detailed description of the benchmarking procedure between SUCCEED and EPCOT (Supplementary Table 4 and Methods).

Revised figure:

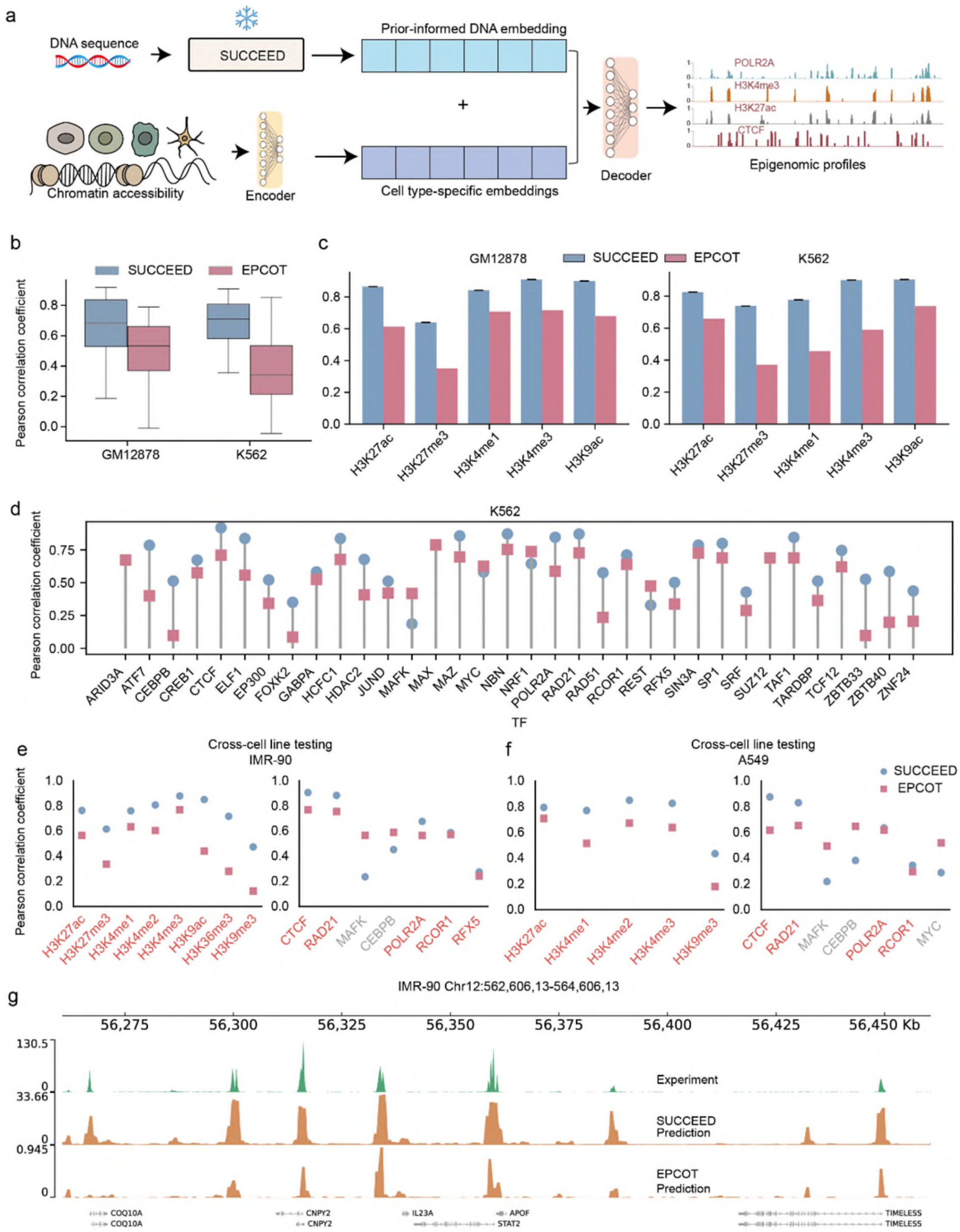

Fig. 4 | Performance of SUCCEED in predicting epigenomic landscapes surpasses other models. a, The SUCCEED framework for predicting cell type-specific epigenomic landscapes. The model takes 1,048,576 bp DNA sequences as input, incorporating cell type-specific chromatin accessibility data. The DNA sequence is encoded through the frozen parameters of SUCCEED, while chromatin accessibility data is processed by a Conv1D-only encoder. The two representations are concatenated and input into a classification head to predict various cell type-specific epigenomic landscapes. b, Performance comparison between SUCCEED and EPCOT on an independent test set,

evaluated using the Pearson correlation coefficient. c, Performance comparison between SUCCEED and EPCOT for predicting multiple histone modification landscapes on independent test sets from different cell lines. The x-axis represents histone modification markers, and the y-axis shows Pearson correlation coefficients. d, Performance comparison between SUCCEED and EPCOT for predicting multiple transcription factors in the K562 cell line. The x-axis represents transcription factors, and the y-axis shows Pearson correlation coefficients. e, f, Performance comparison of SUCCEED and EPCOT across cell types (unseen cell lines). Histone modifications are shown on the left, and transcription factors on the right. The red areas on the x-axis indicate that SUCCEED outperforms EPCOT, while the gray areas represent the opposite. g, Representative examples of observed versus predicted genomic tracks in the IMR-90 cell line, which the model has not encountered before.

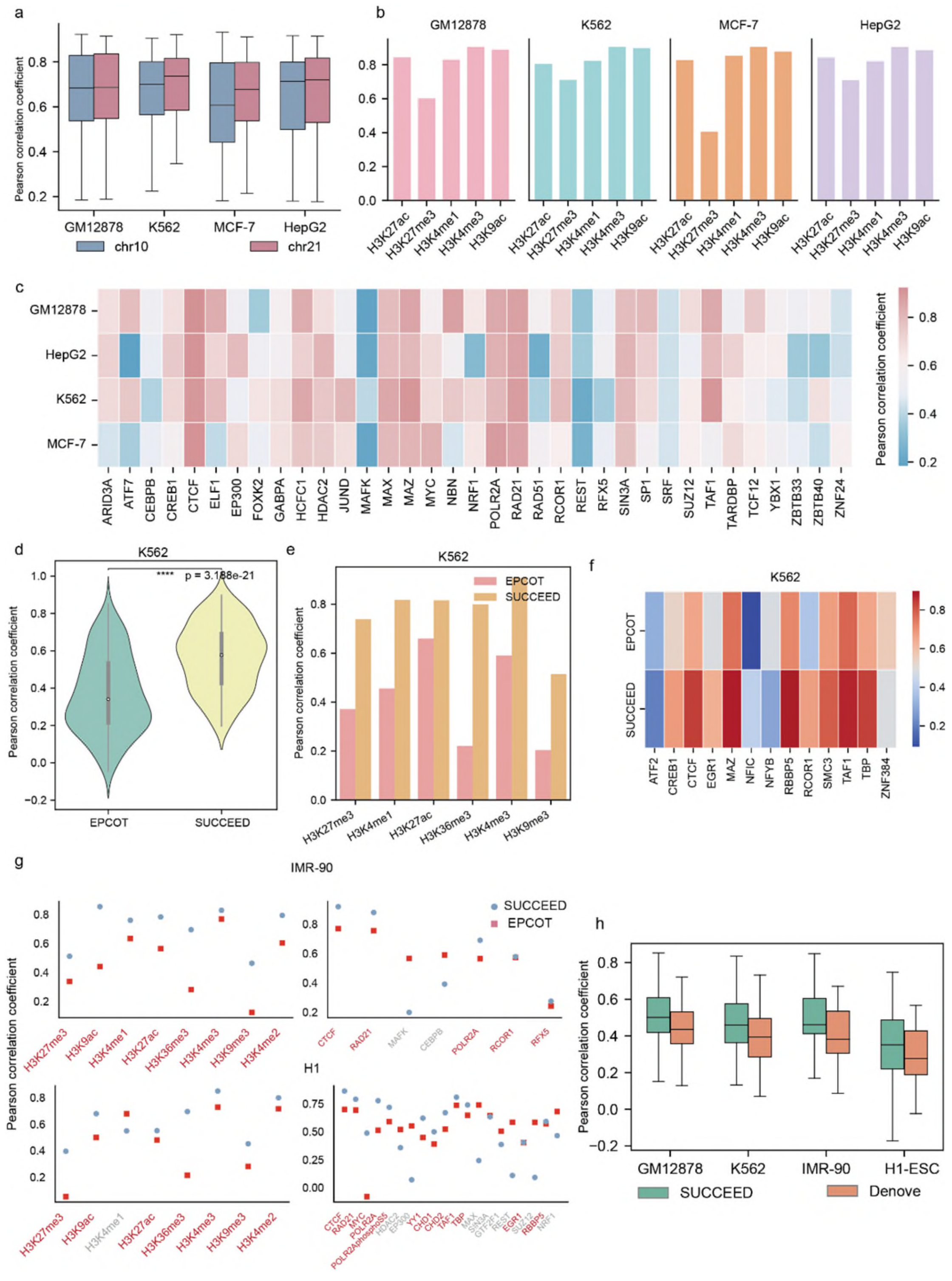

Fig. S5 | Performance comparison of SUCCEED and EPCOT with different training strategies. ***a****, Performance comparison of SUCCEED on independent test and validation sets across multiple datasets, evaluated using the Pearson correlation coefficient.* ***b****, Performance of SUCCEED in predicting histone modification markers across multiple cell lines.* ***c****, Performance of SUCCEED in predicting transcription factor binding sites across multiple cell lines.* ***d****, Overall performance of SUCCEED surpassing EPCOT when trained following the EPCOT strategy (evaluated using the*

Pearson correlation coefficient and t-test). ***e****, Performance of SUCCEED in predicting multiple histone modification markers under the EPCOT training strategy, outperforming EPCOT.* ***f****, Performance of SUCCEED in predicting multiple transcription factor binding sites under the EPCOT training strategy, outperforming EPCOT.* ***g****, Cross-cell type performance comparison of SUCCEED under the EPCOT training strategy, with red on the x-axis indicating that SUCCEED outperforms EPCOT and gray indicating the opposite.* ***h****, Comparison of performance between SUCCEED using a pre-trained model (with frozen parameters) and a de novo trained sequence encoder under the EPCOT training strategy.*

Revised manuscript:

Page 24, Line 838: "*The EPCOT model weights used for benchmarking were obtained from the official repository (https://github.com/liu-bioinfo-lab/EPCOT), and the input datasets were downloaded from the sources referenced therein (Supplementary Table 4). All models were evaluated on an identical held-out test set, with chromosome 2 designated for validation, chromosomes 10 and 21 for testing, and all remaining chromosomes for training. Cross-chromosomal evaluations were performed on chromosomes 10 and 20 in the K562, GM12878, HepG2, and MCF-7 cell lines, and cross-cell-type evaluations were conducted in IMR-90, A549, and H1. Model performance was quantified using the Pearson correlation coefficient between predicted and observed signals.*"

Response to Reviewer #2

Response to Reviewer #2

This paper presents SUCCEED, a supervised multi-task DNA foundation model pre-trained on a massive scale of 6,389 ENCODE tracks. Departing from masked self-supervised learning approaches, SUCCEED employs a hybrid architecture that synergistically combines one-dimensional convolutional layers for local motif detection and Transformer layers for capturing long-range regulatory dependencies. This design enables the model to learn the complex syntax of cis-regulatory grammar directly from DNA sequence to functional outputs. Benchmarking demonstrates that SUCCEED achieves performance on par with or superior to the established Enformer model across various tasks like CAGE, TF binding, and histone modification prediction, despite being a more lightweight model. Crucially, the authors showcase SUCCEED's exceptional transferability and scalability. Through efficient fine-tuning strategies, the model generalizes robustly to unseen cell types, different sequence lengths, and resolutions. Its capabilities extend beyond standard prediction to more challenging applications: it effectively denoises and enhances both bulk and single-cell ATAC-seq data, outperforming AtacWorks; it accurately predicts cell-type-specific epigenomic landscapes (histone marks, TF binding), surpassing EPCOT; and it infers 3D chromatin architecture from DNA sequence and ATAC-seq data alone, rivaling or exceeding the performance of C. Origami without requiring CTCF ChIP-seq input. In conclusion, SUCCEED positions itself as a powerful, versatile, and scalable foundation model that unifies inference across multiple genomic tasks, modalities, and scales, leveraging large-scale supervised pre-training to capture a generalizable understanding of genomic regulatory logic.

Reviewer #2 (Remarks on code availability):
The code is running good and instruction is clear.

Response:

Thank you for the positive feedback. We are glad to hear that the code runs smoothly and the instructions are clear.

Minor Revision Comments:

This paper presents SUCCEED, a supervised multi-task DNA foundation model pre-trained on a massive scale of 6,389 ENCODE tracks. Departing from masked self-

supervised learning approaches, SUCCEED employs a hybrid architecture that synergistically combines one-dimensional convolutional layers for local motif detection and Transformer layers for capturing long-range regulatory dependencies. This design enables the model to learn the complex syntax of cis-regulatory grammar directly from DNA sequence to functional outputs. Benchmarking demonstrates that SUCCEED achieves performance on par with or superior to the established Enformer model across various tasks like CAGE, TF binding, and histone modification prediction, despite being a more lightweight model. Crucially, the authors showcase SUCCEED's exceptional transferability and scalability. Through efficient fine-tuning strategies, the model generalizes robustly to unseen cell types, different sequence lengths, and resolutions. Its capabilities extend beyond standard prediction to more challenging applications: it effectively denoises and enhances both bulk and single cell ATAC-seq data, outperforming AtacWorks; it accurately predicts cell-type-specific epigenomic landscapes (histone marks, TF binding), surpassing EPCOT; and it infers 3D chromatin architecture from DNA sequence and ATAC-seq data alone, rivaling or exceeding the performance of C. Origami without requiring CTCF ChIP-seq input. In conclusion, SUCCEED positions itself as a powerful, versatile, and scalable foundation model that unifies inference across multiple genomic tasks, modalities, and scales, leveraging large-scale supervised pre-training to capture a generalizable understanding of genomic regulatory logic. Some detailed suggestions below aim to enhance the study's scientific rigor. Addressing them would further solidify the conclusions and broaden the impact of this valuable research.

1. Considering the training on human data, it would be fascinating to explore the model's zero-shot or few-shot capabilities on data from model organisms. This could shed light on the conservation of the learned regulatory grammar and broaden its application in comparative genomics.

Response:

We thank the reviewer for this insightful and forward-looking suggestion. We fully agree that evaluating zero-shot or few-shot transfer to model organisms is an important direction for assessing the conservation of regulatory grammar and for extending the applicability of genomic foundation models in comparative genomics.

In the original study, SUCCEED was trained exclusively on human genomic and epigenomic data, and cross-species zero-shot or few-shot evaluations were not included. In the revised manuscript, we have now added zero-shot cross-species analyses to explicitly address this point. Specifically, we evaluated SUCCEED in a zero-shot setting on multiple downstream tasks across species, including prediction of cell-type-

specific epigenomic profiles (Supplementary Fig. 7), denoising and enhancement of chromatin accessibility signals (Supplementary Fig. 11, and prediction of cell-type-specific three-dimensional chromatin organization (Supplementary Fig. 15). Notably, despite being trained solely on human genomic and epigenomic data and without using any mouse data during training, SUCCEED is able to transfer across species on these downstream tasks. These results suggest that SUCCEED captures regulatory representations that are at least partially conserved across species, highlighting its potential for cross-species analysis and comparative genomics.

To improve clarity, we have revised the corresponding parts of the manuscript.

Revised manuscript:

Page 9, Line 297: "*SUCCEED was pre-trained and fine-tuned exclusively on large-scale human functional genomics data. To assess whether the learned prior knowledge generalizes across species, we performed zero-shot transfer evaluations in multiple mouse tissues, including heart, liver, kidney and lung (Methods and Supplementary Table 5). Despite having no exposure to mouse DNA sequences or functional genomics data, SUCCEED achieved high accuracy across several epigenomic signal prediction tasks, particularly for CTCF and H3K27ac (Supplementary Fig. 7). These results indicate that key aspects of genomic regulatory grammar are functionally conserved between human and mouse. Overall, these results demonstrate that SUCCEED effectively leverages regulatory grammar priors learned from large-scale external datasets to accurately predict cell-type-specific epigenomic landscapes and to generalize across species in a zero-shot manner. This capability highlights its potential utility for studying transcriptional regulation in rare cell types and model organisms.*"

Page 11, Line 381: "*To evaluate the zero-shot cross-species transferability of SUCCEED in denoising and signal enhancement, we applied the model to ATAC-seq data from early mouse embryonic development. Despite the absence of mouse data during training, SUCCEED effectively denoised and enhanced chromatin accessibility signals (Supplementary Fig. 11a). Notably, even under low-coverage conditions, the enhanced signals remained highly correlated with the original sequencing data (Supplementary Fig. 11b). Collectively, these results demonstrate that SUCCEED leverages regulatory grammar priors to robustly denoise and enhance chromatin accessibility across bulk, single-cell, and low-coverage ATAC-seq data, while generalizing across species, thereby providing a unified and scalable framework for high-resolution epigenomic profiling.*"

Page 13, Line 454: "*We further evaluated SUCCEED's ability to predict three-*

dimensional chromatin architecture across species in a zero-shot setting (Supplementary Fig. 15). Specifically, we applied SUCCEED, trained exclusively on human data, to mouse early embryonic stages (8-cell and mESC) and the mouse cell line Patski (Supplementary Table 5). The results show that, using only ATAC-seq signals together with learned sequence representations, the predicted Hi-C maps exhibit high concordance with experimentally measured data in terms of insulation scores, suggesting a degree of structural conservation in topologically associating domain (TAD) formation between human and mouse (Supplementary Fig. 15a). In contrast, the correlation between predicted and experimental Hi-C data at the observed/expected level is lower than that achieved on human datasets, indicating the presence of species-specific differences in local chromatin architecture. We performed a visual comparison between the predicted Hi-C maps and experimentally measured Hi-C data on mouse chromosome 3. The results show that, under a zero-shot setting, the model can accurately reconstruct three-dimensional chromatin organization across different mouse cell types and tissues using only ATAC-seq signals, demonstrating robust cross-species generalization (Supplementary Fig. 15b)."

<u>Newly added figure</u>:

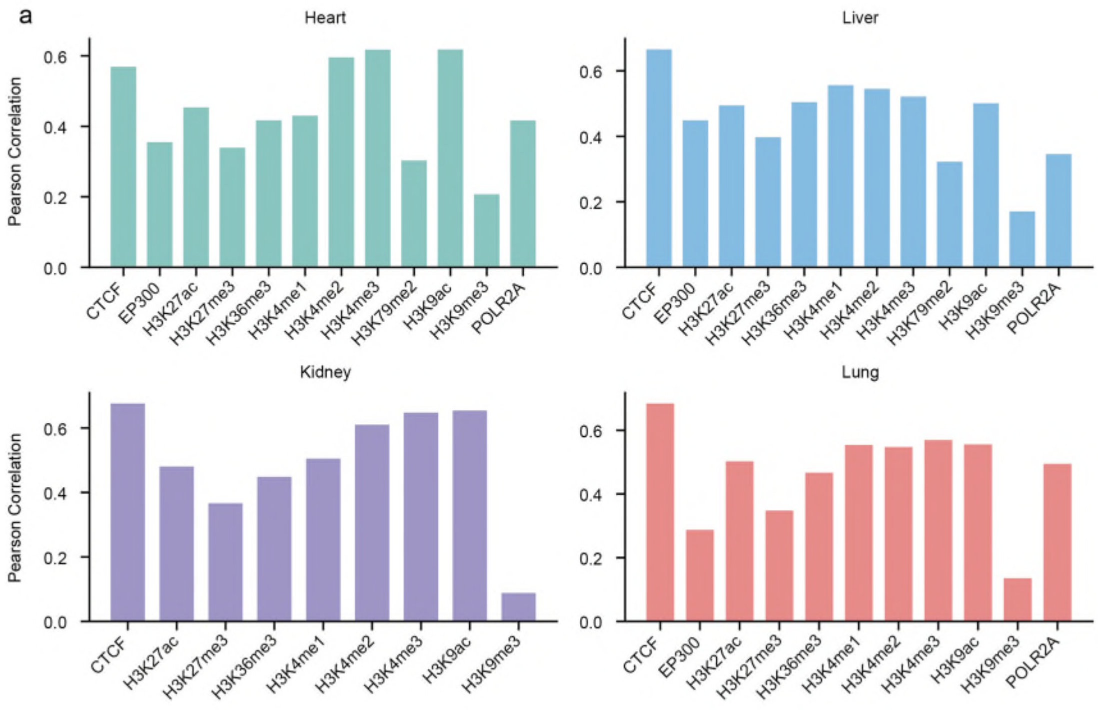

Supplementary Fig. 7 | Cross-species zero-shot prediction of mouse epigenomic landscapes by SUCCEED. a, *Pearson correlation coefficients between epigenomic signals predicted by SUCCEED in a zero-shot manner and experimentally measured*

epigenomic signals across four mouse tissues (heart, liver, kidney and lung).

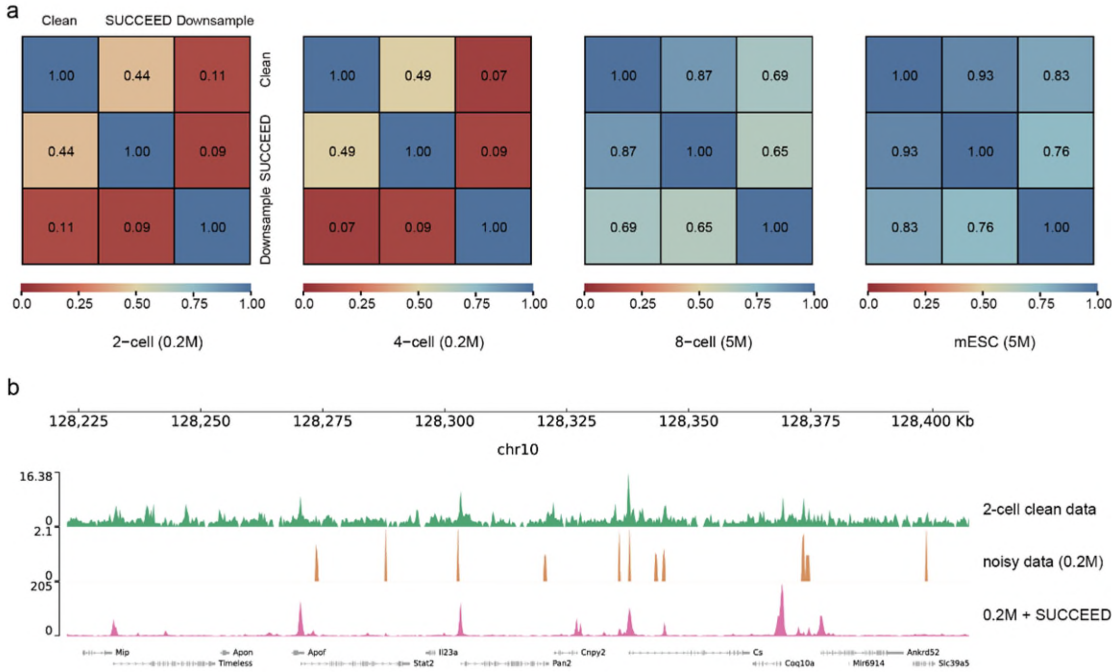

Supplementary Fig. 11 | Cross-species zero-shot denoising and enhancement of ATAC-seq data by SUCCEED. a, *Comparison of denoising and enhancement performance of SUCCEED during early mouse embryonic development across four stages (2-cell, 4-cell, 8-cell and mESC). Sequencing depths of 0.2M and 5M correspond to 200,000 and 5,000,000 reads, respectively.* ***b,*** *Comparison of chromatin accessibility signals after SUCCEED-based enhancement with low-coverage input signals and the original signals.*

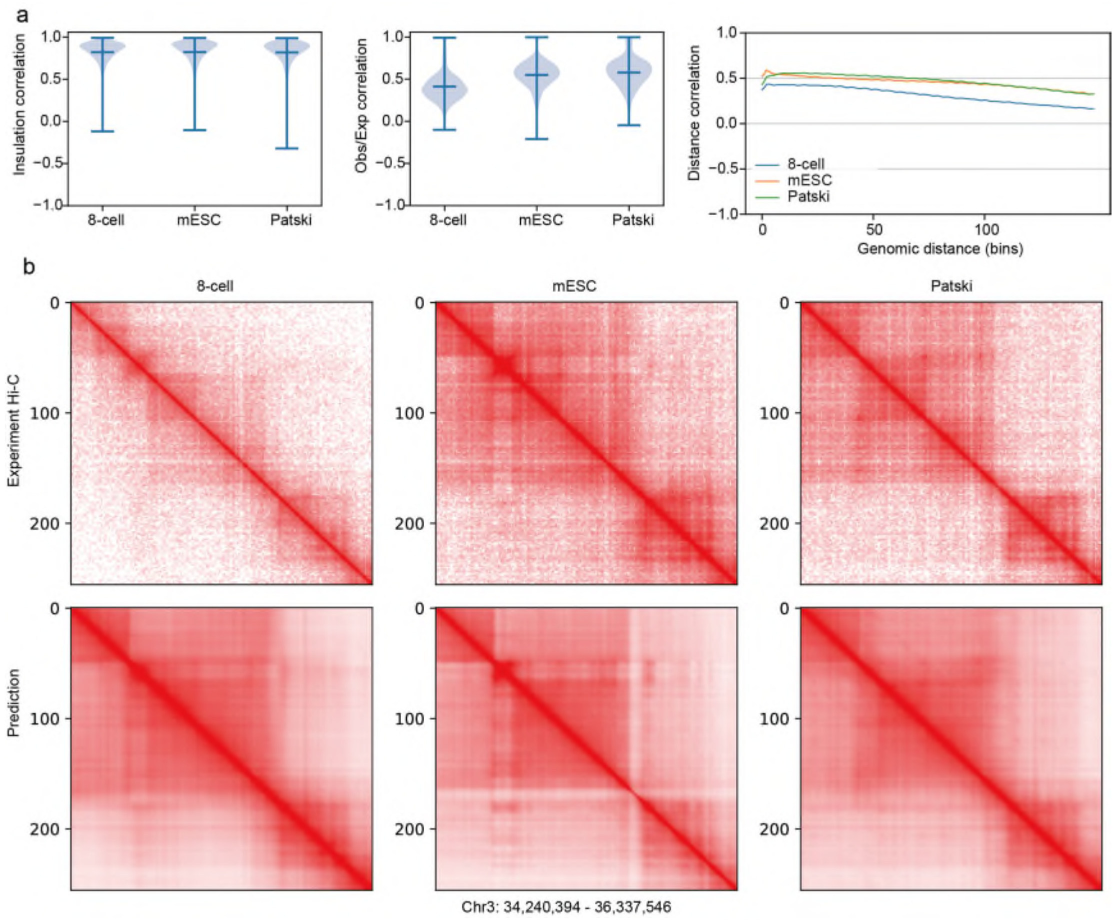

Supplementary Fig. 15 | Cross-species zero-shot prediction of 3D chromatin organization in mouse tissues and cell lines by SUCCEED.a, *Pearson correlation coefficients between predicted and experimentally measured Hi-C contact maps across different mouse tissues and cell lines (8-cell, mESC and Patski). From left to right, correlations are shown for insulation scores, observed/expected (O/E) contact frequencies and distance-stratified contact frequencies. Hi-C contact maps were predicted by SUCCEED from DNA sequence and ATAC-seq signals.* ***b,*** *Representative visualization of predicted and experimentally measured Hi-C contact maps on chromosome 3 (chr3) across different mouse tissues and cell lines.*

2. To further capture the hierarchical nature of genome regulation, it might be beneficial to investigate more explicit multi-scale modeling techniques within the architecture. This could potentially allow for even more seamless integration of features from nucleosome to topologically associating domain scales.

Response:

We thank the reviewer for this insightful suggestion. We agree that explicitly modeling

hierarchical genome organization across multiple scales—from nucleosomes to topologically associating domains—is a highly promising direction. In the current work, our model captures multi-scale regulatory signals by being fine-tuned on inputs with varying sequence lengths and resolutions, enabling the integration of information across different genomic scales. Nevertheless, we acknowledge that this strategy does not constitute an explicit multi-scale modeling architecture. We believe that incorporating dedicated multi-scale modules or hierarchical representations within a unified model would further enhance both the biological interpretability and modeling capacity of the framework. This point has been added to the Discussion section as an important direction for future work.

Revised manuscript:

Page 15, Line 513: "*Developing models that jointly represent multiple regulatory layers—including single-nucleotide sequence features, transcription factor binding, nucleosome occupancy, chromatin loops, TADs, and chromatin compartments—represents an important direction for future research. Such multi-scale frameworks will be critical for advancing sequence-based modeling of hierarchical gene regulation and may ultimately contribute to the realization of comprehensive in silico representations of cellular regulatory states. In parallel, although SUCCEED demonstrates strong zero-shot generalization across cell types and species, its transferability could be further extended by incorporating few-shot or meta-learning paradigms into the fine-tuning process. Such approaches may enable rapid adaptation to rare or previously uncharacterized cell types using only a small number of samples, complementing the zero-shot capabilities established here. Integrating few-shot or meta-learning strategies with large-scale pretraining therefore represents a promising direction for enhancing the flexibility and applicability of sequence-based regulatory models in data-scarce settings.*"

3. To empower biologists with deeper insights, integrating advanced model interpretation techniques as a standard component of the framework could help in precisely identifying causative sequence motifs and regulatory interactions underlying the predictions.

Response:

We thank the reviewer for this valuable suggestion. We agree that the interpretability of DNA sequence models is essential, as computational interpretation techniques can provide important insights into the effects of non-coding variants and facilitate the identification of functional genomic elements. In this work, our primary focus is on

predicting epigenomic profiles in unseen cell types by integrating DNA sequence information with other epigenomic signals, which we believe represents an important and complementary research direction. Nevertheless, to investigate whether the model has learned biologically meaningful representations rather than spurious correlations, we conducted interpretability analyses on the pretrained model.

Specifically, by analysing the activation maps of the first convolutional layer, we identified multiple known transcription factor binding motifs associated with functional regulatory elements (Supplementary Fig. 3). Moreover, gradient-based attribution analyses indicate that the model's predictions rely not only on local sequence features but also on the effective integration of long-range interactions within the DNA sequence (Supplementary Fig. 3).

Together, these results indicate that supervised pretraining on large-scale functional genomics data enables the model to learn representations that are directly related to biological function. With simple fine-tuning, such representations can be effectively transferred to downstream genomic prediction tasks.

To improve clarity, we have revised the corresponding parts of the manuscript.

Revised manuscript:

Page 5, Line 153: "*We next examined whether SUCCEED learns biologically meaningful representations rather than spurious correlations through a systematic interpretability analysis of the pretrained model. Specifically, we extracted activation maps from the first convolutional layer and aggregated subsequences whose activation values exceeded 80% of the maximal response, from which we derived position weight matrices (PWMs) for each convolutional filter. The resulting sequence patterns were then matched to known transcription factor motifs using TOMTOM from the MEME suite[34] against the JASPAR database[35], enabling the annotation of individual filters. This analysis identified multiple transcription factor motifs associated with functional regulatory elements (Supplementary Fig. 3a). In addition, to evaluate whether SUCCEED exploits long-range genomic interactions during prediction, we applied the Input × Gradient attribution method[36]. The results show that SUCCEED relies not only on local sequence features but also substantially incorporates long-range genomic information, indicating that the model captures regulatory signals across genomic scales (Supplementary Fig. 3b).*"

Page 21, Line 739: "*To dissect the sequence representations learned by the SUCCEED pre-trained model for DNA-to-epigenomic signal prediction and their contributions to distinct chromatin mark outputs, we performed interpretability analyses along two complementary axes: (i) extraction of sequence motifs associated*

with first-layer convolutional filters and (ii) gradient-based attribution using the Input × Gradient method to quantify nucleotide-resolution contributions.

Motifs were derived from feature maps (fmap) of the first convolutional layer. For each filter, we selected positions with activations exceeding 0.8× the filter-specific maximum, extracted the corresponding windows from the original one-hot–encoded sequences, aligned the subsequences, and constructed a position weight matrix (PWM) by aggregating nucleotide frequencies across aligned instances.

To localize the most predictive sequence positions, we computed Input × Gradient attributions using Captum. Gradients of the target output with respect to the input sequence were backpropagated and multiplied element-wise by the one-hot input, yielding attribution scores for each nucleotide position. These scores capture the sensitivity of model predictions to local sequence perturbations while restricting contributions to the observed nucleotide channels."

Newly added figure:

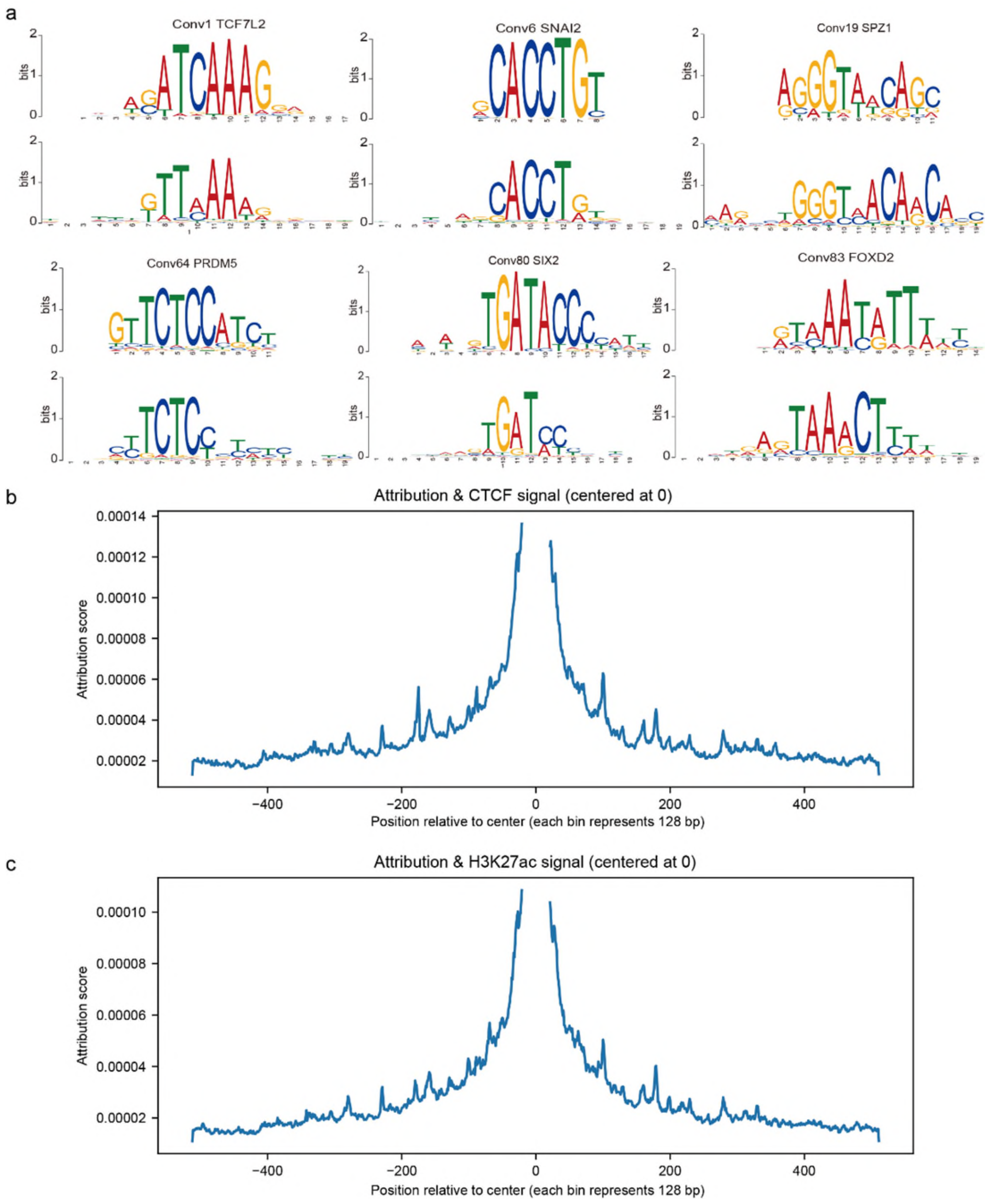

Supplementary Fig. 3 | Interpretability analysis of the SUCCEED pre-trained model. ***a,*** *Sequence motifs learned by the first convolutional layer of the SUCCEED model.* ***b,*** *Attribution scores computed using the Input × Gradient method for the CTCF ChIP-seq signal prediction task, aggregated across all samples; the x-axis denotes relative genomic positions.* ***c,*** *Attribution scores computed using the Input × Gradient method for the H3K27ac ChIP-seq signal prediction task, aggregated across all samples; the x-axis denotes relative genomic positions.*

Newly added Reference:

“34. Bailey TL, Johnson J, Grant CE, Noble WS. The MEME suite. *Nucleic acids research* **43**, W39-W49 (2015).
35. Rauluseviciute I, *et al.* JASPAR 2024: 20th anniversary of the open-access database of transcription factor binding profiles. *Nucleic acids research* **52**, D174-D182 (2024).
36. Shrikumar A, Greenside P, Shcherbina A, Kundaje A. Not just a black box: Learning important features through propagating activation differences. *arXiv preprint arXiv:160501713*, (2016).”

4. The model's remarkable transferability could be extended by incorporating few-shot or meta-learning paradigms into the fine-tuning process. This would be particularly valuable for predicting regulatory landscapes in rare or novel cell types where data is extremely scarce.

Response:

We thank the reviewer for this thoughtful and forward-looking suggestion. We agree that incorporating explicit few-shot or meta-learning paradigms into the fine-tuning process represents a promising direction for further extending the transferability of the proposed model, particularly for rare or previously uncharacterized cell types with extremely limited epigenomic data.

We note that the experimental setting considered in this study already corresponds to a low-data or few-shot transfer scenario: the model is trained on a limited number of cell types and systematically evaluated on previously unseen tissues or cell lines across multiple downstream tasks. Under this regime, supervised pretraining on large-scale functional genomics data enables robust cross-cell type and cross-species transfer, even when only a small amount of labeled data is available for fine-tuning.

Building on these findings, we have revised the Results and Discussion to more clearly articulate the few-shot nature of our framework and to highlight meta-learning–based fine-tuning as a natural and promising extension for future work.

Revised manuscript:

Page 14, Line 494: “*In addition, we find that SUCCEED exhibits zero-shot cross-species transferability, enabling accurate predictions in previously unseen cell types or species. This capability underscores its potential utility for investigating transcriptional regulation in rare cell types and model organisms.*”

Page 15, Line 519: “*In parallel, although SUCCEED demonstrates strong zero-shot generalization across cell types and species, its transferability could be further extended by incorporating few-shot or meta-learning paradigms into the fine-tuning process. Such approaches may enable rapid adaptation to rare or previously*

uncharacterized cell types using only a small number of samples, complementing the zero-shot capabilities established here. Integrating few-shot or meta-learning strategies with large-scale pretraining therefore represents a promising direction for enhancing the flexibility and applicability of sequence-based regulatory models in data-scarce settings."

5. The manuscript states in both the Methods and Results sections ("We obtained 6,389 human epigenomic profiles from ENCODE, excluding those generated under drug treatment or abnormal conditions") that 6,389 tracks were used for model pretraining. However, it does not provide the detailed selection criteria. It remains unclear whether biological/technical replicates were removed, whether batch effects across laboratories were normalized, and what specific filters were applied to exclude abnormal samples. I recommend that the authors provide a complete list of all tracks in the Supplementary Materials, along with experimental metadata and the normalization pipeline.

Response:

We thank the reviewer for raising this important point regarding data selection and transparency. We acknowledge that the previous description of the details regarding pre-training data processing was unclear.

For model pretraining, we curated 6,389 human epigenomic tracks from ENCODE by excluding experiments annotated in the ENCODE metadata as being performed under drug treatments, genetic perturbations, or other abnormal conditions. For experiments with multiple biological or technical replicates, we retained samples after merging the replicates originating from the same experiment.

To mitigate batch effects across different laboratories and experimental protocols, all tracks were processed using ENCODE's standardized normalization and preprocessing pipelines. Specifically, for ATAC-seq tracks, we used the Signal p-value bigWig files provided by ENCODE. The Signal p-value bigWig files were also used for histone modification and transcription factor ChIP-seq data, while for DNase-seq data, we retained the read-depth–normalized signal bigWig files, as described in the Methods section.

In response to the reviewer's suggestion, we have now provided a complete list of all pretraining tracks in the Supplementary Table 1, including ENCODE accession IDs, assay types, cell or tissue annotations, laboratory information, replicate identifiers, and relevant experimental metadata. These additions are intended to improve transparency and facilitate reproducibility.

6. In the Enformer benchmark section, the authors mention that they "retrained a SUCCEED model on the dataset provided in the original Enformer paper" and extended the input length to 196,608 bp. However, several comparisons appear to use inconsistent input lengths, resolutions, or preprocessing steps among SUCCEED, Enformer, EPCOT, and C.Origami, which may affect fairness. The authors should clearly describe, for each comparison, the training and evaluation protocol, including input length, whether model weights were frozen, batch size, optimizer, learning-rate schedule, early-stopping criteria, and random seeds. A supplementary table aligning these settings across models would substantially improve transparency and fairness.

Response:

We appreciate the reviewer for highlighting this issue. We acknowledge that the training and evaluation protocols across different models were not sufficiently specified in the original manuscript.

First, for the SUCCEED and Enformer benchmark, we downloaded the Basenji2 dataset from the link provided in the original Enformer paper. Following the procedure described in Enformer, we extended the input length of the dataset from 131,072 bp to 196,608 bp by symmetrically extending both ends using the hg38 reference genome. We retrained the SUCCEED model on this dataset with an input length of 196,608 bp. In contrast, EPCOT, AtacWorks, and C.Origami were evaluated using their originally reported input lengths and resolutions, as these are intrinsic design choices of the respective models and cannot be modified without re-architecting or retraining the models from scratch.

To improve transparency and fairness, we have now explicitly described the full training and evaluation protocol for each model comparison, including input length, output resolution, whether the model was trained from scratch or evaluated with frozen weights, batch size, optimizer, learning-rate schedule, early-stopping criteria, and random seed settings.

In addition, we have added a new supplementary table (Supplementary Table 3) that summarizes and aligns these settings across SUCCEED, Enformer, EPCOT, and C.Origami, clearly indicating which differences arise from inherent model constraints and which result from experimental choices. We believe that these additions substantially improve the clarity and interpretability of the benchmark results.

To improve clarity, we have revised the corresponding parts of the manuscript.

Revised manuscript:

Page 20, Line 677: "*We trained, evaluated, and tested the model on the same target datasets across all experiments, using the Poisson negative log-likelihood loss function adopted in Enformer and Basenji2. During model pretraining, we followed the data partitioning strategy used in Enformer and Basenji2, in which the genome is segmented into fixed-resolution samples and split into training, validation, and test sets using an 8:1:1 ratio. Two data augmentation strategies were applied during training: reverse-complementing the input sequences and introducing a random 1-3 bp shift. Optimization was performed using the AdamW algorithm in PyTorch with a learning rate of* 1×10^{-4}*, and gradients were clipped to a maximum global norm of 0.2. Early stopping was employed with validation loss as the monitoring metric and a patience of 20 epochs*"

Page 24, Line 823: "*The model takes 1,048,576 bp genomic sequences together with matched chromatin accessibility profiles as input. DNA sequences are encoded by the frozen SUCCEED model, whereas cell-type-specific accessibility signals are processed by an independent encoder. The resulting representations are concatenated and passed to a classification head to predict 46 histone-modification and transcription-factor-binding features. Training was performed with a batch size of 32 using the Adam optimizer and a learning rate of 0.001. Following the EPCOT training strategy, we also constructed a model predicting 208 epigenomic features, which shared the same architecture and hyperparameters, differing only in the dimensionality of the output layer. To quantify the contribution of pre-training, we performed an ablation experiment by training a model without SUCCEED pre-training, replacing the pre-trained sequence encoder with a randomly initialized one while keeping all other configurations unchanged. All models were optimized using the Poisson negative log-likelihood loss.*"

Page 26, Line 886: "*We employed a pretrained 131 kb-128 bp sequence encoder to extract DNA sequence representations, keeping its parameters frozen during downstream training. An independent encoder was used to extract features from low-coverage or low-quality noisy signals. The representations from both encoders were concatenated and passed through a linear layer for denoising, enhancement, and peak calling. To evaluate the effects of large-scale supervised pretraining, we performed an ablation study by training a sequence-processing model without pretraining as a control. During training, we used chromosomes 10 and 20 for testing and validation, respectively, with the remaining chromosomes allocated for training. The model was trained using mean squared error (MSE) and binary cross-entropy (BCE) loss functions, weighted equally, with a batch size of 32, AdamW optimizer, and a learning rate of 0.001. Early stopping with a patience of 20 epochs was applied. AtacWorks model*

weights were obtained from the official repository (https://github.com/NVIDIA-Genomics-Research/AtacWorks). This model predicts based on the coverage of each genomic base, disregarding DNA sequence information, and utilizes a ResNet-like architecture. The training loss function combined MSE, 1-Pearson correlation, and BCE (Supplementary Table 3)."

7. The manuscript reports many averaged performance metrics (e.g., PCC and AUPRC in Figures 2–5) without specifying the number of independent runs or providing confidence intervals/standard deviations. Given that deep learning models are sensitive to random initialization and data partitioning, this omission raises concerns about robustness. The authors should repeat each key comparison (particularly those with Enformer, EPCOT, AtacWorks, and C.Origami) at least five times and report mean ± SD or bootstrap 95% confidence intervals, including full tables in the Supplementary Materials to support reproducibility.

Response:

We thank the reviewer for raising this important point regarding robustness and reproducibility. We agree that deep learning models can be sensitive to random initialization and data partitioning, and that reporting uncertainty estimates is essential for a fair comparison.

In response, we repeated all key comparative experiments, including comparisons with Enformer, EPCOT, AtacWorks, and C.Origami, using multiple independent runs with different random seeds. Except for Enformer, which was run twice due to computational constraints, all other settings were evaluated with at least five independent runs. For each comparison, we now report the mean performance and standard deviation across runs.

We have updated the corresponding figures (Fig. 2-5) to reflect these aggregated statistics.

Revised figure:

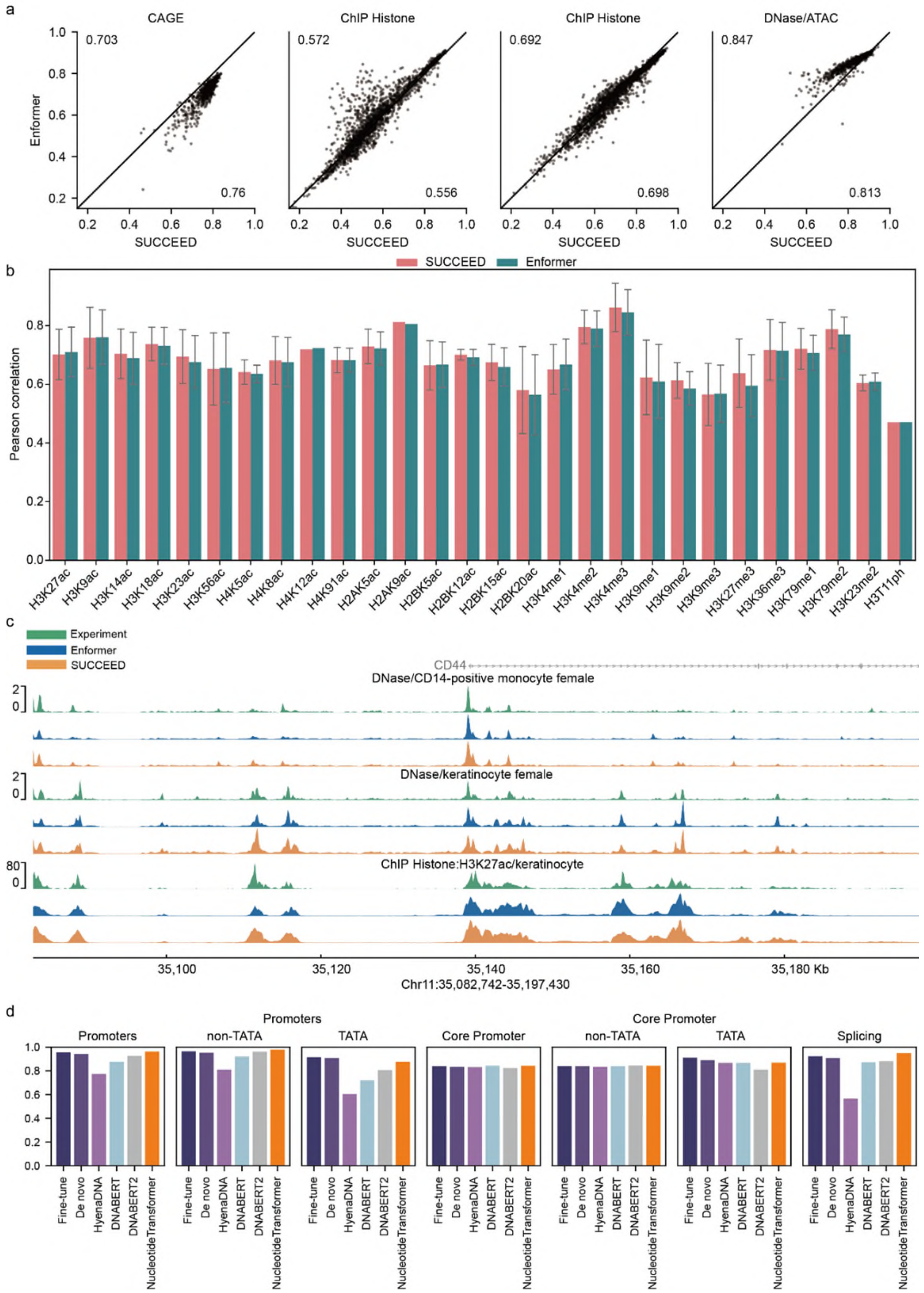

Fig. 2 | Model performance evaluation. ***a***, *Comparison of SUCCEED and Enformer results across 638 CAGE, 2131 transcription factor (TF) ChIP-seq, 1860 histone modification ChIP-seq, and 684 DNase-seq or ATAC-seq datasets.* ***b***, *Pearson correlation coefficients between SUCCEED and Enformer for various histone modification datasets. The x-axis represents specific histone modifications, while the y-*

axis shows the average Pearson correlation coefficient, with error bars representing the standard deviation (s.d.) of the results. ***c***, *Representative examples of predicted genomic trajectories at the CD44 locus in the test set, illustrating the consistency between SUCCEED and Enformer predictions and the true results. The three trajectories in each experiment share the same y-axis. d, SUCCEED was benchmarked against masked self-supervised genomic language models across seven representative genomic tasks, with de novo referring to models trained from scratch on a given task without leveraging pretrained parameters.*

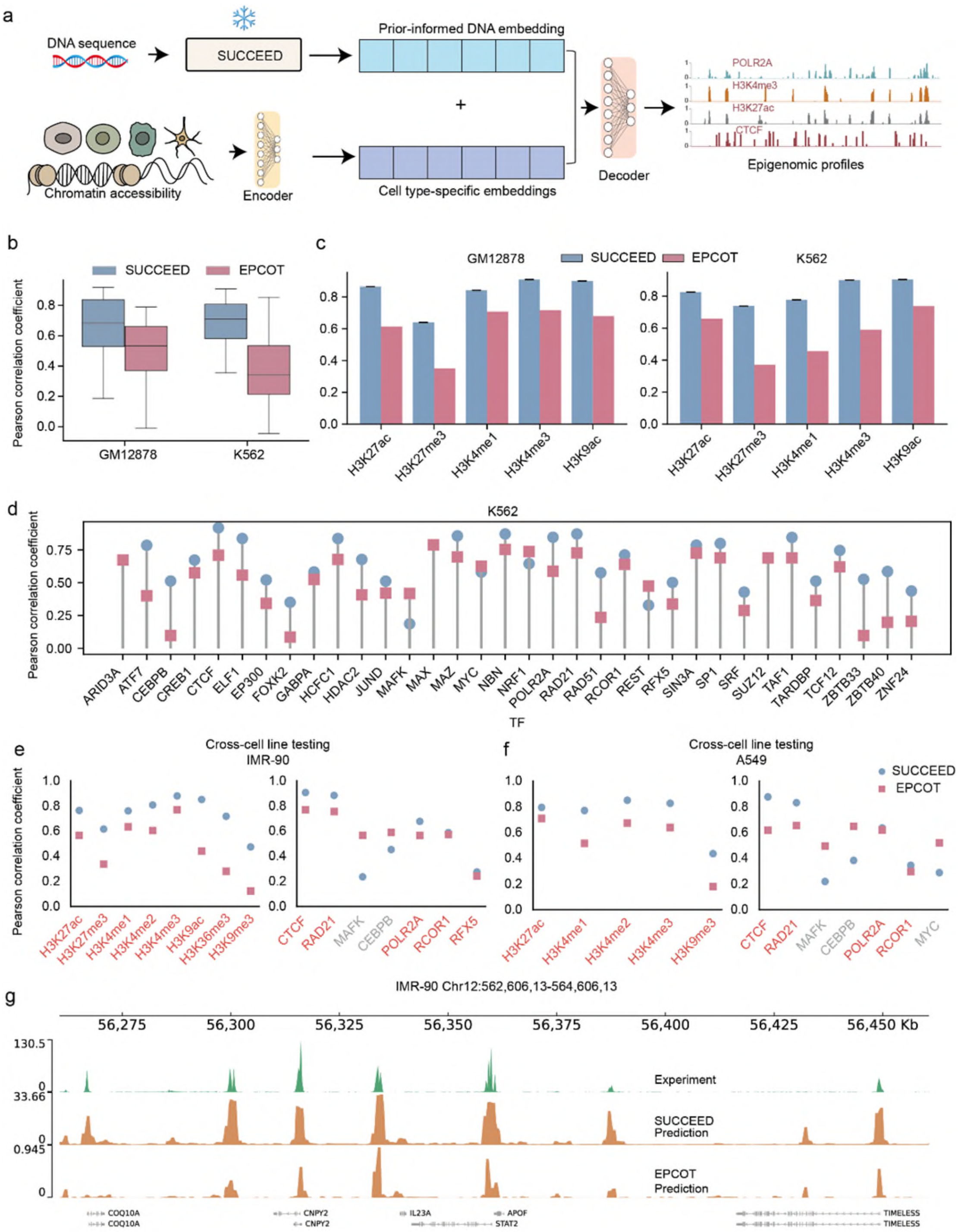

Fig. 4 | Performance of SUCCEED in predicting epigenomic landscapes surpasses other models. ***a***, *The SUCCEED framework for predicting cell type-specific epigenomic landscapes. The model takes 1,048,576 bp DNA sequences as input, incorporating cell type-specific chromatin accessibility data. The DNA sequence is encoded through the frozen parameters of SUCCEED, while chromatin accessibility data is processed by a Conv1D-only encoder. The two representations are concatenated and input into a classification head to predict various cell type-specific epigenomic landscapes.* ***b***, *Performance comparison between SUCCEED and EPCOT on an independent test set,*

evaluated using the Pearson correlation coefficient. ***c****, Performance comparison between SUCCEED and EPCOT for predicting multiple histone modification landscapes on independent test sets from different cell lines. The x-axis represents histone modification markers, and the y-axis shows Pearson correlation coefficients.* ***d****, Performance comparison between SUCCEED and EPCOT for predicting multiple transcription factors in the K562 cell line. The x-axis represents transcription factors, and the y-axis shows Pearson correlation coefficients.* ***e****,* ***f****, Performance comparison of SUCCEED and EPCOT across cell types (unseen cell lines). Histone modifications are shown on the left, and transcription factors on the right. The red areas on the x-axis indicate that SUCCEED outperforms EPCOT, while the gray areas represent the opposite.* ***g****, Representative examples of observed versus predicted genomic tracks in the IMR-90 cell line, which the model has not encountered before.*

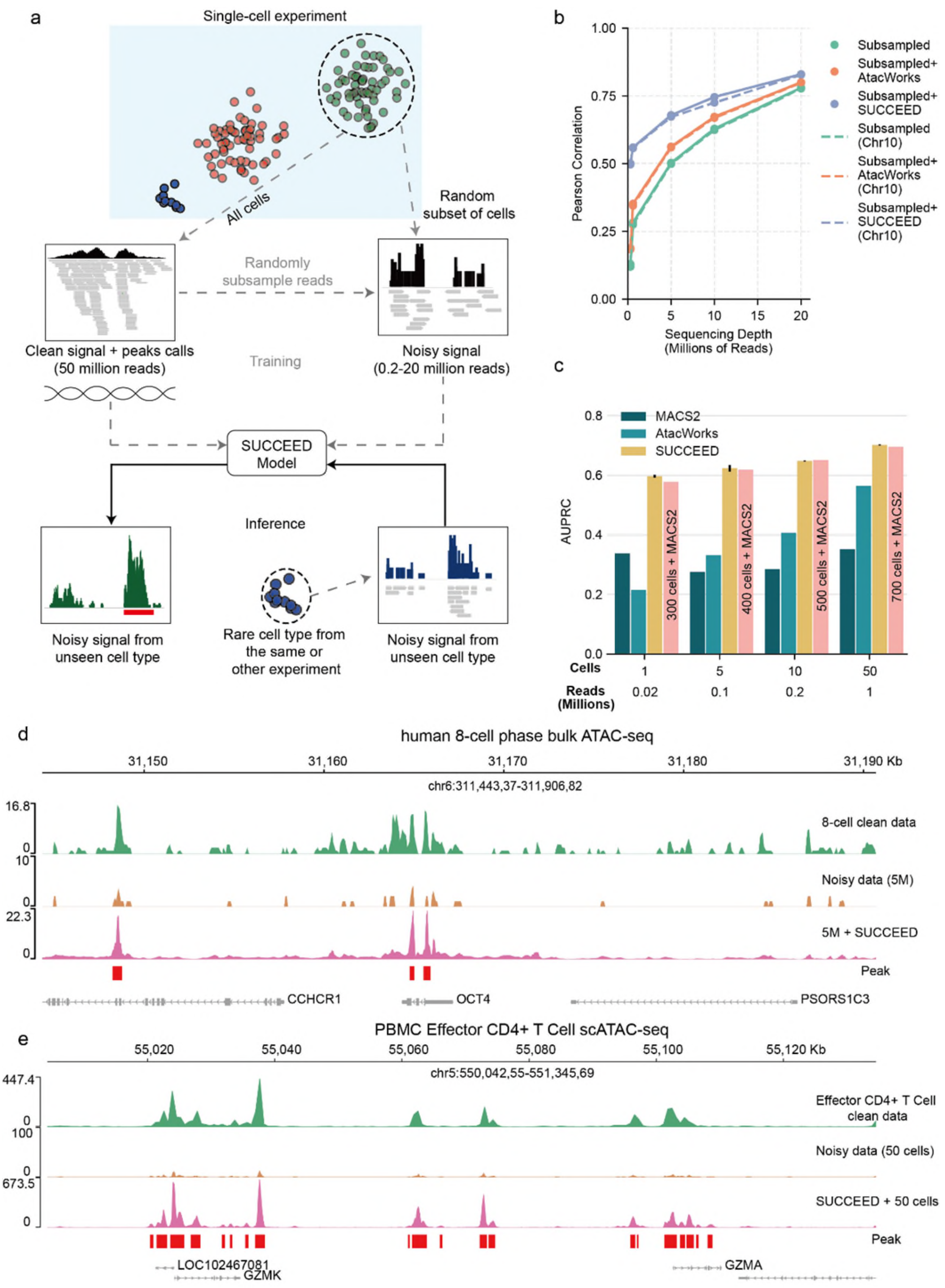

Fig. 5 | SUCCEED enhances chromatin accessibility data denoising and augmentation, outperforming other models. ***a***, *The SUCCEED framework for chromatin accessibility landscape denoising and enhancement. The model takes 131,072 bp DNA sequences as input, combined with low-quality or noisy chromatin accessibility data. The DNA sequence is encoded through the frozen parameters of SUCCEED, while the chromatin accessibility data is processed by a Conv1D-only encoder. The two representations are*

concatenated and input into a dense layer to generate clean chromatin accessibility data. ***b****, Comparison of SUCCEED and AtacWorks on various levels of subsampling (simulating noise and low quality), evaluated using the Pearson correlation coefficient. The solid line indicates whole-genome denoising and enhancement performance, while the dashed line represents performance on chromosome 10.* ***c****, Performance comparison of SUCCEED, AtacWorks, and MACS2 (using original input) in calling peaks on scATAC-seq data, with comparisons made at 1 cell, 5 cells, 10 cells, and 50 cells.* ***d****, Representative genomic tracks of raw, noisy, and SUCCEED-denoised ATAC-seq data from the 8-cell stage of human early embryonic development. These examples are derived from a dataset that was not included in the model training.* ***e****, Representative tracks from raw data, noisy data, and denoised data by SUCCEED in scATAC-seq data from PBMC Effector CD4+ T cells, focusing on the marker gene GZMK, in a model that has not seen this data before.*

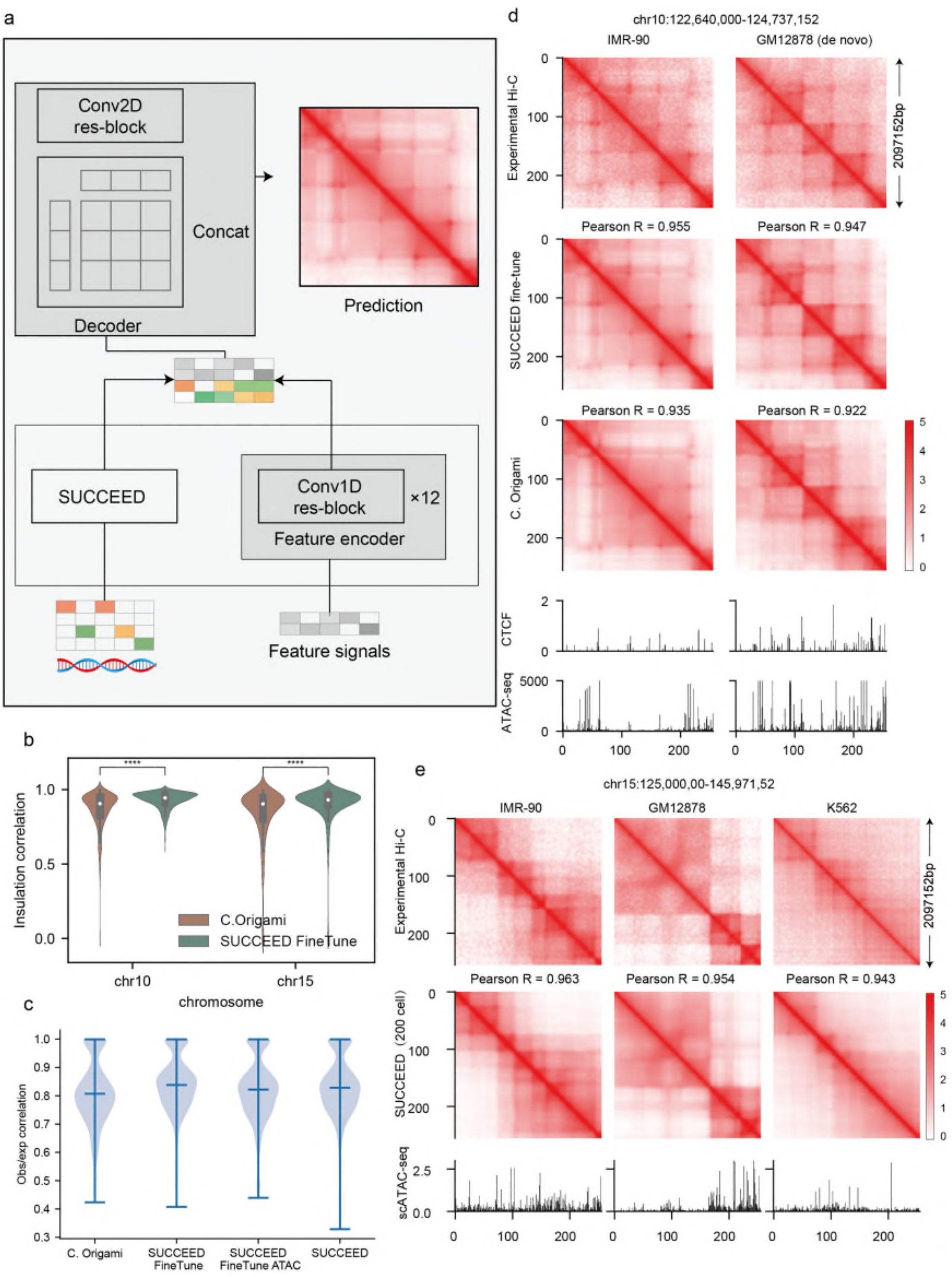

Fig. 6 | SUCCEED outperforms other methods in predicting cell type-specific chromatin 3D structures. ***a***, *The SUCCEED framework for predicting cell type-specific chromatin 3D structures. The model takes as input 2,097,152 bp DNA sequences with a resolution of 8192 bp, chromatin accessibility, and CTCF ChIP-seq data. DNA sequences are encoded by SUCCEED, while chromatin accessibility data are processed by C. Origami's feature encoder to generate compressed representations. These two representations are concatenated and passed through a decoder to predict chromatin*

3D structures. ***b****, Performance evaluation of SUCCEED and C. Origami on the IMR-90 test and validation sets, using Pearson correlation coefficients of insulation scores, with statistical comparison via t-test.* ***c****, Performance comparison of various training strategies (using Observed/Expected ratio). "SUCCEED fine-tune" denotes a training process where SUCCEED parameters are not frozen and are fine-tuned throughout the training. "SUCCEED fine-tune ATAC" refers to a model trained with only ATAC-seq and DNA sequences as input, where SUCCEED is fine-tuned during training. "SUCCEED" indicates that SUCCEED parameters are frozen during training, with only the Feature Encoder and Decoder being trained.* ***d****, Experimental Hi-C data, SUCCEED fine-tune predicted Hi-C, C. Origami predicted Hi-C, CTCF ChIP-seq, and ATAC-seq signals on chromosome 10 in IMR-90 and GM12878.* ***e****, Experimental Hi-C data, SUCCEED fine-tune predicted Hi-C using scATAC-seq and DNA sequences (200 cells) as input, and scATAC-seq signals on chromosome 15 in IMR-90, GM12878, and K562.*

Response to Reviewer #3

Reviewer #3 (Remarks to the Author):

In this manuscript, the authors present SUCCEED, a supervised multi-task DNA foundation model pretrained on 6,389 ENCODE tracks, combining a hybrid CNN-Transformer architecture to model both local sequence motifs and long-range regulatory dependencies. SUCCEED demonstrates competitive or superior performance across a range of genomic tasks, including epigenomic landscape prediction, chromatin accessibility denoising, and 3D chromatin architecture modeling. Notably, the model supports multi-resolution inputs, effective transfer learning, and generalization across unseen cell types.

Overall, this is a timely, well-executed, and impactful study. The authors convincingly argue that supervised foundation models — when trained on large-scale functional genomics datasets — can outperform self-supervised models that rely solely on masked language modeling. SUCCEED contributes a scalable and extensible framework that addresses key challenges in genome modeling, including task-specific retraining and underutilization of available epigenomic resources.

The suggestions below are intended to enhance the manuscript:

Major Comments

1. Data source diversity

The current pretraining exclusively uses data from the ENCODE database. While ENCODE offers high-quality and standardized datasets, it is not fully representative of all tissues, cell types, and biological contexts. The authors are encouraged to discuss whether incorporating additional data sources (e.g., Roadmap Epigenomics, FANTOM, or scENCODE) could further improve generalization.

<u>Response</u>:

We thank the reviewer for this insightful suggestion. We agree that while ENCODE provides high-quality and well-standardized epigenomic datasets, it does not fully cover the diversity of tissues, cell types, and experimental conditions observed in broader biological contexts.

In this work, we focused on ENCODE data to ensure consistency and quality control during pretraining, which is particularly important for large-scale foundation models. However, we fully acknowledge that incorporating additional epigenomic resources such as Roadmap Epigenomics, FANTOM, or emerging single-cell datasets represents a promising direction to further improve model generalization. To this end, we further fine-tuned the model on the Roadmap Epigenomics dataset and applied the fine-tuned model to the prediction of cell-type-specific epigenomic landscapes. The

results show that additional training consistently improves model performance, indicating that integrating more heterogeneous epigenomic data has strong potential to substantially enhance model generalization. However, it should be noted that data generated across different laboratories and sequencing platforms remain costly and time-consuming to collect, harmonize, and process, which constrains the feasibility of large-scale data integration.

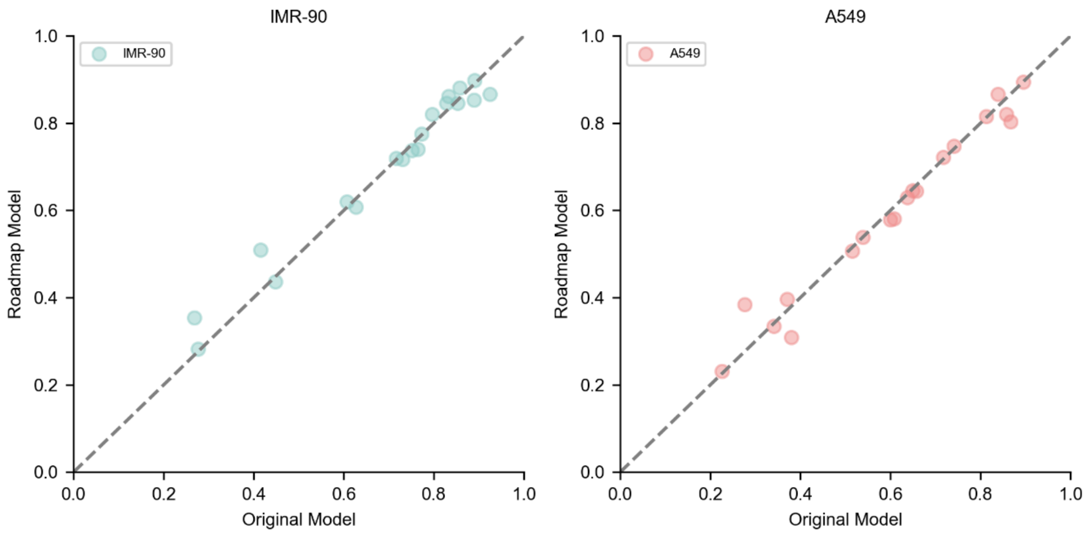

Fig. 1 for reviewer

We have added a discussion in the revised manuscript (Discussion) highlighting the potential benefits of multi-source pretraining, including improved robustness to domain shifts across tissues, protocols, and resolutions. Importantly, our model architecture and training pipeline are agnostic to data source and can naturally accommodate additional datasets in future work. We believe that extending pretraining to heterogeneous bulk and single-cell epigenomic datasets is an exciting avenue for future research, and we thank the reviewer for raising this important point.

To improve clarity, we have revised the corresponding parts of the manuscript.

Revised manuscript:

Page 15, Line 501: “*First, SUCCEED is currently trained in a supervised manner exclusively on human functional genomics data from the ENCODE consortium. Although ENCODE provides high-quality and well-standardized datasets, its coverage does not fully capture the diversity of tissues, cell types, and biological states present in vivo. Integrating additional functional genomics resources, such as Roadmap Epigenomics, FANTOM, and emerging single-cell epigenomic datasets, represents a promising direction for further improving model generalization, particularly in disease*

contexts, developmental stages, and rare cell types."

2. Handling of label noise and batch effects

As the model is trained in a supervised setting, label noise and batch effects from sequencing depth, protocol variations, and experimental conditions are inevitable. The authors should explicitly describe any strategies used to mitigate these issues (e.g., quality control thresholds, normalization, data augmentation, or loss weighting). This would be especially relevant in denoising tasks and across-cell-type transfer evaluations.

Response:

We thank the reviewer for raising this important point. We agree that label noise and batch effects are inherent challenges in supervised learning with functional genomics data, and that these issues are particularly relevant for denoising tasks and across-cell-type transfer evaluations. In this study, we address these challenges through careful data curation, standardized preprocessing, and controlled experimental design.

At the pretraining stage, multiple measures were taken to mitigate noise arising from sequencing depth variation, protocol differences, and experimental conditions. All input DNA sequences were extracted from the UCSC hg38 reference genome to ensure a consistent genomic coordinate system. Training targets were derived from human functional genomics data curated by ENCODE, including DNase-seq, ATAC-seq, transcription factor ChIP-seq, and histone modification ChIP-seq experiments. We retained only samples aligned to the hg38 assembly and excluded experiments annotated in ENCODE metadata as involving drug treatments, genetic perturbations, or other abnormal conditions, thereby reducing label noise introduced by non-baseline experimental settings. For experiments with multiple biological or technical replicates, replicate signals from the same experiment were merged prior to inclusion.

To further reduce batch effects across laboratories and experimental protocols, all epigenomic tracks were processed using ENCODE's standardized normalization and preprocessing pipelines. Specifically, ATAC-seq, transcription factor ChIP-seq, and histone ChIP-seq data used ENCODE-provided signal p-value bigWig files, while DNase-seq tracks used read-depth–normalized signal bigWig files. These standardized representations effectively reduced variability introduced by differences in sequencing depth and experimental procedures. In addition, all signals were clipped to the range of 0–32 to limit the influence of extreme values during model training. Although no explicit batch correction method was applied during training, the large-scale aggregation of 6,389 epigenomic tracks spanning diverse assay types, cell types, tissues, and laboratories encourages the model to learn robust, sequence-driven representations

rather than batch-specific artifacts. During pre-training, we employed data augmentation strategies that included reverse-complement transformation of input sequences and random shifts of 1-3 bp. Model optimization was performed using the AdamW optimizer implemented in PyTorch, with a learning rate of 1×10^{-4} and global gradient norm clipping at 0.2; no additional weighting was applied to the loss function.

For the denoising task, we explicitly modeled sequencing depth–induced noise by following the experimental design established by AtacWorks. Training data were derived from bulk ATAC-seq data from four cell types (B cells, natural killer (NK) cells, CD4+ T cells, and CD8+ T cells). In an additional data augmentation setting involving human early embryonic development data, raw BAM files were downsampled under a unified coordinate framework to simulate noisy or low-coverage conditions, while the corresponding samples with approximately 40 million reads were treated as high-quality clean targets. This controlled downsampling strategy ensures that the introduced noise primarily reflects sequencing depth variation rather than biological or batch-related confounders.

For single-cell chromatin accessibility denoising, we adopted the training and evaluation splits provided by AtacWorks, which include published human dscATAC-seq datasets from B cells, monocytes, and NK cells. For each cell type, 2,400 cells were aggregated to construct high-coverage reference signal tracks and peak calls. Noisy inputs were generated by randomly subsampling 1, 5, 10, or 50 cells, with repeated subsampling to increase data diversity and reduce sampling bias.

Importantly, across-cell-type transfer was explicitly evaluated by training models exclusively on B cell and monocyte data and testing them on NK cell data at matched coverage levels. This design enables a direct assessment of generalization under cell-type distribution shifts while minimizing confounding batch effects. In addition, further validation on PBMC data processed with SnapATAC2 from 10x Genomics fragment files supports the robustness of the model across different datasets and preprocessing pipelines.

Taken together, these data curation, normalization, and experimental design choices ensure robustness to label noise, sequencing depth variation, and batch effects at both the pretraining stage and during denoising and across-cell type transfer evaluations. We have revised the Methods section to explicitly clarify these strategies and their relevance to denoising performance and cross-cell-type generalization.

Revised manuscript:

Page 16, Line 546: "*To mitigate batch effects arising from differences in experimental protocols and laboratories, all tracks were processed using the standardized ENCODE*

normalization and preprocessing pipelines. Specifically, for ATAC-seq tracks, we used the Signal p-value bigWig files provided by ENCODE. The same Signal p-value bigWig files were used for transcription factor and histone modification ChIP-seq data, whereas DNase-seq tracks retained read-depth–normalized signal bigWig files."

Page 23, Line 811: "*For the ATAC-seq inputs, we obtained aligned BAM files for each cell type from ENCODE. We then generated bigWig tracks using bamCoverage with RPGC normalization, and extracted the ATAC-seq signal for each contig to construct the model inputs. The labels used during the model training process are the bigWig files of Signal P-values after standardized processing provided by ENCODE.*"

Page 25, Line 874: "*The data from the Erythroblasts cell line and early human embryonic development also followed the aforementioned processing method but were not involved in the model training process.*"

Page 27, Line 928: "*The datasets used for benchmarking against C.Origami were obtained from the publicly available resources released in its original publication and include Hi-C, ATAC-seq, and CTCF ChIP-seq data for the IMR-90 and GM12878 cell lines. For cross-species evaluation and zero-shot transfer experiments, Hi-C data were used after ICE normalization without additional preprocessing. For the ATAC-seq inputs, CPM-normalized signals were generated using bamCoverage, which helps mitigate the effects of sequencing depth variability and batch effects.*"

3. Mechanistic architecture rationale

While the manuscript describes the use of convolutional layers for motif detection and Transformers for long-range interactions, a deeper explanation of how these components interact — including the rationale for architectural choices like rotary embeddings, grouped query attention, and their biological relevance — would strengthen the understanding of the model's design.

<u>Response</u>:

We thank the reviewer for this suggestion. We agree that a clearer explanation of the interaction between convolutional layers and Transformer layers would improve the understanding of the model design. Below, we clarify the respective roles of these components, and we also explicitly distinguish architectural choices motivated by biological considerations from those motivated primarily by computational efficiency.

In our model, convolutional layers and Transformer layers play complementary roles. The convolutional layers serve as local feature extractors that capture short sequence patterns, such as putative transcription factor binding motifs or other cis-regulatory elements. These locally extracted features are then passed to Transformer layers, which model long-range dependencies and interactions among sequence elements across large genomic distances. This hierarchical design is consistent with the

biological organization of gene regulation, in which local regulatory elements interact combinatorially over long ranges.

We adopt rotary positional embeddings in the Transformer layers to encode relative positional information within the attention mechanism. Compared to absolute positional encodings, rotary embeddings facilitate distance-aware attention and are well-suited for modeling interactions whose functional relevance depends on relative spacing between sequence elements, a common characteristic of regulatory genomics. Grouped query attention was adopted primarily for architectural consistency with recent large-scale Transformer models and for its favorable memory properties with respect to key–value representations; it has a limited impact on the core attention computation and does not encode a specific biological assumption.

In addition, we performed a systematic interpretability analysis of the convolutional and Transformer layers. The results show that the convolutional layers capture sequence motifs associated with biological function, whereas the Transformer layers effectively exploit long-range genomic interactions during modeling, indicating that the model integrates regulatory signals across genomic scales.

We have revised the Results and Methods section to more clearly explain the interaction between convolutional and Transformer components, and to explicitly state the motivation behind individual architectural choices.

Revised manuscript:

Page 3, Line 73: "*Specifically, the architecture consists of four main components: a convolutional frontend for early motif extraction, a multi-stage downsampling convolutional tower, a Transformer encoder for modeling long-range dependencies, and a pointwise prediction head with multitask outputs (Methods). A one-dimensional convolutional neural network identifies conserved local sequence motifs within cis-regulatory elements, such as promoters and enhancers. The downsampling tower enables the model to reduce spatial resolution progressively, allowing it to integrate information from different genomic regions effectively. These local features are passed to a Transformer module, which captures long-range dependencies by modeling interactions between distal genomic regions. Finally, a pointwise multi-task output head is used to support concurrent prediction of multiple regulatory signals, including chromatin accessibility, enhancer activity, and transcription factor binding.*"

Page 3, Line 89: "*In addition, motivated by recent advances in large language models, we systematically optimized key components of our architecture[12, 26, 27]. We incorporated the SwiGLU activation into the Transformer modules, where its gating mechanism adaptively modulates information flow, substantially enhancing*

representational flexibility and expressive capacity compared with piecewise-linear activations such as ReLU[28]. In parallel, we replaced conventional LayerNorm with RMSNorm. By normalizing activations solely based on their root mean square, RMSNorm eliminates mean subtraction, reduces computational overhead, and preserves the directional information of representations, thereby improving training stability in large-scale Transformer models[29]. Furthermore, we introduced rotary positional embeddings to jointly encode the relative and absolute positional information of regulatory elements within sequences, strengthening the model's ability to capture and exploit sequence structure[30]."

Newly added figure:

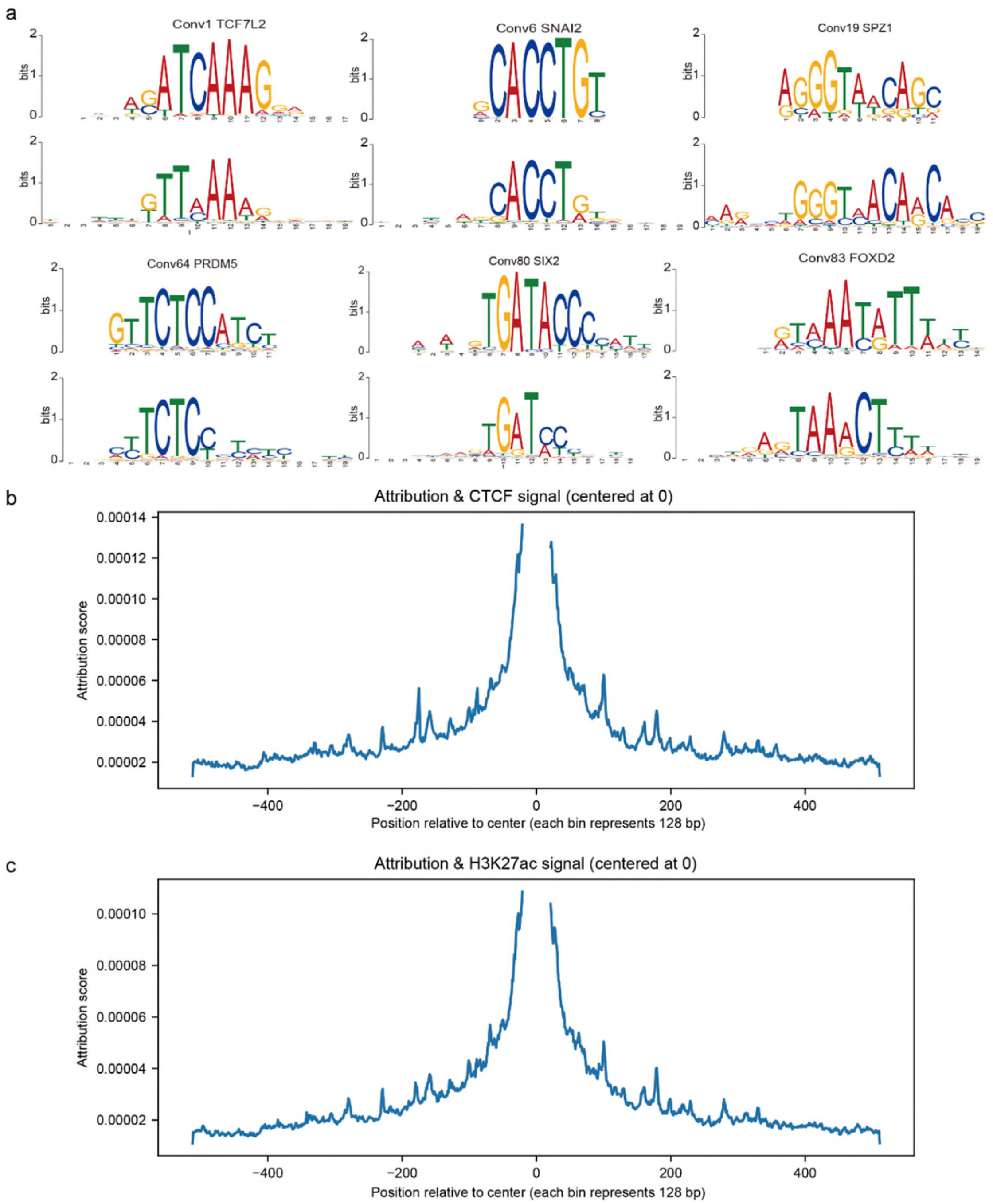

Supplementary Fig. 3 | Interpretability analysis of the SUCCEED pre-trained model. ***a,*** *Sequence motifs learned by the first convolutional layer of the SUCCEED model.* ***b,*** *Attribution scores computed using the Input × Gradient method for the CTCF ChIP-seq signal prediction task, aggregated across all samples; the x-axis denotes relative genomic positions.* ***c,*** *Attribution scores computed using the Input × Gradient method for the H3K27ac ChIP-seq signal prediction task, aggregated across all samples; the x-axis denotes relative genomic positions.*

4. Cross-species transferability

All experiments are conducted on the human genome (hg38). Since several recent genomic foundation models demonstrate potential for cross-species generalization, it would be valuable to include experiments (or at least discussion) on SUCCEED's performance when applied to non-human genomes (e.g., mouse, zebrafish). This would enhance the model's relevance for comparative genomics and evolutionary studies.

Response:

We thank the reviewer for this valuable suggestion. In response, we have conducted explicit cross-species generalization analyses using mouse genomic data to evaluate SUCCEED's transferability beyond the human genome.

Specifically, we applied models trained exclusively on human cell lines to multiple mouse genomics tasks in a zero-shot setting. These include (1) cell-type–specific epigenomic prediction in mouse (Supplementary Fig. 7), (2) denoising and enhancement of chromatin accessibility data during early mouse embryonic development (Supplementary Fig. 10), and (3) prediction of three-dimensional chromatin architecture across diverse mouse tissues and cell types using only mouse chromatin accessibility data (Supplementary Fig. 14). Importantly, no mouse data were used during training in any of these experiments.

We find that, despite the absence of species-specific training, the pretrained models generalize effectively to mouse genomic data across all evaluated tasks. These results indicate that SUCCEED captures conserved regulatory sequence features shared across species and retains strong predictive power when applied to non-human genomes. We have incorporated these cross-species analyses into the revised manuscript and expanded the Results and Discussion to highlight the implications for comparative genomics and evolutionary studies.

Revised manuscript:

Page 9, Line 297: "*SUCCEED was pre-trained and fine-tuned exclusively on large-scale human functional genomics data. To assess whether the learned prior knowledge generalizes across species, we performed zero-shot transfer evaluations in multiple mouse tissues, including heart, liver, kidney and lung. Despite having no exposure to mouse DNA sequences or functional genomics data, SUCCEED achieved high accuracy across several epigenomic signal prediction tasks, particularly for CTCF and H3K27ac (Supplementary Fig. 7 and Supplementary Table 5). These results indicate that key aspects of genomic regulatory grammar are functionally conserved between human and mouse. Overall, these results demonstrate that SUCCEED effectively leverages regulatory grammar priors learned from large-scale external datasets to accurately predict cell-type-specific epigenomic landscapes and to*

generalize across species in a zero-shot manner. This capability highlights its potential utility for studying transcriptional regulation in rare cell types and model organisms."

Page 11, Line 381: "*To evaluate the zero-shot cross-species transferability of SUCCEED in denoising and signal enhancement, we applied the model to ATAC-seq data from early mouse embryonic development. Despite having no prior exposure to mouse data, SUCCEED effectively denoised and enhanced chromatin accessibility signals (Supplementary Fig. 11a). Notably, even under low-coverage conditions, the enhanced signals remained highly correlated with the original sequencing data (Supplementary Fig. 11b). Collectively, these results demonstrate that SUCCEED leverages regulatory grammar priors to robustly denoise and enhance chromatin accessibility across bulk, single-cell, and low-coverage ATAC-seq data, while generalizing across species, thereby providing a unified and scalable framework for high-resolution epigenomic profiling.*"

Page 11, Line 454: "*We further evaluated SUCCEED's ability to predict three-dimensional chromatin architecture across species in a zero-shot setting (Supplementary Fig. 15). Specifically, we applied SUCCEED, trained exclusively on human data, to mouse early embryonic stages (8-cell and mESC) and the mouse cell line Patski. The results show that, using only ATAC-seq signals together with learned sequence representations, the predicted Hi-C maps exhibit high concordance with experimentally measured data in terms of insulation scores, suggesting a degree of structural conservation in topologically associating domain (TAD) formation between human and mouse (Supplementary Fig. 15a). In contrast, the correlation between predicted and experimental Hi-C data at the observed/expected level is lower than that achieved on human datasets, indicating the presence of species-specific differences in local chromatin architecture. We performed a visual comparison between the predicted Hi-C maps and experimentally measured Hi-C data on mouse chromosome 3. The results show that, under a zero-shot setting, the model can accurately reconstruct three-dimensional chromatin organization across different mouse cell types and tissues using only ATAC-seq signals, demonstrating robust cross-species generalization (Supplementary Fig. 15b).*"

Newly added figure:

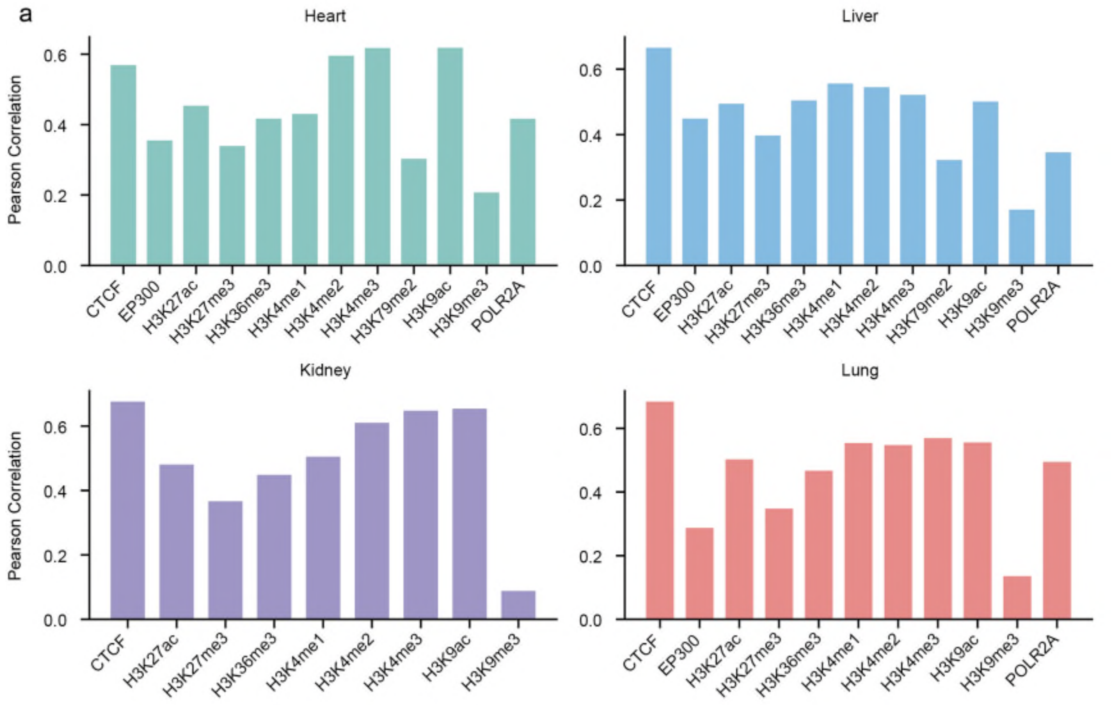

Supplementary Fig. 7 | Cross-species zero-shot prediction of mouse epigenomic landscapes by SUCCEED. a, *Pearson correlation coefficients between epigenomic signals predicted by SUCCEED in a zero-shot manner and experimentally measured epigenomic signals across four mouse tissues (heart, liver, kidney and lung).*

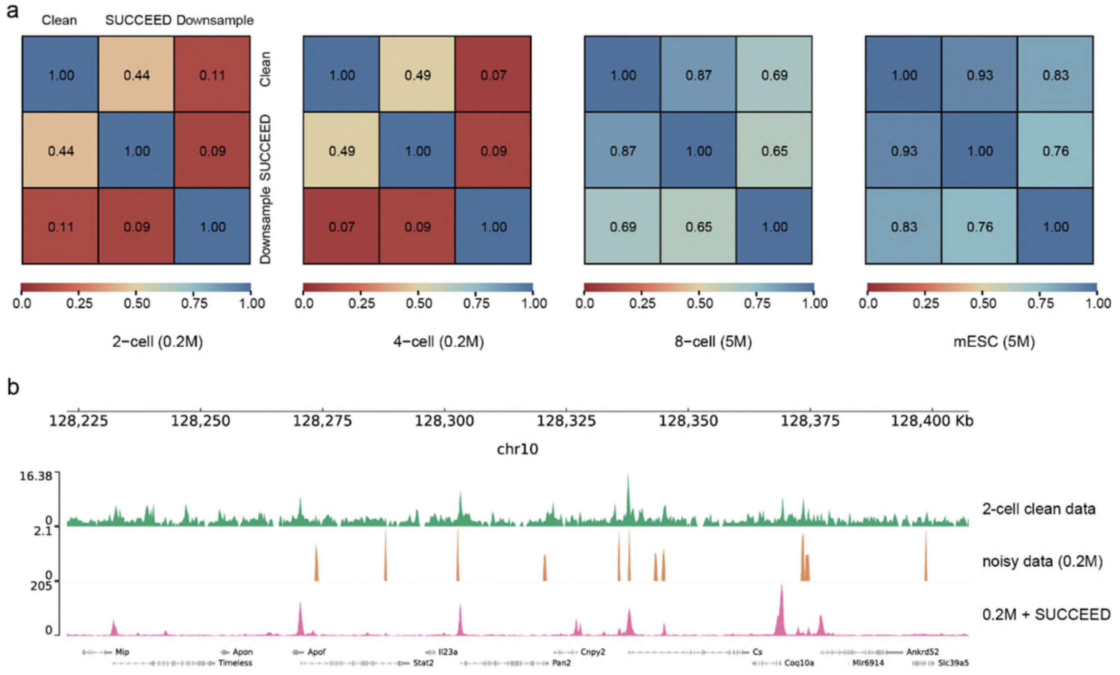

Supplementary Fig. 11 | Cross-species zero-shot denoising and enhancement of

ATAC-seq data by SUCCEED. a, *Comparison of denoising and enhancement performance of SUCCEED during early mouse embryonic development across four stages (2-cell, 4-cell, 8-cell and mESC). Sequencing depths of 0.2M and 5M correspond to 200,000 and 5,000,000 reads, respectively.* ***b,*** *Comparison of chromatin accessibility signals after SUCCEED-based enhancement with low-coverage input signals and the original signals.*

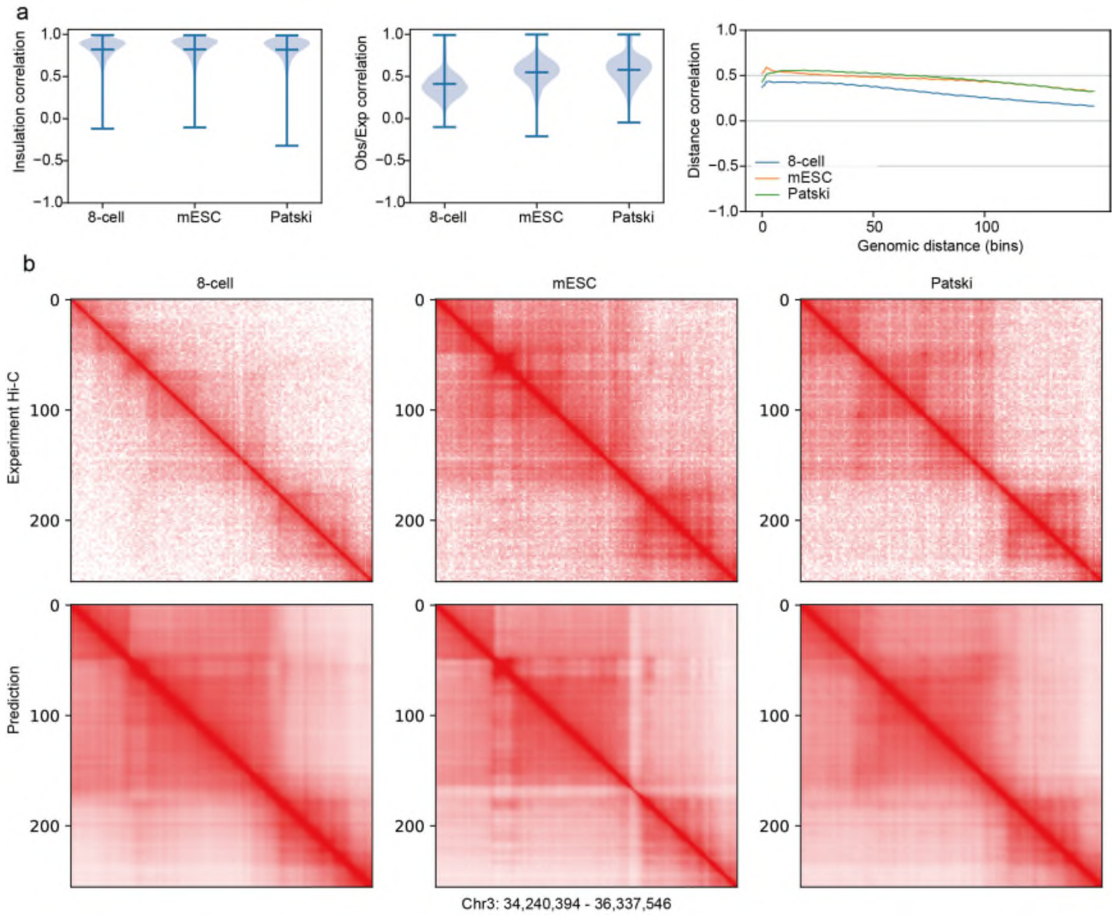

Supplementary Fig. 15 | Cross-species zero-shot prediction of 3D chromatin organization in mouse tissues and cell lines by SUCCEED.a, *Pearson correlation coefficients between predicted and experimentally measured Hi-C contact maps across different mouse tissues and cell lines (8-cell, mESC and Patski). From left to right, correlations are shown for insulation scores, observed/expected (O/E) contact frequencies and distance-stratified contact frequencies. Hi-C contact maps were predicted by SUCCEED from DNA sequence and ATAC-seq signals.* ***b,*** *Representative visualization of predicted and experimentally measured Hi-C contact maps on chromosome 3 (chr3) across different mouse tissues and cell lines.*

5. Biological interpretation of multi-resolution modeling

The model supports various input lengths and resolutions (e.g., 131k-128bp to 2M-2048bp). However, the manuscript lacks a biological interpretation of how these scales map to genomic features (e.g., nucleosomes, topologically associating domains, enhancers). A brief discussion linking resolution to biological phenomena would clarify the motivation for supporting multiple resolutions.

Response:

We thank the reviewer for this insightful comment. We agree that clarifying the biological motivation behind supporting multiple input lengths and resolutions would improve the interpretability of the model design. Our choice of scales is motivated by established practices in genomic sequence modeling and by the hierarchical organization of genome regulation, rather than by a strict one-to-one mapping between resolution and a single biological structure.

The supported input configurations (131,072 bp at 128 bp resolution; 524,288 bp at 512 bp resolution; 1,048,576 bp at 1,024 bp resolution; and 2,097,152 bp at 8,192 bp resolution) are chosen to span a range of genomic contexts commonly studied in functional and structural genomics. These scales align with resolutions that have been widely adopted in prior work. For example, Basenji2 models functional genomics signals using 131,072 bp inputs at 128 bp resolution, Borzoi extends the input length to 524,288 bp to capture broader regulatory context, Akita models Hi-C contact maps at megabase-scale input lengths (1,048,576 bp), and C. Origami further explores multi-megabase contexts at coarser resolutions (2,097,152 bp at 8,192 bp resolution).

From a biological perspective, finer resolutions (e.g., 128 bp) are well-suited for modeling local regulatory signals such as transcription factor binding and chromatin accessibility, which operate at the scale of individual motifs or nucleosome-sized regions. Intermediate resolutions (e.g., 512–1,024 bp) capture interactions among nearby regulatory elements, such as enhancers and promoters, across tens to hundreds of kilobases. Coarser resolutions paired with larger input lengths enable the model to integrate long-range regulatory context and structural features, including domain-scale chromatin organization and higher-order interactions that extend over hundreds of kilobases to megabases.

Importantly, we do not assume a strict correspondence between any single resolution and a specific biological entity (e.g., a nucleosome or a topologically associating domain). Instead, supporting multiple input lengths and resolutions allows the model to flexibly operate across different levels of genomic organization, consistent with the multi-scale nature of gene regulation. We have added specific details in the revised draft to clarify this motivation.

<u>Revised manuscript:</u>

Page 6, Line 178: *“From a biological perspective, finer resolutions (for example, 128 bp) are well suited for modeling local regulatory signals such as transcription factor binding and chromatin accessibility, which operate at the scale of individual motifs or nucleosome-sized regions. Intermediate resolutions (for example, 512-1,024 bp) capture interactions among nearby regulatory elements, including enhancers and promoters, across tens to hundreds of kilobases. In contrast, coarser resolutions paired with larger input lengths enable the integration of long-range regulatory context and structural features, such as domain-scale chromatin organization and higher-order interactions spanning hundreds of kilobases to megabases.”*

Minor Comments

1. Methodological details

The Methods section, while comprehensive, would benefit from clearer structure and concise descriptions of:

(1) Data partitioning strategies (e.g., how training/test chromosomes were selected)

(2) Specific hyperparameters (e.g., batch size, learning rate, optimizer)

(3) Early stopping criteria and fine-tuning protocols (especially in multi-task and cross-cell-type settings)

<u>Response</u>:

We thank the reviewer for this comment. We have revised the Methods accordingly and clarified the requested points below.

(1) Data partitioning.

During model pretraining, we followed the data partitioning strategy used in Enformer and Basenji2, in which the genome is segmented into fixed-resolution samples and split into training, validation, and test sets using an 8:1:1 ratio. For all downstream experiments, genomic regions were partitioned at the chromosome level to avoid information leakage between training and evaluation sets. Specifically, a fixed subset of chromosomes was held out for validation and testing, while the remaining chromosomes were used for training. The revised Methods section now explicitly lists the chromosome assignments used for training, validation, and testing in each setting.

(2) Hyperparameters.

We now provide a consolidated description of the key hyperparameters used in training, including batch size, optimizer, learning rate, learning rate schedule, and weight decay (Supplementary Table 2). Unless otherwise noted, the same set of hyperparameters was

used across experiments to ensure comparability. Any task-specific deviations are explicitly stated in the corresponding subsections.

(3) Early stopping and fine-tuning protocols.

To prevent overfitting, we applied early stopping across all tasks, with termination based on validation loss. During SUCCEED pretraining, a patience of eight epochs was used, such that training was stopped if validation performance failed to improve for eight consecutive epochs. For downstream tasks, the patience was set to five epochs. Fine-tuning strategies varied across tasks and are described in detail in the Methods.

Specifically, for sequence-only transfer tasks, we evaluated both full fine-tuning and partial fine-tuning settings. Full fine-tuning involved initializing the model with pretrained weights and updating all parameters on the target task, whereas partial fine-tuning updated only the classification layer while freezing the remaining parameters. For cell-type-specific epigenomic profile prediction and chromatin accessibility denoising and enhancement, all pretrained model parameters were frozen, and only the ATAC-seq signal encoder and joint representation decoder were trained. For cell-type-specific three-dimensional chromatin structure prediction, we evaluated both full fine-tuning and parameter-freezing strategies.

Revised manuscript:

Page 20, Line 677: "*We trained, evaluated, and tested the model on the same target datasets across all experiments, using the Poisson negative log-likelihood loss function adopted in Enformer and Basenji2. During model pretraining, we followed the data partitioning strategy used in Enformer and Basenji2, in which the genome is segmented into fixed-resolution samples and split into training, validation, and test sets using an 8:1:1 ratio. Two data augmentation strategies were applied during training: reverse-complementing the input sequences and introducing a random 1-3 bp shift. Optimization was performed using the AdamW algorithm in PyTorch with a learning rate of 1×10^{-4}, and gradients were clipped to a maximum global norm of 0.2. Early stopping was employed with validation loss as the monitoring metric and a patience of 8 epochs (Supplementary Table 3).*"

Page 21, Line 710: "*We conducted systematic benchmarks of SUCCEED against HyenaDNA, DNABERT, DNABERT2, and NucleotideTransformer on classical promoter-prediction and splice-site prediction tasks. The promoter benchmarks follow the six tasks provided in GenBench—promoter prediction, no-TATA promoter prediction, TATA promoter prediction, core promoter prediction, no-TATA core promoter prediction, and TATA core promoter prediction—all formulated as binary classification. In addition, we evaluated a human splice-site prediction task from GenBench, defined as a multi-*

task classification problem. All experiments used the training and test splits predefined in GenBench."

Page 24, Line 838: "*The EPCOT model weights used for benchmarking were obtained from the official repository (https://github.com/liu-bioinfo-lab/EPCOT), and the input datasets were downloaded from the sources referenced therein (Supplementary Table 4). All models were evaluated on an identical held-out test set, with chromosome 2 designated for validation, chromosomes 10 and 21 for testing, and all remaining chromosomes for training. Cross-chromosomal evaluations were performed on chromosomes 10 and 20 in the K562, GM12878, HepG2, and MCF-7 cell lines, and cross-cell-type evaluations were conducted in IMR-90, A549, and H1. Model performance was quantified using the Pearson correlation coefficient between predicted and observed signals.*"

2. Clarification in Figure 1

In the diagram summarizing model capabilities (Fig. 1), the explanation of "unseen datasets" is somewhat ambiguous. Please clarify whether it refers to unseen cell types, data modalities, or genomic regions, and how transfer learning is achieved in each case.

Response:

We thank the reviewer for this comment. We agree that the term "unseen datasets" in Fig. 1 could be interpreted ambiguously and requires clarification.

In this work, "unseen datasets" primarily refers to data from previously unseen cell types and experimental datasets that were not used during model training. In the context of different tasks, this includes (i) cell types held out during training and used only for evaluation (e.g., in across-cell-type transfer experiments), and (ii) independent datasets generated using different experimental protocols or preprocessing pipelines. It does not refer to unseen genomic regions, as all evaluations are performed on genomic regions held out at the chromosome level following standard train-validation-test splits.

Transfer learning in these settings is achieved by pretraining the model on large-scale functional genomics data and then directly applying or fine-tuning the pretrained model on the target dataset, depending on the task. The same underlying architecture is used across settings, with task-specific objectives and, when applicable, fine-tuning on data from the target domain.

We have revised the descriptions in Fig. 1 and the Methods section to explicitly clarify the definition of "unseen datasets" and to distinguish between unseen cell types and unseen experimental datasets, thereby improving the clarity of the manuscript.

Revised manuscript:

Page 22, Line 775: "*The model is transferred to epigenomic profiles from previously unseen cell types or tissues. Single-cell ATAC-seq (scATAC-seq) data for multiple human brain cell types were obtained from the CATlas database (https://decoder-genetics.wustl.edu/catlasv1/catlas_humanbrain). Pseudo-bulk profiles were generated based on cell type annotations, resulting in bigWig files for 128 distinct cell types. Following the preprocessing pipeline, signal values were extracted from the corresponding bigWig files to construct model inputs at 131k-128bp resolution.*"

3. Error bars in Figure 2b

Some histone modification bar plots in Figure 2b appear to lack error bars, while others include them. Please ensure consistency and clarify whether the error bars represent standard deviation or standard error across features/tasks.

Response:

We thank the reviewer for this comment and agree that error bars should be clearly defined and consistently interpreted.

In Figure 2b, error bars represent the standard deviation across features or tracks within each group. Error bars are shown only when multiple measurements are available for a given histone modification and condition. In cases where only a single measurement is available (i.e., no replicate features or tasks), the standard deviation cannot be computed and therefore no error bar is displayed.

To further clarify this point, we provide a summary of the number of features/tracks associated with each histone modification, which explains why error bars are present for some modifications but absent for others. We have revised the figure caption to explicitly state that the error bars denote standard deviation and to explain why some bars do not display error bars, ensuring a consistent interpretation of the results.

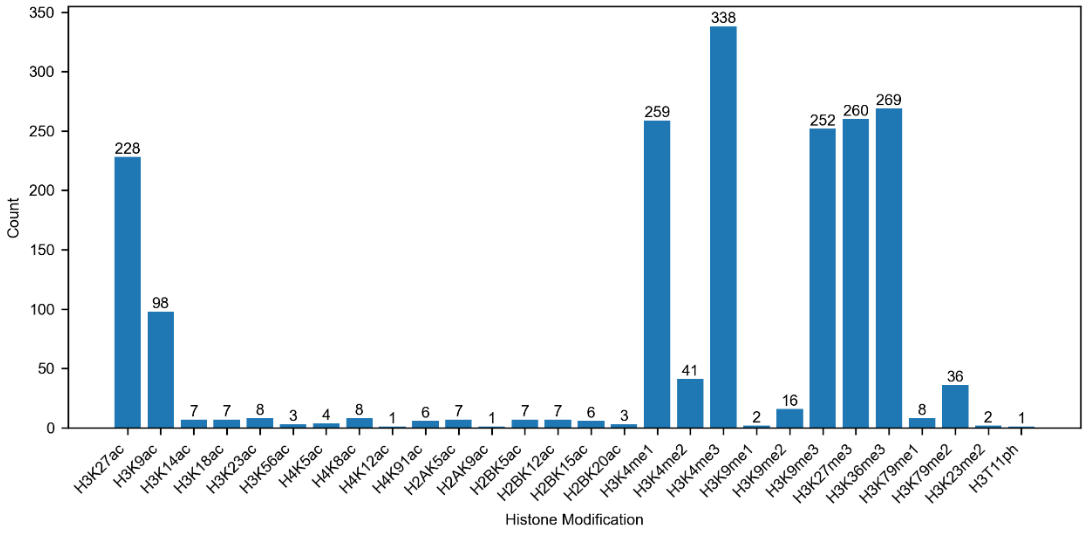

Fig. 2 for reviewer

Revised figure:

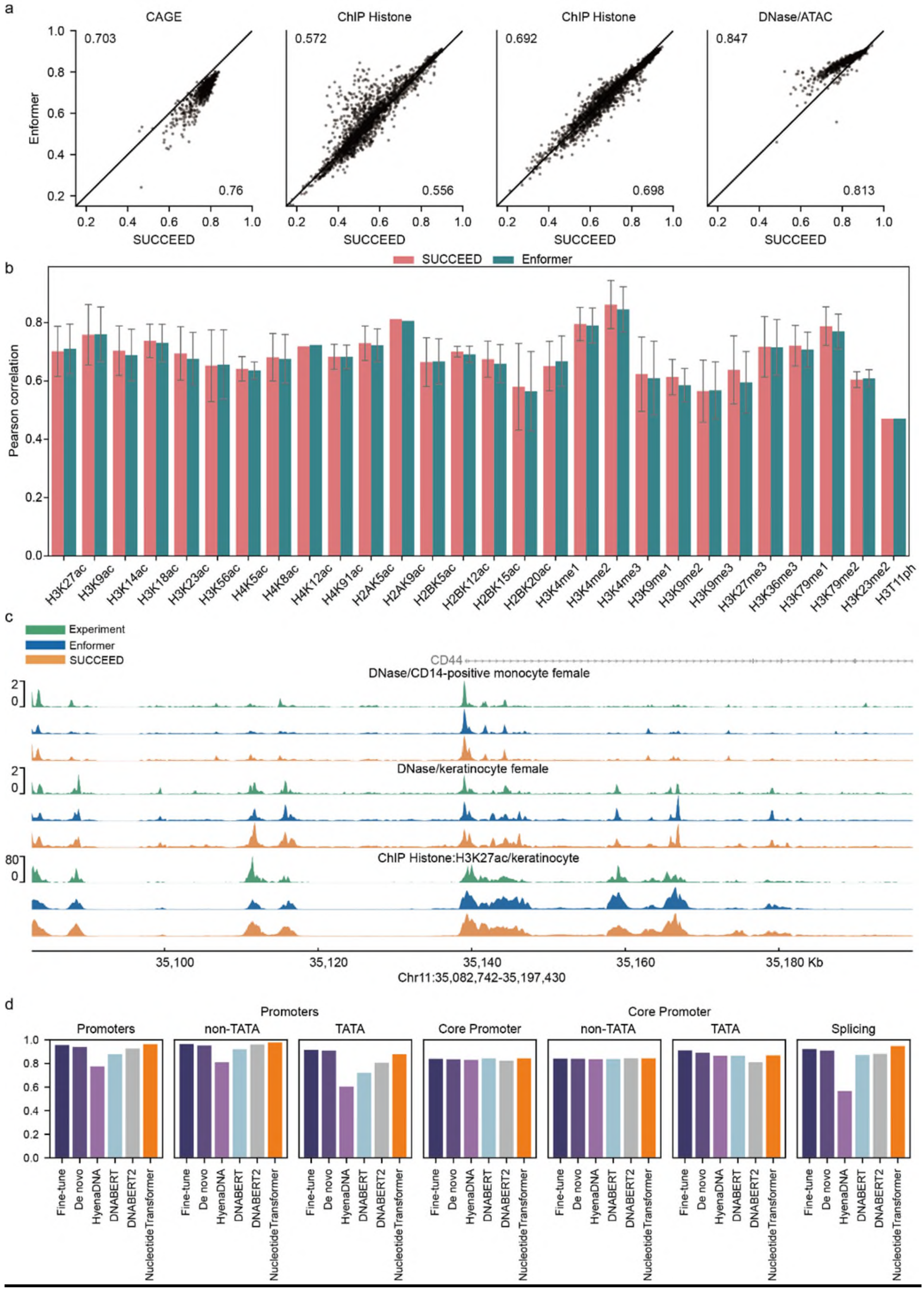

Fig. 2 | Model performance evaluation. ***a***, *Comparison of SUCCEED and Enformer results across 638 CAGE, 2131 transcription factor (TF) ChIP-seq, 1860 histone modification ChIP-seq, and 684 DNase-seq or ATAC-seq datasets.* ***b***, *Pearson correlation coefficients between SUCCEED and Enformer for various histone modification datasets. The x-axis represents specific histone modifications, while the y-*

axis shows the average Pearson correlation coefficient, with error bars representing the standard deviation (s.d.) of the results. ***c***, *Representative examples of predicted genomic trajectories at the CD44 locus in the test set, illustrating the consistency between SUCCEED and Enformer predictions and the true results. The three trajectories in each experiment share the same y-axis. d, SUCCEED was benchmarked against masked self-supervised genomic language models across seven representative genomic tasks, with de novo referring to models trained from scratch on a given task without leveraging pretrained parameters.*

4. Axis labels in Figure 3c and 3d

The terms "cell type" and "region" in the scatter plots require clarification. Do they refer to specific cell populations and genomic loci, respectively? Please define these labels either in the figure caption or main text for interpretability.

Response:

We thank the reviewer for this helpful comment and agree that the terms "cell type" and "region" require a clearer definition for interpretability. In Figures 3c and 3d, these labels do not denote individual cell populations or specific genomic loci per se, but rather represent two complementary dimensions along which predictive performance is summarized.

Specifically, for each cell type and each test genomic interval of 131,072 bp, we computed the Pearson correlation between the predicted and observed chromatin accessibility signals within that interval. Performance summarized by cell type was obtained by aggregating these correlations across all test intervals for a given cell type, whereas performance summarized by region was obtained by aggregating correlations across cell types for a given genomic interval.

We have revised the figure captions of Figures 3c and 3d to explicitly define these terms and clarify their meanings, and added more detailed descriptions in the methods section to enhance the interpretability of the figures.

Revised manuscript:

Page 23, Line 782: "*We trained two models: one initialized from scratch on the human brain scATAC-seq dataset, and another obtained by fully fine-tuning the 131k-128bp model pretrained on the broader ENCODE dataset. Both models were evaluated on the same independent test set using identical hyperparameter configurations, and performance was quantified using the Pearson correlation between predicted and observed signals.*"

Page 23, Line 787: "*We evaluated the predictive performance of the model for chromatin accessibility along two dimensions: cell type and genomic region.*

Specifically, for each cell type and each test genomic interval of 131,072 bp, we aligned the predicted chromatin accessibility signal with the corresponding observed signal at matched genomic positions and computed the Pearson correlation coefficient between them. Performance summarized by cell type was obtained by aggregating correlation coefficients across all test intervals for a given cell type, thereby quantifying overall predictive concordance within that cell type. Conversely, performance summarized by region was obtained by aggregating correlation coefficients across cell types for a given genomic interval, reflecting variation in predictive difficulty and robustness across different genomic regions."

Conclusion

This work presents a significant step forward in the development of scalable and biologically informed genomic foundation models. With some clarifications and improved methodological transparency, the paper is well-positioned for publication in Nature Communications.

Response to Reviewer #1

Reviewer #1 (Remarks to the Author):

This second version has been substantially improved. Method descriptions have been expanded and the manuscript now includes some rational about architectural choices as well as assessments of key neural-network components. Most importantly, the authors have included comparisons against several other well known foundation models, an aspect that was absent from the first manuscript.

This last point is particularly important. Dozen foundation models have been already proposed in last years, so these comparisons should be a central point for any new approach. As such, I do not think that they are sufficiently highlighted in the new manuscript and I would recommend rewriting certain parts to highlight them better:

1/ first, rather than being exclusively in supp. figures, these results should appear (at least partly) in the main figures 4, 5 & 6.

Response: We thank the reviewer for this helpful suggestion. Following the reviewer's advice, we have revised the manuscript to better highlight the comparisons with existing foundation models. Specifically, key results that were previously presented only in the Supplementary Figures have now been incorporated into the main text and are shown in Figures 4–6, where they are most relevant to the corresponding analyses. This change makes the comparisons more visible and emphasizes the performance of our method relative to other foundation models.

2/ I would recommend to rewrite some parts that present the performances of Succeed in a way that is a little bit biased. Notably:

- l.146-184: it is difficult to say that Succeed "outperforms" the models trained from scratch as both models achieve almost identical accuracy (I doubt the difference is even statistically significant).

- l.287-289: Succeed seems to perform slightly better solely on cross-chromosome comparisons, which are not of prime interest here. The goal of the task is to "characterize epigenomic profiles in unseen cell types" (l.223-224), which corresponds to the cross-cell-type comparison where Sei and Succeed have very similar accuracy.

- l.446-449: We cannot really say "The results show that SUCCEED achieves the best performance among all methods". Succeed seems to perform slightly better than Sei in bulk ATAC-seq (supp. fig. 13) but slightly worse in single cell (supp. fig. 14). It is also misleading to present the results achieved on chromosome 10 and 15 on the same level (see also Fig. 6). Chromosome 10 has been used for validation purpose, so the

measured accuracies tend to be optimistically biased. Hence the only valid comparisons should be done on chromosome 15.

Response: We thank the reviewer for this detailed and constructive comment. We agree that the descriptions of SUCCEED's performance in the previous version could give an overly strong impression. We have therefore revised the corresponding sections to provide a more balanced and precise description of the results.

Lines 147–150. We have removed the wording suggesting that SUCCEED "outperforms" models trained from scratch. The text has been revised to state that the two approaches achieve comparable accuracy, without implying statistically significant differences.

Lines 284–289. We agree that the primary objective of this task is to characterize epigenomic profiles in unseen cell types. Accordingly, we have revised this section to focus on the cross-cell-type evaluation, where SUCCEED and Sei show very similar accuracy, and we no longer emphasize cross-chromosome comparisons as evidence of superior performance.

Lines 451–458. We have revised this section to avoid stating that SUCCEED achieves the best performance among all methods. The updated text now distinguishes between the bulk and single-cell ATAC-seq evaluations, indicating that SUCCEED performs slightly better in the bulk ATAC-seq setting, whereas Sei shows comparable or slightly better performance in the single-cell setting. In addition, we now clarify that chromosome 10 was used for validation during model development and may therefore lead to optimistic estimates. As suggested by the reviewer, we emphasize comparisons on chromosome 15 as the primary evaluation.

Revised manuscript:

Page 5, Line 147: "*We compared SUCCEED with DNA foundation models on these tasks and found that it achieves performance comparable to, or exceeding, that of larger self-supervised models across most genomic benchmarks.*"

Page 9, Line 284: "*We observed that SUCCEED and Sei, which are both pretrained on large-scale functional genomics data, consistently achieve higher performance than self-supervised sequence-only models when predicting cell-type-specific epigenomic signals (Supplementary Fig. 6f, g, h). This advantage remains robust across cross-chromosomal, cross-cell type, and the more stringent combined cross-chromosomal and cross-cell type evaluation settings (Supplementary Fig. 6f, g, h).*"

Page 14, Line 451: "*When bulk ATAC-seq is used as input, SUCCEED attains slightly higher accuracy. In contrast, under single-cell ATAC-seq input, Sei—pretrained on large-scale functional genomics data—shows comparable or marginally superior*

performance (Supplementary Fig. 13 and Supplementary Fig. 14). Results are reported for both chromosome 10 and chromosome 15; however, because chromosome 10 was used during validation, the evaluation on chromosome 15 provides a less biased assessment of generalization performance."

3/ I would also recommend to entirely rewrite the Discussion to discuss the performance of the other foundations models. Overall, Succeed has very similar performances as Sei, sometimes slightly better for certain tasks, and slightly worse for others. Additionally, given their very similar performance, Succeed and Sei should also be compared in terms of model complexity (number of variables) and computation time for learning.

Response: We thank the reviewer for this constructive suggestion. Following this advice, we have revised the Discussion section to more explicitly compare SUCCEED with other foundation models, particularly Sei, across the evaluated tasks (Page 16, Lines 505–510 and Lines 528–536).

The revised discussion now emphasizes that SUCCEED and Sei achieve broadly comparable performance across several benchmarks, with SUCCEED performing slightly better in some settings and Sei performing similarly or marginally better in others. We also expanded the discussion to place these results in the broader context of existing DNA foundation models. In addition, as suggested by the reviewer, we now discuss model characteristics such as architectural design and computational considerations, highlighting potential trade-offs between predictive performance, model complexity, and efficiency.

Revised manuscript:

Page 16, Line 505: "*Further comparisons indicate that SUCCEED and Sei, both pretrained in a supervised manner on large-scale functional genomics data, generally outperform self-supervised models trained solely on DNA sequence across multiple application scenarios. These observations are consistent with recent findings by Tang et al., who similarly reported advantages of supervised pretraining over self-supervised sequence-only approaches*[59]*.*"

Page 16, Line 528: "*In our comparisons, Sei—trained in a supervised manner on large-scale functional genomics data—achieves competitive performance using a purely convolutional architecture. SUCCEED, in contrast, adopts a hybrid convolution–Transformer architecture designed to capture long-range regulatory interactions. While this design enables SUCCEED to model distal genomic dependencies, it also introduces additional computational cost compared with convolution-only models. Extending convolutional architectures with efficient*

mechanisms for modeling long-range dependencies may therefore further improve the balance between predictive performance and computational efficiency."

4/ The abstract should also be rewritten to include these comparisons and conclusions.

Response: We thank the reviewer for this suggestion. The abstract has been revised to explicitly include the relevant performance comparisons and key conclusions. Specifically, we now summarize SUCCEED's performance relative to existing domain-specific models, its comparison with Sei, and its advantages over self-supervised DNA models. The revised abstract also clarifies the broader implications of these findings.

5/ Given the results achieved by Succeed and the other foundation models an important recent reference is missing IMO. The paper of Tang et al. 2025 (Genome Biology) reaches very similar conclusions as this paper (supervised models are better than self-supervised models). This should also be discussed in the Discussion.

Response: We thank the reviewer for pointing out this important reference. We have now included Tang et al. (2025, *Genome Biology*) in the revised manuscript and incorporated a discussion of its findings in the Discussion section (Page 16, Lines 505–510). We note that their conclusions regarding the comparative advantages of supervised over self-supervised models are consistent with our observations, and we now explicitly discuss this alignment.

Newly added Reference:

59. *Tang Z, Somia N, Yu Y, Koo PK. Evaluating the representational power of pre-trained DNA language models for regulatory genomics. Genome Biology* ***26****, 203 (2025).*

Response to Reviewer #2

Reviewer #2 (Remarks to the Author):
The research method of this work is advanced, and the research content is of great significance. All the issues I am concerned about have been resolved, and I agree to accept this manuscript.

Response: We thank the reviewer for the positive assessment of our work and for the constructive comments provided throughout the review process. We are pleased that the concerns have been satisfactorily addressed.

Reviewer #2 (Remarks on code availability):

The code for this work is feasible and efficient.

Response to Reviewer #3

Reviewer #3 (Remarks to the Author):

This manuscript presents SUCCEED, a large-scale supervised multi-task DNA foundation model trained on 6,389 ENCODE tracks, integrating convolutional neural networks and Transformer architectures to jointly model local sequence motifs and long-range regulatory dependencies. Overall, the study is carefully designed, technically sound, and addresses an important problem in genomic sequence-to-function modeling.

Noteworthy contributions and significance:

The most notable contribution of this work is the demonstration that supervised pretraining on large-scale functional genomics data can yield foundation models with strong transferability across tasks, resolutions, and biological contexts, including epigenomic profile prediction, chromatin accessibility denoising, and 3D chromatin architecture inference. The comprehensive evaluation across bulk and single-cell ATAC-seq data, multiple cell types, and varying sequence lengths highlights the versatility and scalability of the proposed framework. The comparison with established methods such as Enformer, EPCOT, AtacWorks, and C. Origami is thorough and, in the revised version, appears fair and well controlled.

The work is clearly of relevance to the genomics and computational biology communities, particularly in the context of ongoing debates regarding supervised versus self-supervised foundation models for biological sequence modeling.

Soundness of methodology and evidence:

The methodology is generally sound, and the conclusions are well supported by extensive experimental evidence. The authors have substantially improved the manuscript by:

- Providing clearer architectural descriptions and motivations;

- Adding ablation studies to assess the impact of key architectural choices (e.g., SwiGLU, RMSNorm, rotary positional embeddings);

- Expanding benchmarking to include a broader set of foundation models under unified downstream decoders;

- Addressing reproducibility through repeated runs and reporting variability.

These additions significantly strengthen the technical rigor and transparency of the work.

Clarity and reproducibility:

The revised Methods section is detailed and largely sufficient to enable reproduction of the main experiments. Data preprocessing, normalization strategies, and training protocols are described clearly, and the inclusion of supplementary tables listing pretraining tracks and experimental configurations is particularly helpful.

Response: We sincerely thank the reviewer for the thorough evaluation and the highly positive assessment of our work. We greatly appreciate the careful reading of the revised manuscript and the recognition of the significance, technical soundness, and relevance of our study. We are pleased that the revisions have addressed the reviewer's concerns and strengthened the clarity, rigor, and reproducibility of the work.

Minor points and suggestions:

While the manuscript is already strong, a few minor clarifications could further improve clarity:

1. In the main text, it may be helpful to consistently emphasize when SUCCEED achieves comparable versus superior performance, particularly in tasks where differences with strong baselines (e.g., Sei or Enformer) are modest.

Response: We thank the reviewer for this helpful suggestion. We have revised the main text to more consistently distinguish between cases where SUCCEED achieves comparable versus superior performance, particularly in comparisons with strong baselines such as Sei and Enformer, where performance differences are modest.

2. A brief reminder in the main text that some comparisons are constrained by inherent architectural limits of baseline models (e.g., maximum input length) could help non-specialist readers interpret the benchmarks more carefully.

Response: We thank the reviewer for this helpful suggestion. We have added brief clarifications in both the main text and the Methods section noting that certain comparisons are constrained by inherent differences in the original tasks or architectural limitations of baseline models (such as maximum input sequence length), to help readers interpret the benchmarks more carefully.

3. The Discussion section appropriately outlines future directions (e.g., explicit multi-scale architectures, few-shot/meta-learning); these points could optionally be highlighted more concisely to avoid overextending the scope.

Response: We thank the reviewer for this suggestion. We have made minor edits to the

Discussion section to present the future directions more concisely.

Overall assessment:

In summary, this is a high-quality and timely study that makes a meaningful contribution to the field of genomic foundation models. The revised manuscript addresses prior concerns in a thorough and convincing manner, and I believe it is suitable for publication in Nature Communications.
